# Arylation with Unsymmetrical Diaryliodonium Salts: A Chemoselectivity Study

**DOI:** 10.1002/chem.201300860

**Published:** 2013-06-20

**Authors:** Joel Malmgren, Stefano Santoro, Nazli Jalalian, Fahmi Himo*, Berit Olofsson

**Affiliations:** [a] Department of Organic Chemistry, Arrhenius Laboratory, Stockholm University106 91 Stockholm (Sweden) Fax: (+46) 8-154908 E-mail: himo@organ.su.seberit@organ.su.se

**Keywords:** arylation, chemoselectivity, DFT calculations, hypervalent compounds, ligand exchange

## Abstract

Phenols, anilines, and malonates have been arylated under metal-free conditions with twelve aryl(phenyl)iodonium salts in a systematic chemoselectivity study. A new “*anti-ortho* effect” has been identified in the arylation of malonates. Several “dummy groups” have been found that give complete chemoselectivity in the transfer of the phenyl moiety, irrespective of the nucleophile. An aryl exchange in the diaryliodonium salts has been observed under certain arylation conditions. DFT calculations have been performed to investigate the reaction mechanism and to elucidate the origins of the observed selectivities. These results are expected to facilitate the design of chiral diaryliodonium salts and the development of catalytic arylation reactions that are based on these sustainable and metal-free reagents.

## Introduction

Hypervalent iodine compounds have emerged as selective and environmentally benign reagents in many areas of organic synthesis.1a,b Diaryliodonium salts (Ar_2_IX), also named diaryl-λ^3^-iodanes, serve as versatile electrophilic arylating agents with a variety of carbon and heteroatom nucleophiles under both metal-free and metal-catalyzed conditions.2c

Unsymmetrical diaryliodonium salts (Ar^1^≠Ar^2^) are required in enantioselective arylation reactions with chiral salts,3a as well as in the search for catalytic arylation conditions or polymer-bound salts.^4^ Furthermore, the synthesis of an unsymmetrical salt is often more facile than the corresponding symmetrical salt, because salts with one electron-poor and one electron-rich moiety are readily prepared, whereas very electron-rich or electron-poor symmetrical salts are more difficult to obtain.2a,^5^. Likewise, the synthesis of sterically hindered, symmetrical salts is cumbersome. Another advantage of unsymmetrical salts is seen if an expensive or non-commercial aryl group is to be transferred, because the waste of an expensive aryl-iodide moiety can be avoided.

However, the use of unsymmetrical diaryliodonium salts in arylation reactions requires control of the chemoselectivity to avoid transfer of the wrong aryl group onto the nucleophile. The factors that influence the observed chemoselectivities in metal-free reactions are poorly understood and a thorough chemoselectivity investigation with a systematic variation of both electronic and steric properties of the diaryliodonium salts has been lacking.

**Scheme 1:**
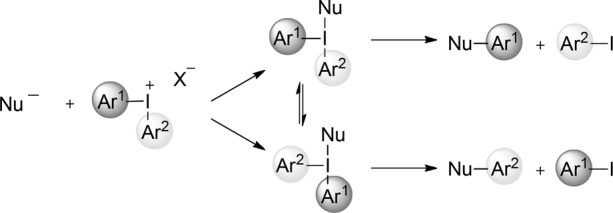
Chemoselectivity in metal-free arylation reactions with diaryliodonium salts.

It is generally accepted that diaryliodonium salts react with nucleophiles under metal-free conditions to form a T-shaped Ar_2_I–Nu intermediate, with the nucleophile and one of the aryl groups in the hypervalent three-center-four-electron (3c-4*e*) bond (Scheme 1). The reaction proceeds by ligand coupling (reductive elimination) between the nucleophile and the equatorial aryl group.6a Thus, reactions with unsymmetrical salts will give two T-shaped intermediates, which are in fast equilibrium through Berry pseudorotation.6e A direct nucleophilic aromatic substitution (S_N_Ar) at the *ipso*-carbon atom has also been suggested7a and a single electron transfer (SET) mechanism is likely under certain conditions.8a

Chemoselectivities that are due to electronic differences have been explained by a polarized ligand-coupling transition state, in which the developing charges are better stabilized by the substituents when the more electron-deficient group is transferred.[Bibr b6d][Bibr b6d] A recent computational study found a correlation between the chemoselectivity and the natural charge differences on the *ipso*-carbon atom of the aryl ligand in the hypervalent bond.6h On the other hand, the *ortho* effect has been rationalized by the bulkiest aryl group occupying the equatorial position in the T-shaped intermediate, because this position is less crowded than the apical position.6a Alternatively, a rate acceleration by the release of steric strain has been suggested, although assuming a different mechanism.6g

The α-arylation of β-dicarbonyl compounds with diaryliodonium salts has been well-investigated,6d, 9 including a theoretical study that showed two different T-shaped iodine(III) intermediates with a low isomerization barrier.^10^ Product formation through [2,3] rearrangement from the O–I intermediate was favored over [1,2] rearrangement from the C–I intermediate.^10^ Previous calculations on fluoride arylation with unsymmetrical heteroaryl salts have shown that chemoselectivity predictions must be based on relative transition-state (TS) energies rather than the stability of the T-shaped intermediates.^11^

Herein, we have performed a systematic study with three different types of nucleophiles to clarify the factors that influence the chemoselectivity and to find a suitable “dummy group” in metal-free arylation reactions with unsymmetrical iodonium salts. Further experiments have been performed to understand the reaction mechanism and to investigate an observed ligand exchange in the salts. Density functional theory (DFT) calculations have been carried out to shed more light on the origin of the observed selectivities. The results described herein will aid further mechanistic investigations and simplify the design of diaryliodonium salts that are suitable for asymmetric or catalytic processes.

## Results and Discussion

**Chemoselectivity studies**: In metal-free reactions, the most electron-deficient aryl group is generally transferred, as exemplified by the selective α-arylation of carbonyl compounds with 2-nitrophenyl(phenyl)iodonium salts[Bibr b12a][Bibr b12b] and various *p*-alkyl or *p*-methoxyphenyl(phenyl) salts.^9^ This selectivity can be overruled by steric bulk in the *ortho* position, thus leading to the selective transfer of that aryl group, irrespective of electronic properties (the so-called *ortho* effect).6a–c However, there are several examples of *ortho*-substituted aryl groups with various electronic properties that have not been transferred,3b, [Bibr b6d][Bibr b6e] thus making predictions and the design of unsymmetrical salts difficult.

Phenols and carboxylic acids are arylated with good chemoselectivity, which is either controlled by electronic factors or *ortho* substituents.13a The observed chemoselectivity with a certain salt can otherwise vary greatly with the type of nucleophile,2a, 8d, [Bibr b14a][Bibr b14b] as demonstrated in the arylation of aniline and fluoride with three different unsymmetrical salts.^15^

Fluoride arylation with unsymmetrical salts has been well-studied and gives good chemoselectivities based on electronic factors.[Bibr b16a][Bibr b16b][Bibr b16c][Bibr b16d][Bibr b16e] Few of the salts that are used in F-arylation have *ortho* substituents,7d, 17a but Pike and Widdowson have shown that *ortho* effects are indeed present.[Bibr b18a][Bibr b18b] DiMagno and co-workers recently designed cyclophane-derived diaryliodonium salts that gave good chemoselectivities with a range of nucleophiles.[Bibr b19a][Bibr b19b] However, these salts were synthesized in moderate yields over several steps and are therefore unattractive for application in standard arylation reactions.

Opposite trends in chemoselectivity are observed with diaryliodonium salts in the presence of a transition-metal catalyst. In this case, the more electron-rich or least bulky aryl group is generally transferred and the selectivity is easier to predict and control.2b

**Figure 1:**
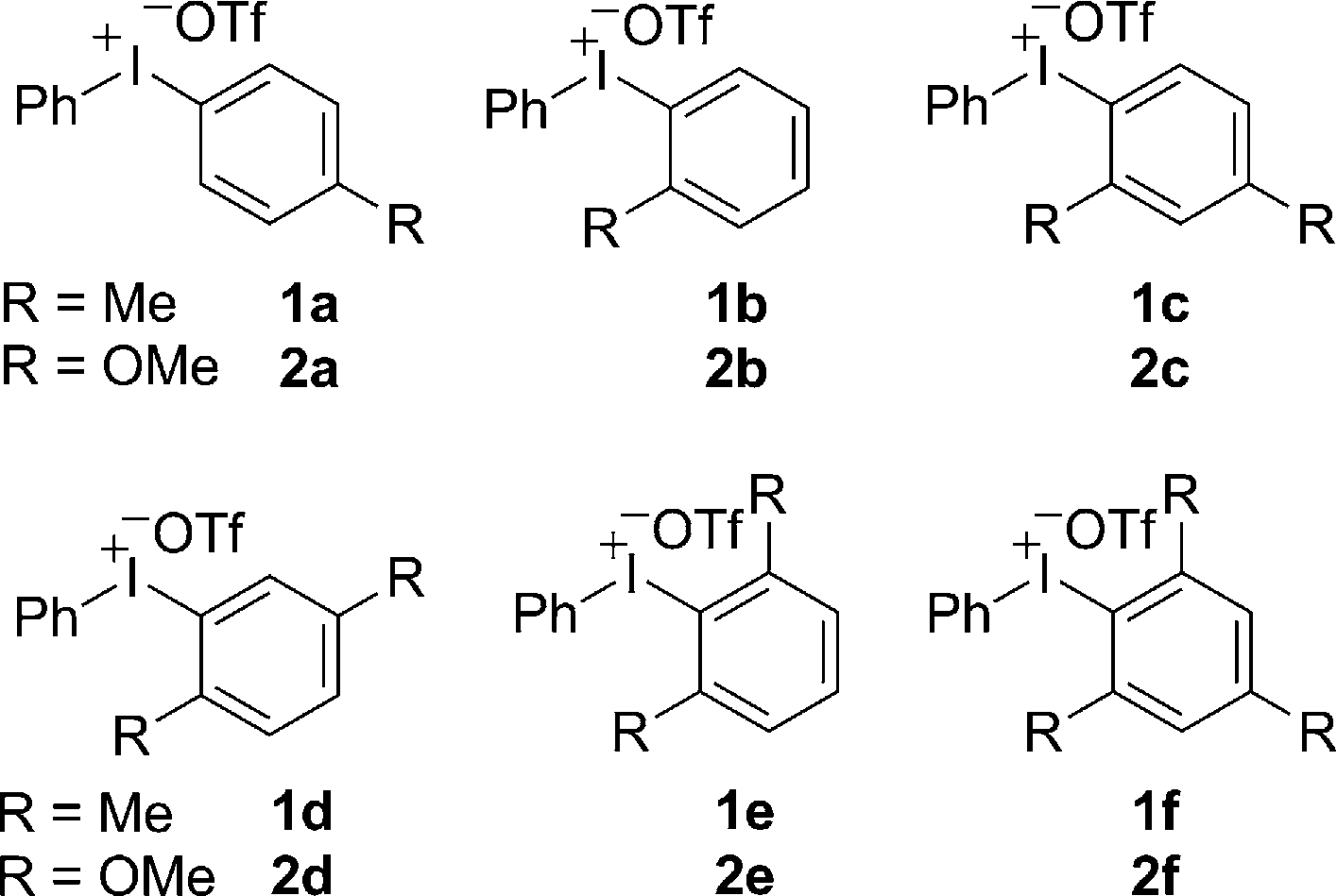
Unsymmetrical diaryliodonium triflates 1 and 2 that were used in the study.

We have designed twelve unsymmetrical diaryliodonium salts to study how variations in the steric bulk and electronic properties influence the arylation of nucleophiles under metal-free conditions (Figure 1).^20^ These salts contain one phenyl group and one aryl group with one, two, or three methyl (**1 a**–**1 f**) or methoxy substituents (**2 a**–**2 f**). The phenyl group is always the most electron deficient and should preferentially be transferred in the absence of other effects (salts **1 a** and **2 a**). Salts **1 b**–**2 f** and **2 b**–**2 f** have *ortho* substituents that could influence the chemoselectivity towards the transfer of the aryl group instead of the phenyl group, according to the *ortho* effect.

**Scheme 2:**
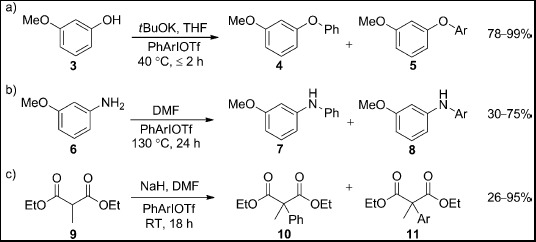
Arylation conditions for: a) phenols,13b b) anilines,^15^ and c) malonates.^9^

Phenol **3**, aniline **6**, and malonate **9** were selected to study the phenylation versus arylation of O, N, and C nucleophiles under previously reported conditions (Scheme 2).^9^, 13b, 15 The combined yields of the isolated phenylated and arylated products are given in Scheme 2; they were consistently high in the synthesis of diaryl ethers, but varied considerably with the salt for the other nucleophiles. 3-Methoxy substituents were used on the phenol and aniline substrates to facilitate NMR determination of the product ratios, which are listed in Table 1 and Table 2.

**Table 1:** Phenylation versus arylation of 3, 6, and 9 with iodonium salts 1.^[a]^

		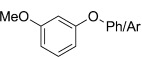		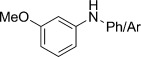		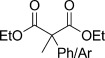	
		Yield4/5[%]		Yield7/8[%]		Yield10/11[%]	
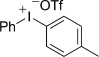	**1 a**	2.9:1	78	1.4:1	53	3.3:1	54
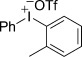	**1 b**	1:2.4	98	1.4:1	40	11:1	64
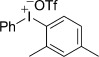	**1 c**	1.3:1	98	4.5:1	75	5.0:1	52
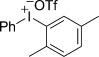	**1 d**	1:1.6	>99	2.5:1	60	2.0:1	95
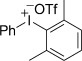	**1 e**	1:9	98	6.5:1	53	only **10**	26
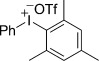	**1 f**	1:1.9	84	15:1	50	only **10**	55

[a] Combined yields of the isolated products. Product ratios are taken from ^1^H NMR spectroscopy of the combined isolated products for compounds **4**/**5** and from crude mixtures for compounds **7**/**8** and **10**/**11**.

**Table 2:** Phenylation versus arylation of 3, 6, 9 with iodonium salts 2.^[a]^

		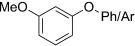		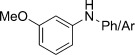		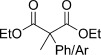	
		Yield4/5[%]		Yield7/8[%]		Yield10/11[%]	
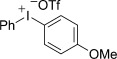	**2 a**	only **4**	94	5.4:1	74	13:1	36
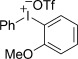	**2 b**	only **4**	93	3.0:1	62	2.6:1	61
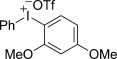	**2 c**	only **4**	82	only **7**	50	only **10**	53
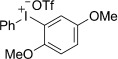	**2 d**	only **4**	90	2.3:1	30	only **10**	57
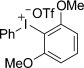	**2 e**	only **4**	>99	only **7**	70	only **10**	38
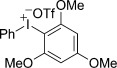	**2 f**	only **4**	85	only **7**	45	only **10**	44

[a] Combined yields of the isolated products. Product ratios are taken from ^1^H NMR spectroscopy of the combined isolated products for compounds **4**/**5** and from crude mixtures for compounds **7**/**8** and **10**/**11**.

The arylation reactions of phenol **3** with methyl-substituted salts **1** closely follow the two trends described above (Table 1): salt **1 a** gives a 3:1 preference for phenylation, in line with the electronic effects, whereas salt **1 b** follows the *ortho* effect and salts **1 c** and **1 d** give unselective reactions, owing to the two factors opposing each other. The different results with salts **1 c** and **1 d** are in accordance with the *p*-Me substituent being more electron donating than a *m*-Me substituent.^21^ Salt **1 e** shows a strong *ortho* effect, whereas the poor selectivity with trisubstituted salt **1 f** is a combination of the results with salts **1 a** and **1 e**, as expected.

In contrast, the reactions with aniline **6** gave preferential phenylation to afford compound **7** in all cases, thus indicating that the *ortho* effect is not present for this nucleophile. One methyl substituent gives a slight selectivity (**1 a** and **1 b**), which is increased with two methyl substituents (**1 c**–**1 e**), and mesityl salt **1 f** gives high selectivity for compound **7**.

The results that were obtained with malonate **9** are more difficult to rationalize, because salts **1** all preferentially give the phenylation product, but the results are not additive, that is, salts **1 c** and **1 d** give lower selectivities than salt **1 b**, despite them containing another electron-donating substituent. In fact, one *ortho* substituent gives a **10**/**11** ratio of 11:1 (**1 b**) and two *ortho* substituents give complete selectivity for the phenylation product (**1 e** and **1 f**). This trend has not previously been reported and can be termed an “*anti*-*ortho* effect”.

Methoxy-substituted salts **2** were completely chemoselective for the phenylation of phenol **3** to yield compound **4**, irrespective of the substitution pattern (Table 2). The electronic effect apparently overrides the steric effect completely in the reactions with compound **3**. Arylation of aniline **6** proceeded with moderate selectivity with monosubstituted salts **2 a** and **2 b**, thus matching the trends seen with salts **1**. Complete selectivity for product **7** was observed with salts **2 c**, **2 e**, and **2 f**, whereas salt **2 d** only gave a 2.3:1 ratio. The different outcomes with salts **2 c** and **2 d** are in accordance with the switch of an electron-donating *p*-OMe substituent for an electron-withdrawing *m*-OMe substituent.^21^

The reactions between malonate **9** and monosubstituted salts **2 a** and **2 b** indicate the presence of an *ortho* effect, which counteracts the electronic effect that is exerted by the methoxy group, because the selectivity is much higher with *para*-substituted salt **2 a** than with *ortho*-substituted salt **2 b**. This result is unexpected, because the opposite effect was seen with the methyl-substituted salts (Table 1). Disubstituted salts **2 c**–**2 e** and trisubstituted salt **2 f** all gave complete selectivity for the phenylation reaction to afford compound **10**. Surprisingly, our results with 3-methoxyaniline (**6**) differ from those that were previously reported with aniline and salts **1 f** and **2 a** with a trifluoroacetate anion instead of a triflate anion.^15^, ^22^ The results with malonate **9** matched previously reported values in terms of the product ratios, although lower yields were consistently observed.^9^, ^23^

To summarize the chemoselectivity results, any methoxy-substituted aryl moiety (as in **2**) can be used as a dummy ligand to control the chemoselectivity in the arylation reactions of compound **3**, whereas the di- or trimethoxy aryl groups in salts **2 c** and **2 f** are the most convenient dummy ligands for chemoselective transfer with compounds **6** and **9**. Salts that contain a 2,6-dimethoxy group (e.g., **2 e**) also give complete chemoselectivity, but are more difficult to synthesize.

In this study, the phenyl group was the most electron-deficient group in all of the salts that were used. Based on previous reports, electron-withdrawing aryl groups are expected to be transferred with similar or higher chemoselectivities than the phenyl group. Reactions with salts that contain two different electron-donating aryl groups are predicted to give lower chemoselectivities, but follow the trends that were observed in this study.

**Aryl exchange**: DiMagno and co-workers recently reported an unexpected aryl exchange in F-arylation with unsymmetrical diaryliodonium salts, thus giving symmetrical diaryliodonium salts in the presence of the nucleophile [Eq. (1)].^17^


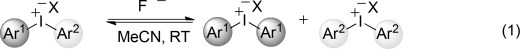
(1)

Intrigued by this possibility, we investigated whether an aryl exchange also took place in our reactions. Salt **2 d** was selected as test substrate, because it had given low chemoselectivity with aniline **6** (2.3:1). When salt **2 d** was stirred in DMF at room temperature or heated at reflux in the absence of a nucleophile, no ligand exchange was seen within 1 h, according to HRMS analysis of the reaction mixture. Moreover, when aniline **6** was reacted with salt **2 d** under the normal arylation conditions, no exchange was seen and salt **2 d** remained as the only diaryliodonium species throughout the reaction.

**Scheme 3:**
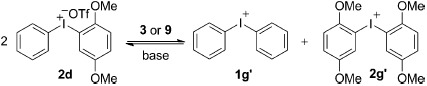
Diaryliodonium cations that were observed in the reactions of compounds 3 and 9 with salt 2 d.

In stark contrast, the reaction of phenol **3** with salt **2 d** produced HRMS peaks that corresponded to symmetrical diaryliodonium cations **1 g′** and **2 g′** within 5 min reaction time at room temperature (Scheme 3). In principle, these species could influence the chemoselectivity in both directions, because only one aryl group can be transferred from the symmetrical salts that are formed in situ. The theoretical phenylation yield is decreased if arylation takes place with cation **1 g′**, because the formation of iodobenzene wastes one phenyl moiety that could otherwise be transferred. Since product **4** was obtained in 90 % yield with complete chemoselectivity, the arylation must have mainly taken place with salt **2 d**, despite the presence of other salts, thus indicating that the aryl exchange is reversible.

Furthermore, the reaction between salt **2 d** and malonate **9** showed aryl exchange to cations **1 g′** and **2 g′** within 5 min reaction time at room temperature. Since product **10** was obtained in 57 % yield with complete chemoselectivity, cation **2 g′** must be very unreactive compared to salt **2 d** and cation **1 g′**. The lower theoretical yield if arylation takes place with cation **1 g′** (see above) could explain the lower yields seen throughout this study compared to the arylation of compound **9** with Ph_2_IOTf (80 %).

The aryl exchange was further investigated with malonate **9** by using 2,5-dimethyl-substituted salt **1 d**, and the symmetrical di(2,5-dimethylphenyl)iodonium cation (**2 h′**) and diphenyliodonium cation (**1 g**) were also detected in this reaction. The chemoselectivity was followed by NMR spectroscopy throughout the reaction and only changed slightly over time (from 2.1:1 to 1.9:1 over 3 h). This result either indicates that thearylation rates with the three salts are rather similar or that the aryl exchange is in fast equilibrium. A control experiment in which compound **9** was reacted with the symmetrical salts **1 h** and di(*p*-methoxyphenyl)iodonium triflate (**2 h**) showed that the aryl exchange took place rapidly to form the corresponding unsymmetrical cation, **2 i′**. On the contrary, no aryl exchange was detected in a control experiment in which compound **9** and a di(*p*-tolyl)iodonium triflate (**1 i**) were reacted in the presence of 2,6-dimethyliodobenzene, thus demonstrating that iodine(I) is not involved in the exchange reaction.

The reactions with compounds **3** and **9** are both performed at low temperatures with deprotonated nucleophiles, whereas the arylation of compound **6** takes place at high temperatures without added base; in fact, the addition of a base is detrimental to the reaction. The absence of aryl exchange in the reactions with aniline **6** might be explained by the aniline remaining protonated in the iodine(III) intermediate. Then, it would exchange much more easily than the anions of compounds **3** and **9**, thus making exchange of the aryl ligands more unlikely. Alternatively, the arylation mechanism could be different for compound **6** compared to compounds **3** and **9**. Therefore, a mechanistic study was performed, as detailed below.

**Mechanistic studies**: To shed more light on the origin of the chemoselectivity, we performed DFT calculations on selected substrates. The transition-state energies for ligand coupling from the two T-shaped isomers were calculated and compared and, in general, very good agreement was found with the experimentally observed selectivities (Table 3).

**Table 3:** Calculated and experimentally observed differences in ligand-coupling transition-state energies [kcal mol^−1^].

Entry	Nucleophile	Salt	Exptl Ph/Ar ratio^[a]^	Exptl^[b]^ ΔΔ*G*^≠^	Calcd^[c]^ ΔΔ*G*^≠^
1	**3**	**1 a**	2.9:1	−0.6	−0.5
2	**3**	**1 e**	1:9	+1.3	+0.9
3	**6**	**1 a**	1.4:1	−0.3	−0.5
4	**6**	**2 b**	3.0:1	−0.9	−1.0
5	**9**^[d]^	**1 d**	2.0:1	−0.4	−0.4
6	**9**^[d]^	**1 e**	only Ph	<−1.8^[e]^	−2.0

[a] Ratios taken from Tables 1 and 2. [b] Differences in reaction barriers as converted from the observed chemoselectivities based on the Eyring equation. The temperatures of the experiments (Scheme 2) were taken into account during the conversion of the selectivities. [c] Difference in the Gibbs free energies between the lowest-energy transition states that lead to the two products. Room temperature was used for nucleophiles **3** and **9**, whereas 130 °C was used for nucleophile **6**. [d] Diethyl methylmalonate was modeled as dimethyl methylmalonate. [e] A difference of 1.8 kcal mol^−1^ corresponds to a 95:5 ratio.

The calculated energy differences between the transition states that lead to the two different products can only be directly compared under Curtin–Hammett conditions, that is, when the isomerization between the two T-shaped intermediates is faster than the ligand-coupling step. Indeed, the calculated barrier for isomerization in one representative reaction (between salt **1 a** and phenol **3**) was much lower than the barriers for the two possible ligand-coupling reactions (11.8 vs. 17.0 kcal mol^−1^, as the lowest barrier for ligand coupling). This result is in agreement with the results of previous calculations.6h, 10, 11

**Figure 2:**
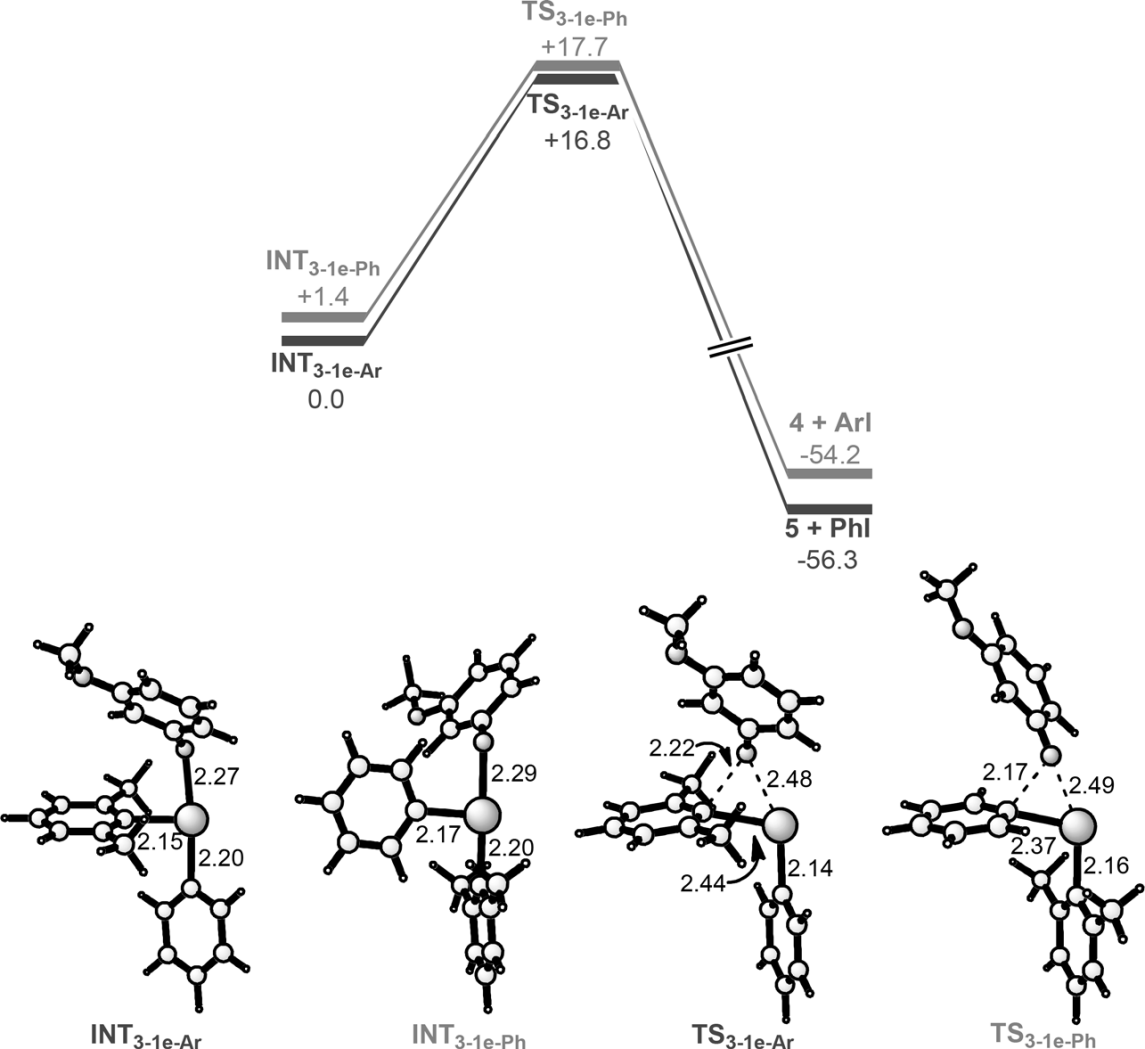
Free-energy profiles and optimized structures of starting T-shaped complexes and transition states in the reaction of phenol 3 with salt 1 e. Distances are in Å and energies in kcal mol^−1^. This Figure is provided in color in the Supporting Information.

The calculations very nicely reproduced the observed experimental selectivities in all of the cases that were investigated. For example, the reaction of iodonium salt **1 a** with phenol **3** experimentally gives a low chemoselectivity, which corresponds to a barrier difference of about 0.6 kcal mol^−1^ (Table 3, entry 1). The calculated barriers for the phenyl and tolyl transfers are 17.0 and 17.5 kcal mol^−1^, respectively, thus giving a difference of 0.5 kcal mol^−1^. On the other hand, the reaction between compound **3** and salt **1 e** leads to preferential transfer of the more electron-rich *ortho*-disubstituted aryl group, which corresponds to a difference of 1.3 kcal mol^−1^ (Table 3, entry 2). The calculated difference in TS energies is 0.9 kcal mol^−1^ (Figure 2).

**Figure 3:**
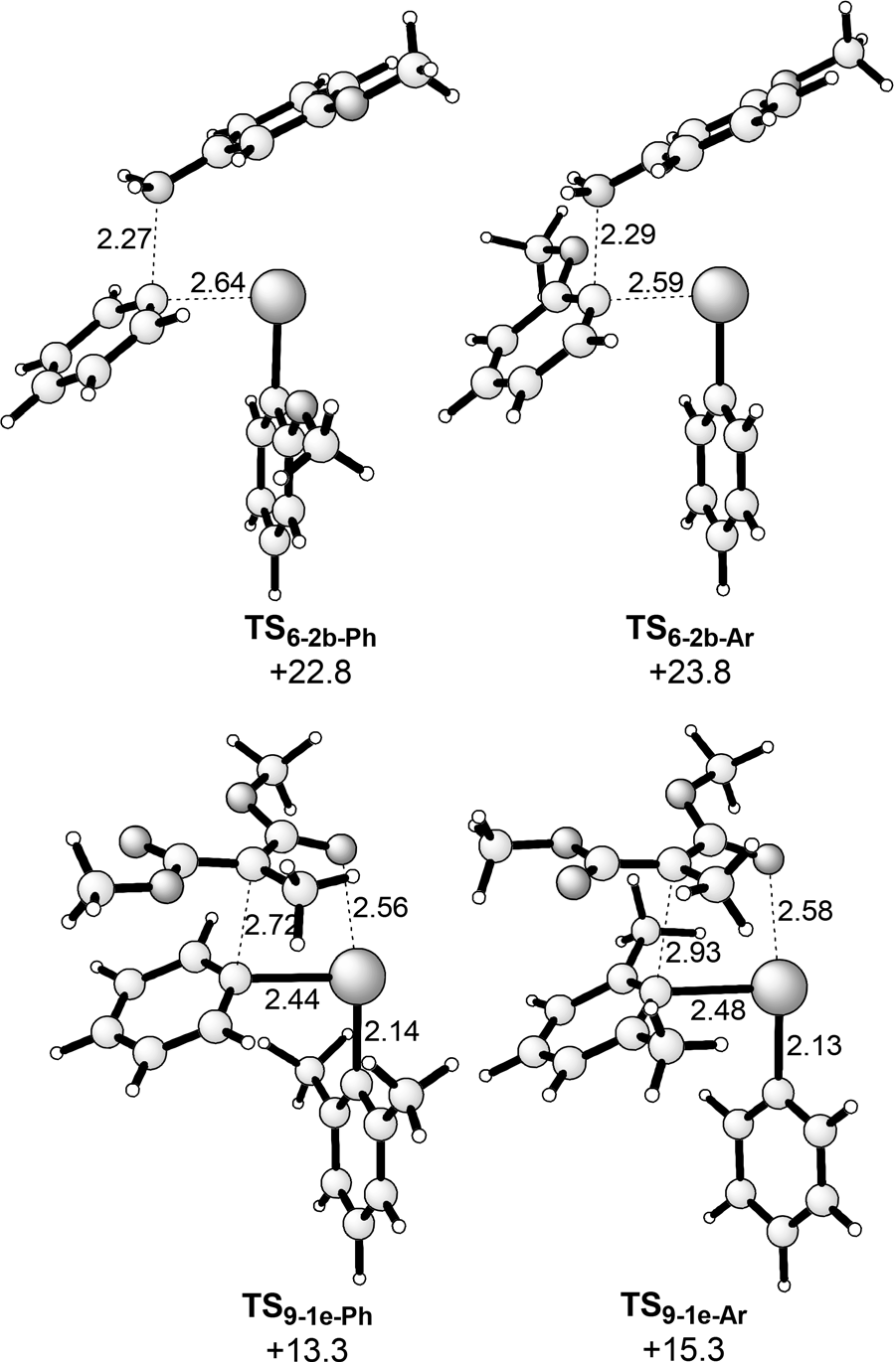
Optimized TS structures in the reaction of aniline 6 with salt 2 b and the reaction of malonate 9 with salt 1 e. Distances are in Å and energies in kcal mol^−1^. This Figure is provided in color in the Supporting Information.

Aniline **6** was arylated without the addition of a base and at a much higher temperature (130 °C) than the other nucleophiles. The reaction with salt **1 a** gave a low selectivity (Ph/Ar 1.4:1), which corresponds to a barrier of about 0.3 kcal mol^−1^ at that temperature (Table 3, entry 3); in this case, the energy difference between the calculated transition states was 0.5 kcal mol^−1^. Similarly, the reaction of compound **6** with salt **2 b** favored the transfer of the least electron-rich aryl group (Table 3, entry 4). In this case, the selectivity was slightly higher and this result was also quite accurately reproduced by the calculations (Figure 3).

Finally, we also modeled the reactivity of malonate **9**, for which the experimental results with salts **1** were somewhat surprising. In the reaction between compound **9** and salt **1 d**, which only has one *ortho*-methyl group, the selectivity is only 2:1 (Table 3, entry 5). This result is reproduced by the calculations (calculated difference of 0.4 kcal mol^−1^). The reaction with salt **1 e** gives complete selectivity for phenylation, despite the presence of two *ortho* substituents (Table 3, entry 6). The calculations give a difference of 2.0 kcal mol^−1^ in favor of the phenylated product, in good agreement with the experimental data (Figure 3). Note that, although the aryl group occupies the equatorial position in the T-shaped intermediate, the TS for the phenyl transfer is lower in energy than the TS for the aryl transfer (for the free-energy profile of this reaction, see the Supporting Information). Thus, this result confirms the above-stated fact that chemoselectivity predictions must be based on the relative transition-state energies rather than on the relative energies of the T-shaped intermediates.^11^

The calculations reproduced the experimental chemoselectivities very well, thus providing further support for the established ligand-coupling mechanism. It is also clear from the calculations that the chemoselectivity depends on a delicate balance between electronic and steric effects. In the cases when only *para* substituents are present on the aryl moiety, such as the reactions between salt **1 a** and nucleophiles **3** and **6**, there are no steric effects present and, thus, the chemoselectivity is only dictated by electronic effects.

**Figure 4:**
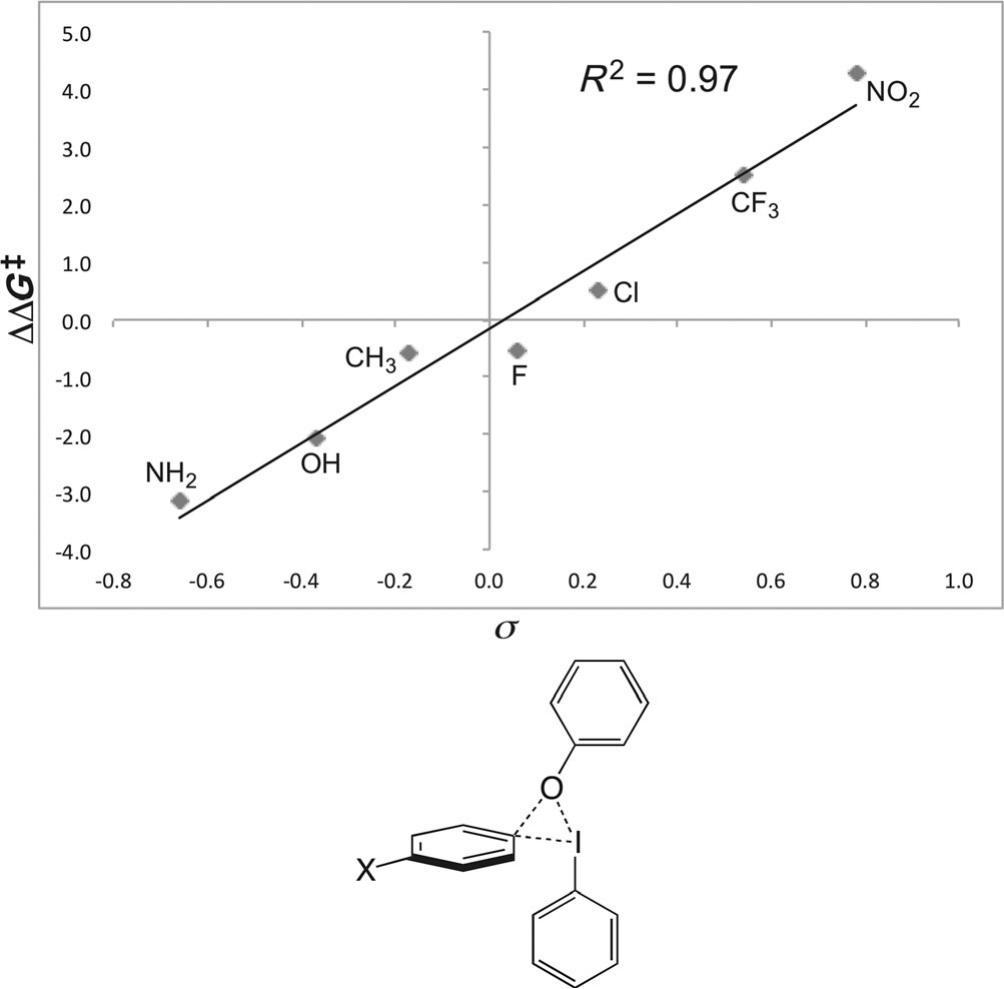
Plot of the calculated relative energy barriers of phenyl/aryl transfer versus the Hammett *σ* values for the ligand coupling between phenoxide and an aryl(phenyl)iodonium salt with one *para*-substituted aryl group.

To further assess the dependence of chemoselectivity on electronic effects, we studied the ligand coupling between phenoxide and aryl(phenyl)iodonium salts with seven different *para* substituents. The calculated differences between the energy barriers for phenyl or aryl transfer were correlated with the Hammett *σ* value of the substituent (Figure 4).^21^ A good correlation was found, which showed that the more electron-withdrawing the substituent, the higher the chemoselectivity in favor of aryl transfer.

To analyze the source of this electronic effect, we plotted the absolute barriers for ligand coupling between the phenoxide and the phenyl/aryl groups as a function of the Hammett *σ* value (see the Supporting Information). A good correlation was found for the aryl transfer, whereas no correlation was found for the phenyl transfer. This suggests that the substituent influences the reaction barrier and, thus, the chemoselectivity by stabilizing or destabilizing the attack of the nucleophile on the *ipso*-carbon atom, rather than influencing the properties of the 3c-4*e* bond.

As mentioned above, a previous theoretical study showed that the differences in natural charge between the *ipso*-carbon atom of the aryl ligands in the 3c-4*e* bond correlated well with the calculated selectivity for *meta*- and *para*-substituted aryl compounds, whereas aryl ligands with *ortho* substituents did not follow the same pattern.[Bibr b6h][Bibr b6g] It was also suggested that the polarity of the 3c-4*e* bond correlated with experimental Hammett *σ* parameters.6h These findings can be reconciled with our results by simply recognizing that the charge on the *ipso*-carbon atom indirectly reflects the inductive effect of the substituent. Therefore, we believe that the use of the Hammett *σ* correlation is a more straightforward measure.^24^

**Discussion**: The reaction between compound **3** and iodonium salt **1 e**, which contains *ortho*-methyl groups, is a clear example of the so-called *ortho* effect. With this nucleophile, the steric effect that is exerted by the methyl groups overrides their electron-donating properties, which would favor phenyl transfer. The *ortho* effect is normally explained by assuming that the bulkiest aryl groups prefer the equatorial position over the apical position, because, in trigonal bipyramidal geometries, the latter positions are more crowded. Our calculations show that there is indeed already a preference (1.4 kcal mol^−1^) for the xylyl group occupying the equatorial position in the T-shaped intermediate. However, it should be noted that the TSs show considerable distortion compared to the starting geometries (Figure 2) and, thus, other effects could play a role in dictating the selectivity. Analysis of the structures of the two transition states (**TS_3-1e-Ar_** and **TS_3-1e-Ph_**, Figure 2) shows that the breaking I–C bond is significantly elongated and that this elongation is more pronounced when the aryl group is transferred (2.44 versus 2.37 Å). It could also be possible that there is a release of the steric repulsion between the *ortho*-methyl groups and the iodine atom in the TS that leads to the transfer of the substituted aryl group and that this can contribute to the lower energy of this TS compared to the one that leads to phenyl transfer.

The arylation of malonate **9** is a special case, because a new “*anti*-*ortho* effect” is observed with the methyl-substituted salts. A comparison of **TS_9-1e-Ph_** with **TS_9-1e-Ar_** (Figure 3) gives valuable insight into the origin of this effect. Indeed, the forming C–C bond is much longer in **TS_9-1e-Ar_** than in **TS_9-1e-Ph_** (2.93 vs. 2.72 Å), thus suggesting that this “*anti*-*ortho* effect” is due to steric repulsion between the bulky aryl group and the malonate, which results in a preferential transfer of the phenyl group. The same effect is present, although to a lesser extent, in the arylation of compound **9** with iodonium salt **1 d**.

Finally, we also considered the possibility of a mechanism that involved a direct nucleophilic attack on the *ipso*-carbon atom by an external nucleophile (i.e., a nucleophile that is not a ligand on the hypervalent iodine).[Bibr b7a][Bibr b7b][Bibr b7c] For all of the three nucleophiles, the calculated barriers were found to be higher for this mechanistic proposal compared to the barrier to the direct ligand-coupling mechanism as discussed above (data not shown).

However, these calculations cannot completely exclude the involvement of a radical mechanism, at least under certain reaction conditions. Experimentally, the arylation reactions of both phenols^13^ and malonate **9** were found to be insensitive towards the radical scavenger diphenylethylene (DPE) in their reactions with diaryliodonium triflates. On the other hand, the arylation of aniline **6** was affected by radical scavengers,^15^ because the addition of DPE or (2,2,6,6-tetramethylpiperidin-1-yl)oxidanyl (TEMPO) considerably lowered the yield of the arylation reactions with both salts **1 a** and **2 b**, without altering the chemoselectivity.

The accurate calculated reproduction of the observed selectivities gives indirect support to the ligand-coupling mechanism, although the factors that govern the reaction outcome in the ligand-coupling mechanism studied herein could be the same as those that dictate the selectivity in a hypothetical radical mechanism.

The mechanism of aryl exchange remains unknown and will be the subject of further studies.

## Conclusion

The observed chemoselectivities vary considerably with the type of nucleophile. The reactions of phenol **3** with methyl-substituted salts **1** are influenced by both electronic and *ortho* effects, whereas only electronic factors are important with more electron-rich methoxy salts **2**. The selectivities that were obtained with aniline **6** were rather different from those with phenol **3**, as only the electronic properties influenced the outcome and phenylation was always the major pathway. Malonate **9** showed a clear “*anti-ortho* effect” with the methyl-substituted salts **1**, thus resulting in opposite chemoselectivity to phenol **3**. In contrast, the reactions of compound **9** with methoxy-substituted salts **2** were influenced by both electronic and *ortho* effects, although the electronic effects dominated. The di- or trimethoxy aryl groups on salts **2 c** and **2 f** are the most convenient dummy ligands, because complete chemoselectivity was observed with all nucleophiles.

A fascinating aryl-exchange was observed in the reactions with phenol **3** and malonate **9**, resulting in the formation of two symmetrical diaryliodonium cations. The high chemoselectivities and good yields that were obtained in these reactions indicate a large difference between the reactivities of the three diaryliodonium species under these reaction conditions.

DFT calculations showed very good agreement with the experimental selectivities and confirmed that electronic effects favored the transfer of the most electron-poor aryl group. This effect does not depend on an influence on the 3c-4*e* bond, but rather on the stabilization of the nucleophilic attack on the *ipso*-carbon atom of the equatorial aryl group in the ligand-coupling step. The *ortho* effect is based on steric factors and is more difficult to account for because it varies with the nucleophile. The “*anti*-*ortho* effect” observed in the arylation of malonate **9** depends on the steric repulsion between the bulky ligand and the nucleophile.

## Experimental Section

**Arylation of phenol 3**:13b To a suspension of *t*BuOK (1.1 equiv, 43 mg, 0.37 mmol) in THF (1.5 mL) was added phenol **3** (1.0 equiv, 0.34 mmol) at 0 °C and the reaction was stirred at this temperature for 15 min. Diaryliodonium salt **1** or **2** (1.2 equiv, 0.40 mmol) was added in one portion and the reaction was stirred in an oil bath that had been preheated to 40 °C until salt **1** or **2** had been completely consumed (by TLC). Then, the reaction was quenched with water at 0 °C, the organic phase was separated, and the water phase was extracted with Et_2_O (3×10 mL). The combined organic phases were dried (Na_2_SO_4_) and concentrated in vacuo. The crude material was purified by flash chromatography on silica gel to give diaryl ethers **4** and **5** as an inseparable mixture. The product ratio was determined by ^1^H NMR spectroscopy of the isolated mixture.

**Arylation of aniline 6**:^15^ Diaryliodonium salt **1** or **2** (1 equiv, 0.25 mmol) was dissolved in dry DMF (2 mL) and *m*-anisidine (1 equiv, 0.028 mL, 0.25 mmol) was added under stirring at RT. The reaction mixture was placed in an oil bath that had been preheated to 130 °C and stirred for 24 h. The reaction was treated with a 1 m aqueous solution of Na_2_CO_3_ (2 mL), extracted with EtOAc (3×5 mL), and washed with water (1×10 mL) and brine (2×10 mL). The combined organic phases were dried (MgSO_4_) and concentrated in vacuo. The crude material was purified by flash chromatography on silica gel to give diarylamines **7** and **8** as an inseparable mixture. The product ratio was determined by ^1^H NMR spectroscopy from the crude mixture.

**Arylation of malonate 9**:^9^ NaH (60 % dispersion in mineral oil, 1.3 equiv, 0.33 mmol, 10 mg) was suspended in DMF (0.5 mL) and diethyl methylmalonate (**9**, 0.25 mmol, 44 mg) was added dropwise at 0 °C. The reaction was stirred at RT for 10 min. A solution of diaryliodonium salt **1** or **2** (1.3 equiv, 0.33 mmol) in DMF (0.5 mL) was added by cannula to the reaction mixture at 0 °C. The reaction mixture was stirred at RT until compound **9** had been completely consumed (by TLC). The reaction was quenched with water at 0 °C, extracted with EtOAc (3×5 mL), and washed with water (1×10 mL) and brine (2×10 mL). The combined organic phases were dried (MgSO_4_) and concentrated in vacuo. The crude material was purified by flash chromatography on silica gel to give products **10** and **11** as an inseparable mixture. The product ratio was determined by ^1^H NMR spectroscopy of the crude mixture.
