# Supplementary material for: Arylation with Unsymmetrical Diaryliodonium Salts: A Chemoselectivity Study
Source: Chemistry. 2013 Jun 20;19(31):10334–42. doi: 10.1002/chem.201300860 (PMC3884774; doi:10.1002/chem.201300860)

CHEMISTRY
A EUROPEAN JOURNAL

Supporting Information

© Copyright Wiley-VCH Verlag GmbH & Co. KGaA, 69451 Weinheim, 2013

Arylation with Unsymmetrical Diaryliodonium Salts: A Chemoselectivity Study

Joel Malmgren, Stefano Santoro, Nazli Jalalian, Fahmi Himo,* and Berit Olofsson*[a]

chem_201300860_sm_miscellaneous_information.pdf

Arylation with Unsymmetric Diaryliodonium Salts – a Chemoselectivity Study

Joel Malmgren, Stefano Santoro, Nazli Jalalian, Fahmi Himo* and Berit Olofsson*

Department of Organic Chemistry, Arrhenius Laboratory, Stockholm University, SE-106 91 Stockholm (Sweden)
himo@organ.su.se, berit@organ.su.se

SUPPORTING INFORMATION

1. General Experimental Conditions S2
2. Mechanistic Studies S2
3. Synthesis of Diaryliodonium Salts **1**, **2, 12** S6
4. Arylation of Phenol **3** to Products **4**, **5** S9
5. Arylation of Aniline **6** to Products **7**, **8** S10
6. Arylation of Malonate **9** to Products **10**, **11** S13
7. Computational Figures and Details S16
8. Copies of ^{1}H and ^{13}C NMR Spectra S42

1 General Experimental Conditions

Precautions to exclude air or moisture were not taken, except when mentioned. Commercial *m*CPBA was dried under vacuum at rt for 1 hour and subsequently the percentage of active oxidising reagent was determined by iodometric titration.[1] All other commercially available chemicals were used as supplied. For TLC analyses precoated silica gel 60 F_{254} plates were used; and for column chromatography 40-60 µm, 60A silica gel was used. Melting points were measured using a STUART SMP3 and are reported uncorrected. NMR spectra were recorded using a 400 MHz Bruker AVANCE II with a BBO probe at 298 K, unless otherwise mentioned, using $CDCl_3$ and DMSO-d_6 as solvents. Chemical shifts are given in ppm relative to the (residual) solvent peak (^{1}H NMR: $CHCl_3$ δ 7.27, DMSO-d_5 δ 2.50; ^{13}C NMR: $CDCl_3$ δ 77.23, DMSO-d_6 δ 39.52) with multiplicity (br=broad, s=singlet, d=doublet, t=triplet, q=quartet, m=multiplet, app=apparent), coupling constants (in Hz) and integration. High resolution mass analyses were obtained using a Bruker microTOF ESI with a time-of-flight-detector. Combined isolated yield refers to calculated yield based on the isolated mass of the two products and their NMR integrals.

2 Mechanistic studies

2.1 Aryl exchange study by HRMS

The aryl exchange studies were performed by setting up reactions according to the experimental protocols for arylation of **4**, **5** and **6** (see the paper or Sections 5-7). A sample was taken *via* syringe and immediately dissolved in a mixture of MeOH and H_2O (10 mL 1:1). This mixture was then further diluted (by a factor of 10) before analysis. The samples were taken after 5 min, 30 min and then once every hour up to ten hours unless otherwise noted.

Experiments with 2,5-dimethoxyphenyl-(phenyl)iodonium triflate (2d):

* Without nucleophile: **2d** (0.25 mmol) was stirred in DMF at rt and under reflux. No aryl exchange was detected.

* With 3-methoxy phenol **3**: Samples were taken after 5 min and 30 min respectively. The di(2,5-dimethoxyphenyl)iodonium cation **2g'** and diphenyliodonium cation **1g'** were detected after 5 min reaction time at room temperature (Figure S1).

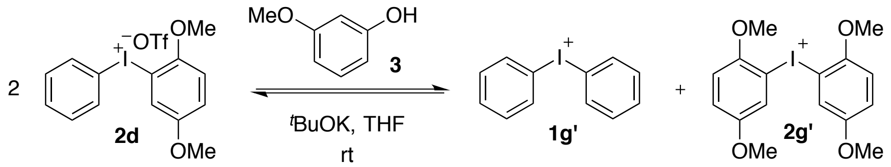

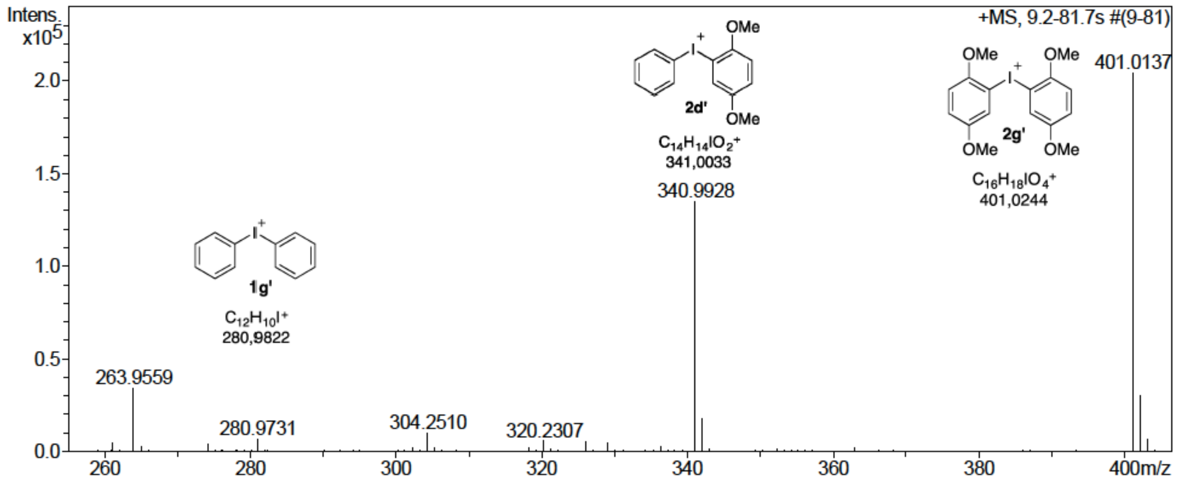

Figure S1. HRMS of aryl exchange after 5 min reaction of phenol **3** with salt **2d**.

* With 3-methoxy aniline **6**: No aryl exchange was detected.

* With diethylmethyl malonate **9**: The di(2,5-dimethoxyphenyl)iodonium cation **2g’** and diphenyliodonium cation **1g’** were detected. (Figure S2).

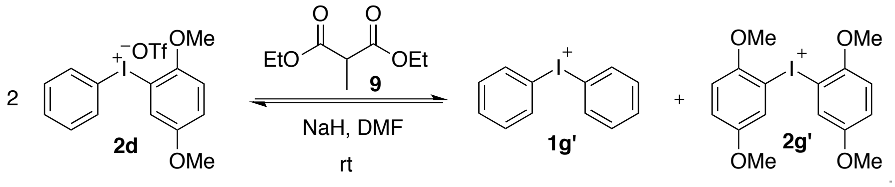

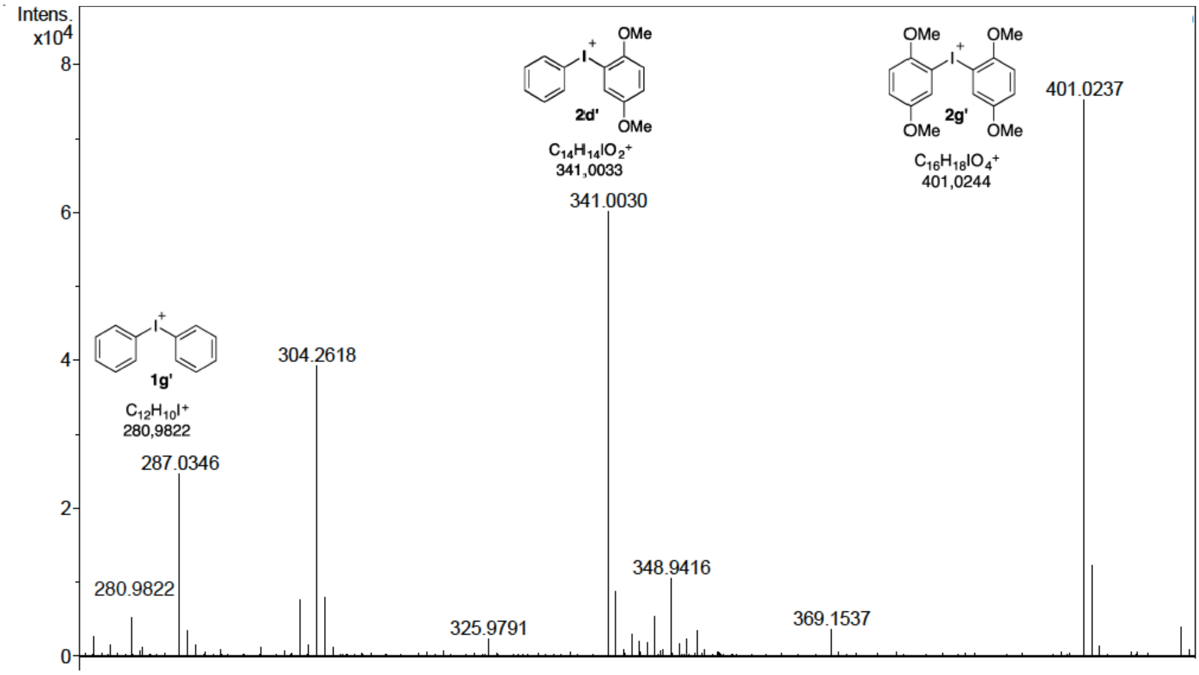

Figure S2. HRMS of aryl exchange after 5 min reaction of malonate **9** with salt **2d**.

Experiments with 2,5-dimethylphenyl-(phenyl)iodonium triflate (1d):
* With diethylmethyl malonate (**9**): The diphenyliodonium cation **1g'** and di(2,5-dimethylphenyl)iodonium cation **1h'** were detected after 5 min at room temperature (Figure S3).

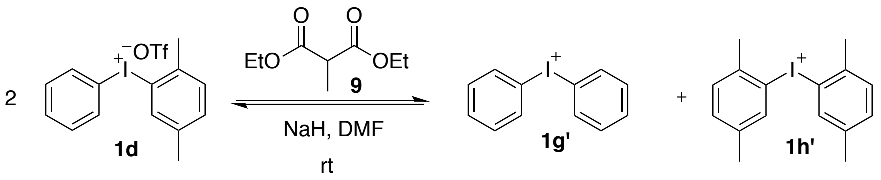

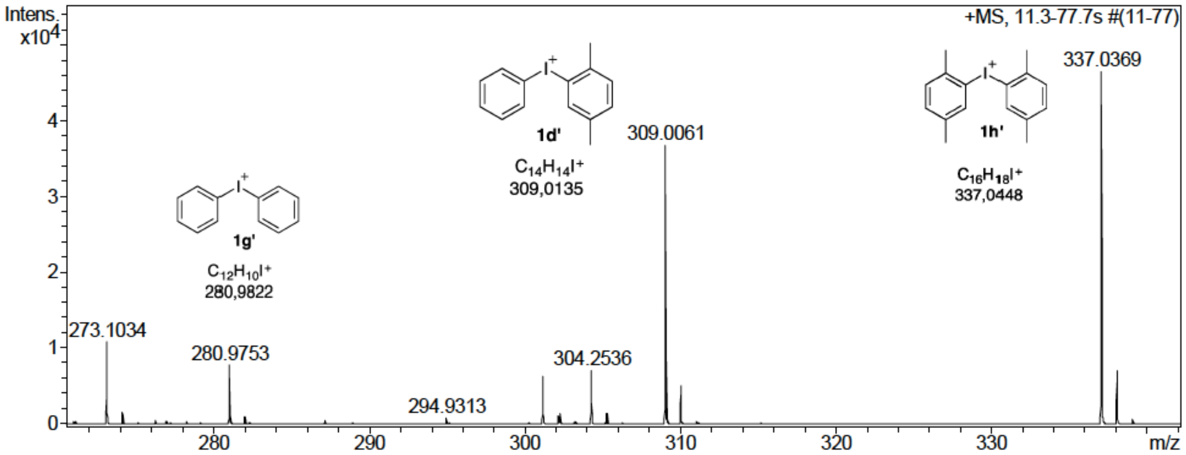

Figure S3. HRMS of aryl exchange after 5 min reaction of malonate **9** with salt **1d**.

Control experiments:
* Malonate **9** was arylated with di(*p*-tolyl)iodonium triflate (**1i**) in the presence of 2,6-dimethyliodobenzene (1 equiv). No aryl exchange was detected.
* Di(2,5-dimethylphenyl)iodonium triflate (**1h**) (1 equiv) and di(4-methoxyphenyl)iodonium triflate (**2h**) (1 equiv) were reacted with malonate **9** (1 equiv). The corresponding unsymmetric 2,5-dimethylphenyl-(4-methoxyphenyl)iodonium cation **2i'** was detected (Figure S4).

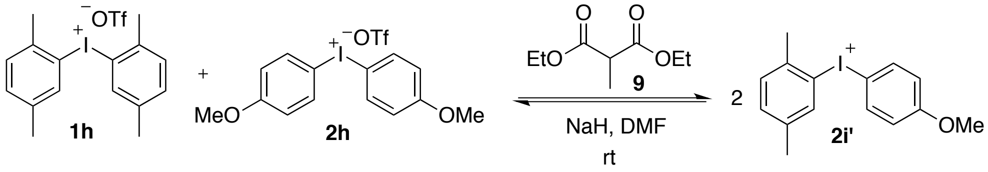

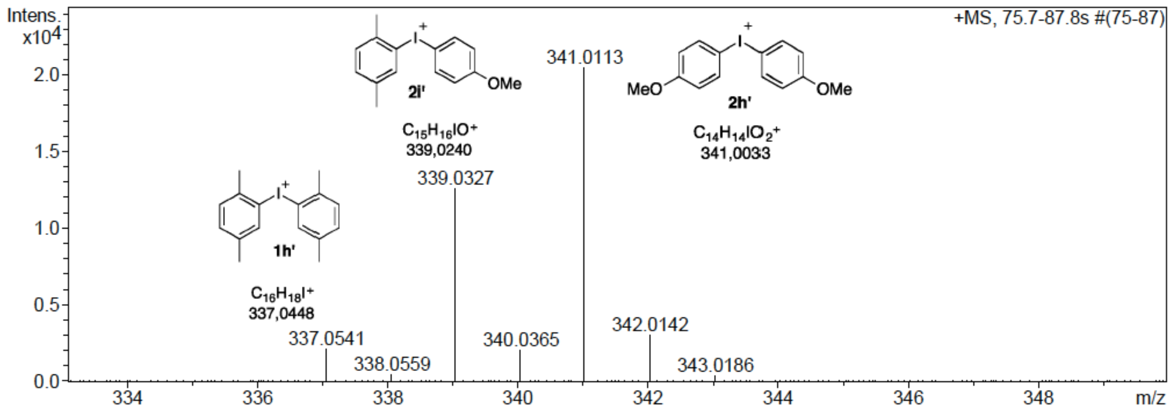

Figure S4. HRMS of aryl exchange after 5 min reaction of malonate **9** with salts **1h** and **2h**.

2.2 Aryl exchange study by NMR

An NMR study of the arylation of **9** with salt **2d** was conducted in a similar manner as the HRMS study. The reaction was performed as described in the paper (or Section 7). NMR samples were taken after 15, 25, 70, 120 and 180 minutes respectively. Each sample was taken *via* syringe (0.1 mL) and placed in a vial containing Et_2O and H_2O (1 mL, 1:1). The organic phase was evaporated *in vacuo* and dissolved in $CDCl_3$. The product ratios (Ph: Ar) throughout the reaction were:

15 min: 2.0 : 1; 25 min: 1.9 : 1; 70 min: 1.9 : 1; 120 min: 2.0 : 1; 180 min: 2.0 : 1.

2.3 Radical trap experiments

The radical trap experiments were performed by setting up reactions according to the experimental procedure in Sections **5-7** with the addition of a radical trap, which was added as the last reagent into the reactions. The results are given in Table S1.

We have previously reported radical trap experiments with phenols and DPE.[2]

Table S1. Radical trap experiments.

**Nucleophile**	**Salt**	**Radical trap (amount)**	**Yield[a] (%)**	**Yield without radical trap (%)**
m-Anisidine **6**	**1a**	DPE[b] (1 equiv)	28	53
m-Anisidine **6**	**2b**	DPE (1 equiv)	34	62
m-Anisidine **6**	**2c**	DPE (10 mol%)	20	50
m-Anisidine **6**	**2c**	TEMPO[c] (10 mol%)	10	50
Diethylmethyl malonate **9**	**1a**	DPE (1 equiv)	54	54

[a] Chemoselectivities were not altered. [b] 1,1-Diphenylethylene. [c] 2,2,6,6-Tetramethylpiperidin-1-oxyl.

3 Synthesis of Diaryliodonium Salts 1-2

3.1 One-pot methods

Many diaryliodonium salts are now commercially available. For convenience, we have synthesized most of the salts used in this investigation according to the one-pot methods previously developed in our group (Scheme S1).

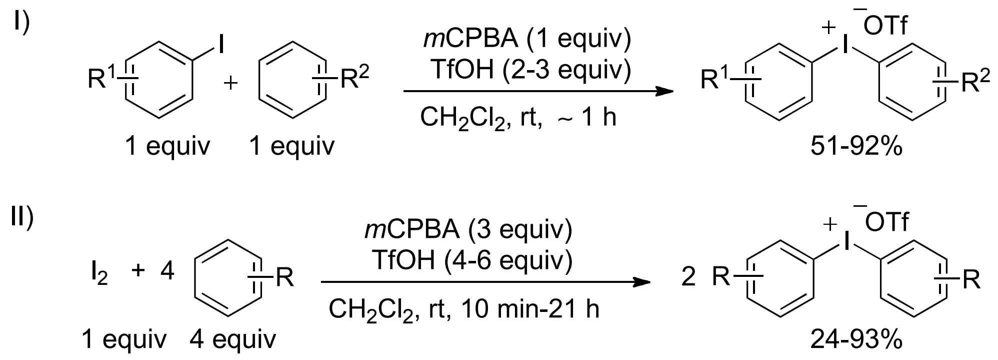

M. Bielawski, B. Olofsson, *Chem. Commun.* **2007**, 2521;
M. Bielawski, M. Zhu, B. Olofsson, *Adv. Synth. Catal.* **2007**, *349*, 2610;
M. Bielawski, B. Olofsson, *Org. Synth.* **2009**, *86*, 308-314.

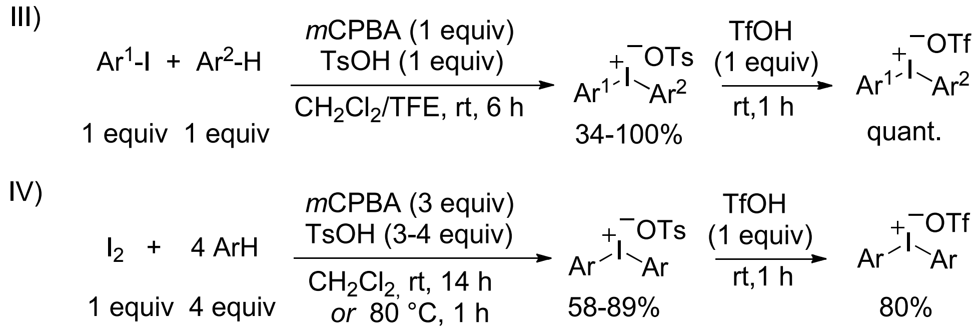

M. Zhu, N. Jalalian, B. Olofsson, *Synlett* **2008**, 592.

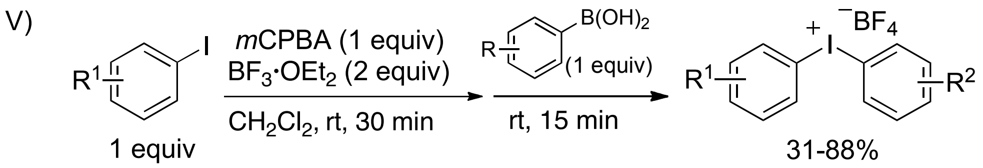

M. Bielawski, D. Aili, B. Olofsson, *J. Org. Chem.* **2008**, *73*, 4602.

Scheme S1. Our one-pot syntheses of diaryliodonium salts.

Table S2. Synthesis of diaryliodonium salts **1a-1f, 1h-j** and **2a**.

Entry	Salt	Method[a]	Acid (equiv)	T (°C)	Time	Yield (%)	Ref[b]
1	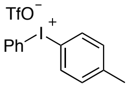 **1a**	I	2	0→rt	25 min	90	[3]
2	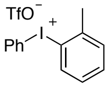 **1b**	I	2	rt	30 min	52	[3]
3	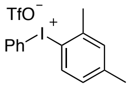 **1c**	I	2	0	2 h	95	-
4	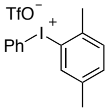 **1d**	I	2	rt	5 h	69	[3]
5	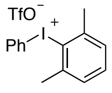 **1e**	V	2.5	rt	30 min + 30 min	76	-
6	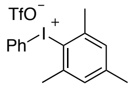 **1f**	I	2	rt	1 h	79	[3]
7[c]	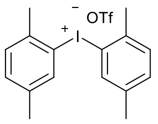 **1h**	II	4	rt	24 h	40	[3]
8	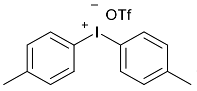 **1i**	III	3	0→rt	2 h	60	[3]
9[c]	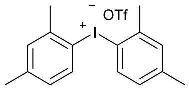 **1j**	II	4	rt	3 h	97	[4]
10	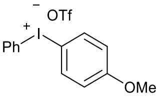 **2a**	I	2	-78→rt	10 + 10 min	87	[5]
		III	1	rt	6 h	100	

[a] Method in Scheme S1. [b] Reference to analytical data. [c] This salt was synthesized in order to selectively prepare the minor arylation product, to confirm the product ratio analysis.

2,4-Dimethyl(phenyl)iodonium triflate (1c):

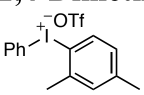

Isolated as a light grey solid; mp 128-129 °C, ^{1}H NMR (400 MHz, DMSO-d$_6$) δ 8.25 (d, *J* = 8.0, 1H), 8.17 (appd, *J* = 8.0, 2H), 7.65 (t, *J* = 4.0, 2H), 7.51 (t, *J* = 8.0, 2H), 7.38 (s, 1H), 7.13 (d, *J* = 8.0, 1H), 2.56 (s, 3H), 2.31 (s, 3H); ^{13}C NMR (100 MHz, DMSO-d$_6$) δ 143.3, 140.4, 137.0, 134.9, 132.0, 131.9, 131.8, 131.8, 129.9, 117.7, 115.9, 24.8, 20.7. HRMS (ESI) *m*/*z* calculated for $C_{14}H_{14}I$ ([M−OTf]$^+$) 309.0094, found 309.0080.

2,6-Dimethyl(phenyl)iodonium triflate (1e):

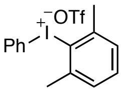

Isolated as a light grey solid; mp 137-138 °C, ^{1}H NMR (400 MHz, DMSO-d$_6$) δ 8.01 (appd, *J* = 8.0, 2H), 7.65 (t, *J* = 8.0, 1H), 7.55-7.46 (m, 3H), 7.40 (appd, *J* = 8.0, 2H), 2.65 (s, 6H); ^{13}C NMR (100 MHz, DMSO-d$_6$) δ 141.7, 134.6, 131.9, 131.9, 129.1, 126.2, 114.4, 26.5. HRMS (ESI) *m*/*z* calculated for $C_{14}H_{14}I$ ([M−OTf]$^+$) 309.0149, found 309.0135.

3.2 Stepwise methods

General procedure for anion exchange to triflate

The diaryliodonium salt (5 mmol) was dissolved in dichloromethane (30 mL) and washed with an aqueous NaOTf solution (3 x 50 mmol). The organic layer was concentrated without drying. Et_2O (20 mL) was added and the mixture was stirred at room temperature for 30 min to precipitate a solid. The solid was filtrated and washed with Et_2O and dried under vacuum to give the triflate salt. The anion exchange was confirmed by NMR analysis.

2-Methoxyphenyl(phenyl)iodonium triflate (2b):

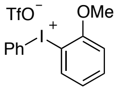

Synthesized by a known method,[6] followed by anion exchange with NaOTf. Isolated as a light yellow solid; mp 148-150 °C; ^{1}H NMR (400 MHz, DMSO-d$_6$) δ 8.30 (dd, *J* = 7.9, 1.4, 1H), 8.20-8.10 (m, 2H), 7.70-7.61 (m, 2H), 7.54-7.47 (m, 2H), 7.31 (dd, *J* = 1.3, 8.4, 1H), 7.09 (td, *J* = 7.9, 1.4, 1H), 3.94 (s, 3H); ^{13}C NMR (100 MHz, DMSO-d$_6$) δ 156.4, 137.2, 135.1, 134.9, 131.9, 131.6, 123.4, 120.7 (q, $^1J_{C-F}$ = 322), 115.8, 113.1, 106.5, 57.1; HRMS (ESI) *m*/*z* calculated for $C_{13}H_{12}IO$ ([M−OTf]$^+$) 310.9927, found 310.9912.

2,4-Dimethoxyphenyl(phenyl)iodonium triflate (2c):

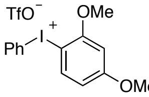

Synthesized by a known method,[7] followed by anion exchange with NaOTf. Isolated as a light yellow solid; mp 92-94 °C; ^{1}H NMR (400 MHz, DMSO-d$_6$) δ 8.18 (d, *J* = 8.8, 1H), 8.09- 8.03 (m, 2H), 7.66-7.59 (m, 1H), 7.53- 7.45 (m, 2H), 6.80 (d, *J* = 2.6, 1H), 6.69 (dd, *J* = 2.6, 8.8, 1H), 3.93 (s, 3H), 3.83 (s, 3H); ^{13}C NMR (100 MHz, DMSO-d$_6$) δ 164.7, 158.2, 138.3, 134.6, 131.7, 131.5, 122.3, 120.7 (q, $^1J_{C-F}$ = 322), 119.1, 116.3, 108.9, 99.7, 95.8, 57.3, 56.0; HRMS (ESI) *m*/*z* calculated for $C_{14}H_{14}IO_2$ ([M−OTf]$^+$) 314.0033, found 314.0030.

2,5-Dimethoxyphenyl(phenyl)iodonium triflate (2d):

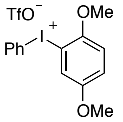

Synthesized by a known method,[6] followed by anion exchange with NaOTf. Isolated as a light yellow solid; mp 168-170 °C; ^{1}H NMR (400 MHz, DMSO-d$_6$) δ 8.12 (d, *J* = 8.0, 2H), 7.96 (brs, 1H), 7.65 (appt, 1H), 7.51 (appt, 2H), 7.29-7.18 (m, 2H), 3.86 (s, 3H), 3.77 (s, 3H); ^{13}C NMR (100 MHz, DMSO-d$_6$) δ 154.2, 150.7, 135.0, 131.9, 131.6, 122.1, 120.7 (q, $^1J_{C-F}$ = 322), 119.9, 115.9, 113.5, 106.3, 57.4, 56.2; HRMS (ESI) *m*/*z* calculated for $C_{14}H_{14}IO_2$ ([M−OTf]$^+$) 314.0033, found 314.0030.

2,6-Dimethoxyphenyl(phenyl)iodonium triflate (2e):

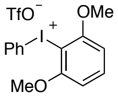

Synthesized by a known method,[6] followed by anion exchange with NaOTf. Isolated as a light yellow solid; mp 152-153 °C; ^{1}H NMR (400 MHz, DMSO-d$_6$) δ 7.95 (dd, J = 1.0, 8.3, 1H), 7.62-7.54 (m, 1H), 7.48-7-41 (m, 1H), 6.87 (t, J = 8.4, 1H), 3.94 (s, 3H); ^{13}C NMR (100 MHz, DMSO-d$_6$) δ 158.1, 135.7, 134.6, 131.4, 131.4, 116.8, 105.3, 99.1, 57.3; HRMS (ESI) m/z calculated for $C_{14}H_{14}IO_2$ ([M−OTf]$^+$) 314.0033, found 314.0023.

2,4,6-Trimethoxyphenyl(phenyl)iodonium triflate (2f):

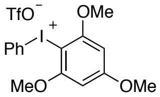

Synthesized by a known method,[7] followed anion exchange with NaOTf. Isolated as a white solid in 94% yield; mp 114-116 °C; ^{1}H NMR (400 MHz, DMSO-d$_6$) δ ^{1}H NMR (500 MHz, DMSO-d$_6$) δ 7.92 (d, J = 7.4, 2H), 7.61 (t, J = 7.4, 1H), 7.47 (t, J = 7.8, 2H), 6.46 (s, 2H), 3.94 (s, 6H), 3.86 (s, 3H); ^{13}C NMR (100 MHz, DMSO-d$_6$) δ 166.2, 159.4, 134.4, 131.6, 128.1, 125.5, 122.31 120.7 (q, $^1J_{C\text{-}F}$ = 322), 119.1, 116.1, 92.1, 87.0, 57.4, 56.2; HRMS (ESI) m/z calculated for $C_{15}H_{16}IO_2$ ([M−OTf]$^+$) 314.0033, found 314.0023.

4 Arylation of Phenol 3 to Products 4, 5

Experimental procedure:[8]

To a suspension of ^{t}BuOK (1.1 equiv, 43 mg, 0.37 mmol) in THF (1.5 mL) was added phenol **3** (1.0 equiv, 0.34 mmol) at 0 °C and the reaction was left to stir at this temperature for 15 min. Diaryliodonium salt **1** or **2** (1.2 equiv, 0.40 mmol) was added in one portion and the reaction was stirred in an oil bath preheated to 40 °C until TLC indicated complete consumption of **1** or **2.** The reaction was then quenched with H_2O at 0 °C, the organic phase was separated and the water phase was extracted with CH_2Cl_2 (3 × 10 mL). The combined organic phases were dried (Na_2SO_4) and concentrated *in vacuo*. The crude material was purified by flash chromatography to give the diaryl ethers **4** and **5**. The product ratio was determined by isolating **4** and **5** respectively.

The analytical data of **4**[8], **5a**[9] and **5b-f**[4] were in agreement with previous reports.

5 Arylation of Aniline 6 to Products 7, 8

5.1 Arylation with unsymmetric diaryliodonium salts 1, 2

Experimental procedure:[10]
Diaryliodonium salt **1** or **2** (1 equiv, 0.25 mmol) was dissolved in dry DMF (2 mL). *m*-Anisidine (1 equiv, 0.028 mL, 0.25 mmol) was added under stirring at rt. The reaction mixture was submitted to a 130 °C oilbath and stirred for 24 h. The reaction was treated with Na_2CO_3 (1 M, 2 mL), extracted with EtOAc (3 × 5 mL) and washed with H_2O (1 × 10 mL) and brine (2 × 10 mL). The combined organic phases were dried ($MgSO_4$) and concentrated *in vacuo*. The crude material was purified with flash chromatography to give the diarylamines **7** and **8** as an inseparable mixture. The product ratio was determined by NMR from the crude mixture.

***N*-(3-methoxyphenyl)-aniline (7):**

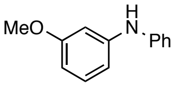

Synthesized according to the general protocol with salt **2e** to give **7** in 70% yield as a colorless oil. Analytical data were in accordance with previously reported data.[11]

***N*-(3-methoxyphenyl)-2-methylaniline (8b):**

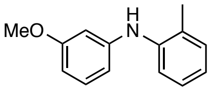

Synthesized according to the general procedure using 2-methylphenyl(phenyl)iodonium triflate (**1b**) to give **8b** in 15% yield as a colorless oil. Analytical data were in accordance with previously reported data.[12]

***N*-(3-methoxyphenyl)-2,4,6-trimethylaniline (8f):**

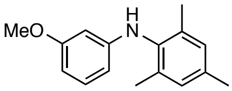

Synthesized according to the general procedure using salt **1f**. Isolated as a mixture of **7** and **8f** (15:1, 50% yield). The presence of **8f** was confirmed by comparing with the reported analytical data.[13]

***N*-(3-methoxyphenyl)-2,5-dimethoxyaniline** (**8i**):

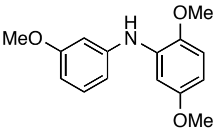

Synthesized according to the general procedure using 2,5-dimethoxyphenyl(phenyl)iodonium triflate (**2d**) to give **7** and **8i** (2.3:1, 30% combined yield). **8i** was isolated by column chromatography (Pentane → pentane : EtOAc 12:1) in 9% yield as a colorless oil. ^{1}H NMR (400 MHz, $CDCl_3$) δ 7.26 (d, *J* = 8.64, 1H), 7.15 (t, *J* = 8.12, 1H), 6.63-6.57 (m, 2H), 6.55 (d, *J* = 2.68, 1H), 6.47 (dd, *J* = 8.64, 2.68, 1H), 6.43 (appd, 1H), 3.86 (s, 3H), 3.83 (s, 3H), 3.79 (s, 3H) ^{13}C NMR (100 MHz, $CDCl_3$) δ 160.9, 155.5, 151.5, 146.3, 130.2, 125.6, 119.9, 109.3, 105.3, 104.1, 102.2, 99.7, 55.9, 55.4, 31.1; HRMS (ESI) *m*/*z* calculated for $C_{15}H_{18}NO_3$ ($[M+H]^+$) 259.1281 found 259.1239.

5.2 Arylations to obtain reference products

The following compounds were synthesized separately to obtain NMR data of the pure minor products, in order to confirm the NMR analysis of the crude mixtures.

***N*-(3-methoxyphenyl)-4-methylaniline (8a):**

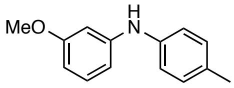

Synthesized according to the general protocol using di(4-methylphenyl)iodonium triflate (**1i**) to give **8a** in 66% yield as a colorless oil. Analytical data were in agreement with previously reported data.[14]

***N*-(3-methoxyphenyl)-2,4-dimethylaniline (8c):**

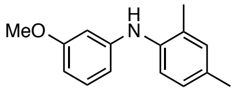

Synthesized according to the general protocol using di(2,4-dimethylphenyl)iodonium triflate (**1j**) to give **8c** in 19% yield as a colorless oil. ^{1}H NMR (400 MHz, $CDCl_3$) δ 7.19-7.10 (m, 2H), 7.05 (br s, 1H), 7.01-6.95 (app d, J = 8.39, 1H), 6.44-6.39 (m, 2H), 3.77 (s, 3H), 2.32 (s, 3H), 2.23 (s, 3H); ^{13}C NMR (100 MHz, $CDCl_3$) δ 161.0, 146.7, 138.1, 132.9, 131.9, 130.5, 130.2, 128.8, 127.5, 121.8, 109.1, 105.1, 102.1, 55.4, 20.9, 18.0; HRMS (ESI) *m*/*z* calculated for $C_{15}H_{18}NO$ ([M + H]$^+$) 228.1383 found 228.1383.

***N*-(3-methoxyphenyl)-2,5-dimethylaniline (8d):**

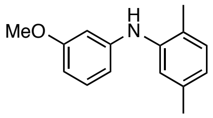

Synthesized according to the general protocol using di(2,5-dimethylphenyl)iodonium triflate (**1h**) to give **8d** in 30% yield as a colorless oil. Analytical data were in agreement with previously reported data.[15]

***N*-(3-methoxyphenyl)-2,6-dimethylaniline (8e):**[16]

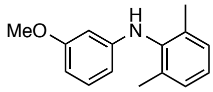

t-Bu_3PBF_4 (0.005 mmol, 1.5 mg), $Pd(OAc)_2$ (0.003 mmol, 0.7 mg) and Cs_2CO_3 (3.05 mmol, 0.994 g) was added to a vial. Inert atmosphere was introduced by applying vacuum and then backfilling with argon. A mixture of *m*-anisidine (0.5 mmol, 0.057 mL) and 2,6-dimethylbromobenzene (3 mmol, 0.400 mL) in dry DMF (2 mL) was added to the solid compounds. The reaction vial was capped and submitted to μ-wave irradiation at 140 ^{0}C for 1 h. The reaction mixture was filtered through a plug of SiO_2 (5 g). The SiO_2 was washed with CH_2Cl_2 (10 mL). Column chromatography (pentane → pentane : EtOAc 9 : 1) gave **8e** in 66% yield as a colorless oil. ^{1}H NMR (400 MHz, $CDCl_3$) δ 7.14-7.05 (m, 4H), 6.33 (d, J = 8.20, 1H), 6.16 (d, J = 8.04, 1H), 6.05 (t, J = 2.28, 1H), 3.74 (s, 3H), 2.23 (s, 1H); ^{13}C NMR (100 MHz, $CDCl_3$) δ 161.1, 148.0, 138.3, 136.3, 130.2, 128.7, 126.1, 106.9, 103.4, 99.8, 55.3, 18.5; HRMS (ESI) *m*/*z* calculated for $C_{14}H_{18}NO^+$ ([M+H]$^+$) 228.1383 found 228.1360.

N-(3-methoxyphenyl)-4-methoxyaniline (8g):

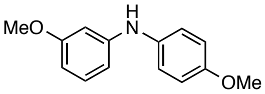

Synthesized according to the same procedure as **8e** using 2-methoxybromobenzene with 1.5 h reaction time. Column chromatography (pentane → pentane : EtOAc 9 : 1) gave **8g** in 50% yield as a colorless oil. Analytical data were in agreement with previously reported data.[17]

N-(3-methoxyphenyl)-2-methoxyaniline (8h):

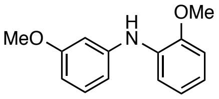

Synthesized according to the same procedure as **8e** with 1.5 h reaction time. Column chromatography (pentane → pentane : EtOAc 9 : 1) gave **8h** in 52% yield as a colorless oil. Analytical data were in agreement with previously reported data.[18]

6 Arylation of Malonate 9 to Products 10, 11

6.1 Arylation with unsymmetric diaryliodonium salts 1, 2

Experimental procedure:[19]
NaH (60% dispersed in mineral oil, 1.3 equiv, 0.33 mmol, 10 mg) was suspended in DMF (0.5 mL) and diethylmethylmalonate **9** (0.25 mmol, 44 mg) was added dropwise at 0 °C. The reaction was allowed to stir at rt for 10 min. A solution of diaryliodonium salt **1** or **2** (1.3 equiv, 0.33 mmol) in DMF (0.5 mL) was added *via* cannulation to the reaction mixure at 0 °C. The reaction mixture was stirred at rt until TLC indicated complete consumption of **9**. The reaction was quenched with H_2O at 0 °C, extracted with EtOAc (3 × 5 mL) and washed with H_2O (1 × 10 mL) and brine (2 × 10 mL). The combined organic phases were dried ($MgSO_4$) and concentrated *in vacuo*. The crude material was purified with flash chromatography to give products **10** and **11** as an inseparable mixture. The product ratio was determined by NMR from the crude mixture.

Diethyl 2-methyl-2-phenylmalonate (10):

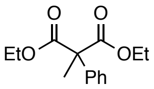

Synthesized according to the general procedure using salt **1f**, giving **10** as a colorless oil in 55% yield. Analytical data was in agreement with the reported analytical data.[20]

Diethyl 2-(2-methylphenyl)-2-methylmalonate (11b):

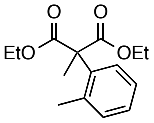

Synthesized according to the general procedure using salt **1b**, and isolated as a mixture of **10** and **11b**. The presence of **11b** was confirmed by comparing with the reported analytical data.[21]

6.2 Arylations to obtain reference products

The following compounds were synthesized separately to obtain NMR data of the pure minor products, in order to confirm the NMR analysis of the crude mixtures.

Diethyl 2-(4-methylphenyl)-2-methylmalonate (11a):

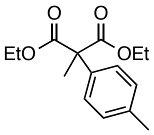

Synthesized according to the general protocol using di(4-methylphenyl)iodonium triflate (**1i**) to give **11a** in 53% yield as a colorless oil. Analytical data were in agreement with previous report.[22]

Diethyl 2-(2,4-dimethylphenyl)-2-methylmalonate (11c):

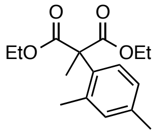

Synthesized according to the general protocol using di(2,4-dimethylphenyl)iodonium triflate (**1j**) to give **11c** in 30% yield as a colorless oil. ^{1}H NMR (400 MHz, $CDCl_3$) δ7.05-6.94 (m, 3H), 4.35-4.15 (m, 4H), 2.30 (s, 3H), 2.25 (s, 3H), 1.86 (s, 3H), 1.27 (t, J = 7.2, 6H); ^{13}C NMR (100 MHz, $CDCl_3$) δ 172.0, 137.3, 136.8, 135.2, 133.2, 126.9, 126.7, 61.9, 59.4, 23.2, 21.0, 20.9, 14.1; HRMS (ESI) *m/z* calculated for $C_{16}H_{12}NaO_4$ ($[M + Na]^+$) 301.1410 found 301.1406.

Diethyl 2-(2,5-dimethylphenyl)-2-methylmalonate (11d):

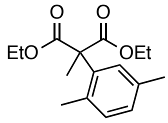

Synthesized according to the general procedure using di(2,5-dimethylphenyl)iodonium triflate (**1h**) to give **11d** in 41% yield as a colorless oil. Analytical data were in agreement with previous report.[21]

Diethyl 2-(4-methoxyphenyl)-2-methylmalonate (11g):

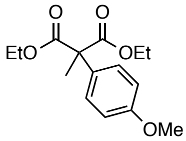

Synthesized in a two-step procedure from diethylmalonate.[23] CuI (0.01 g, 0.05 mmol), 2-picolinic acid (0.012 g, 0.1 mmol) and Cs_2CO_3 (0.977 g, 3 mmol) was added to a dry round-bottomed flask containing a magnetic stirrer bar, submitted to vacuum for 15 min and then backfilled with argon. Dry 1,4-dioxane (2 mL) was added *via* syringe followed by diethylmalonate (0.304 mL, 2 mmol) and 4-iodoanisole (0.234 g, 1 mmol). The reaction was stirred at rt under argon for 24 h. Column chromatography (pentane → pentane : EtOAc 5 : 1) afforded the product in 91% yield.
Diethyl 2-(2-methoxyphenyl)methylmalonate (0.242 g, 0.91 mmol) was dissolved in dry DMF (10 mL) in a round-bottomed flask. NaH (60% dispersed in mineral oil, 0.028 g, 1.18 mmol) was added at 0 °C and the mixture was allowed to stir at that temperature for 15 min. MeI (0.57 mL, 0.92) was added at 0 °C and the reaction was allowed to reach rt and was stirred for 18 h. The reaction was then cooled to 0 °C and water (2 mL) was added. After 15 min, the reaction mixture was transferred to a separatory funnel and extracted with EtOAc (3 x 40 mL). The combined organic layers where washed with water (2 x 60 mL) and brine (2 x 60 mL). Column chromatography (pentane → pentane : ethylacetate 5 : 1) afforded **11g** in 30% yield as a yellow oil. Analytical data were in agreement with previous report.[24]

Diethyl 2-(2-methoxyphenyl)-2-methylmalonate (11h):

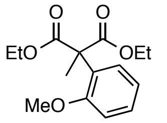

Synthesized in a two-step procedure from diethylmalonate[23] as described for **11g**, using 2-bromoanisole and 24 h reaction time. The crude mixture was purified by column chromatography (pentane → pentane : ethylacetate 9 : 1) to give **11h** in 79% yield as a yellow

oil. ^{1}H NMR (400 MHz, $CDCl_3$) δ 7.31-7.25 (m, 1H), 7.11 (dd, J = 7.72, 1.64, 1H), 6.97-6.88 (m, 2H), 4.31-4.17 (m, 4H), 3.79 (s, 3H), 1.81 (s, 3H), 1.26 (t, J = 7.08, 6 H); ^{13}C NMR (100 MHz, $CDCl_3$) δ 171.7, 157.1, 129.4, 128.9, 127.2, 120.9, 111.8, 61.7, 57.9, 55.6, 22.0, 14.2. HRMS (ESI) m/z calculated for $C_{15}H_{20}NaO_5$ ($[M + Na]^+$) 303.1203 found 303.1192.

Diethyl 2-(2,4-dimethoxyphenyl)-2-methylmalonate (11i):

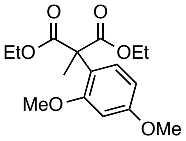

Prepared according to the general procedure using the symmetrical bis(2,4-dimethylphenyl)iodonium triflate **12b** to give **11i** in 57% yield as a colorless oil. ^{1}H NMR (400 MHz, $CDCl_3$) δ 7.04-6.94 (m, 3H), 4.33-4.17 (m, 4H), 2.30 (s, 3H), 2.25 (s, 3H), 1.27 (t, J = 7.08, 6H); ^{13}C NMR (100 MHz, $CDCl_3$) δ 171.9, 160.4, 158.1, 127.6, 121.9, 104.1, 99.8, 61.6, 57.2, 55.6, 55.5, 22.1, 14.2; HRMS (ESI) m/z calculated for $C_{16}H_{22}NaO_6$ ($[M + Na]^+$) 333.1309 found 333.1325.

Analysis references

[1] A. I. Vogel, B. S. Furniss, A. J. Hannaford, V. Rogers, P. W. G. Smith, A. R. Tatchell, *Vogel's Textbook of Practical Organic Chemistry*, **1978**.

[2] N. Jalalian, T. B. Petersen, B. Olofsson, *Chem. Eur. J.* **2012**, *18*, 14140-14149.

[3] M. Bielawski, M. Zhu, B. Olofsson, *Adv. Synth. Catal.* **2007**, *349*, 2610-2618.

[4] N. Jalalian, T. B. Petersen, B. Olofsson, *Chem. Eur. J.* **2012**, *18*, 14140-14149.

[5] M. Zhu, N. Jalalian, B. Olofsson, *Synlett* **2008**, 592-596.

[6] S. L. J.-H. Chun, Y.-S. Lee, V. W. Pike, *J. Org. Chem* **2010**, *75*, 3332-3338.

[7] G. F. Koser, R. H. Wettach, *J. Org. Chem.* **1980**, *45*, 1542-1543.

[8] N. Jalalian, E. E. Ishikawa, L. F. Silva, B. Olofsson, *Org. Lett.* **2011**, *13*, 1552-1555.

[9] A. B. Naidu, E. A. Jaseer, G. Sekar, *J. Org. Chem.* **2009**, *74*, 3675-3679.

[10] M. A. Carroll, R. A. Wood, *Tetrahedron* **2007**, *63*, 11349-11354.

[11] L. Ackermann, R. Sandmann, W. Song, *Org. Lett.* **2011**, *13*, 1784-1786.

[12] T. Ogata, J. F. Hartwig, *J. Am. Chem. Soc.* **2008**, *130*, 13848-13849.

[13] D. H. R. Barton, D. M. X. Donnelly, J. P. Finet, P. J. Guiry, *J. Chem. Soc., Perkin Trans. 1* **1991**, 2095-2102.

[14] A. Hajra, Y. Wei, N. Yoshikai, *Org. Lett.* **2012**, *14*, 5488-5491.

[15] R. J. Hall, J. Marchant, A. M. F. Oliveira-Campos, M. J. R. P. Queiroz, P. V. R. Shannon, *J. Chem. Soc., Perkin Trans. 1* **1992**, 3439-3450.

[16] O. Verho, E. V. Johnston, E. Karlsson, J.-E. Bäckvall, *Chem. Eur. J.* **2011**, *17*, 11216.

[17] Q. Shen, T. Ogata, J. F. Hartwig, *J. Am. Chem. Soc.* **2008**, *130*, 6586-6596.

[18] C. V. Reddy, J. V. Kingston, J. G. Verkade, *J. Org. Chem.* **2008**, *73*, 3047-3062.

[19] C. H. Oh, J. S. Kim, H. H. Jung, *J. Org. Chem.* **1999**, *64*, 1338-1340.

[20] C. E. Katz, J. Aube, *J. Am. Chem. Soc.* **2003**, *125*, 13948-13949.

[21] S. Ghosh, S. N. Pardo, R. G. Salomon, *J. Org. Chem.* **1982**, *47*, 4692-4702.

[22] A. Piorko, A. Abd-El-Aziz, C. C. Lee, R. G. Sutherland, *J. Chem. Soc., Perkin Trans. 1* **1989**, 469-475.

[23] S. F. Yip, H. Y. Cheung, Z. Zhou, F. Y. Kwong, *Org. Lett.* **2007**, *9*, 3469-3472.

[24] R. P. Kopinski, J. T. Pinhey, B. A. Rowe, *Aust. J. Chem.* **1984**, *37*, 1245-1254.

7 Computational details

All calculations reported in the present study were carried out using density functional theory with the B3LYP functional,[25] as implemented in the Gaussian09 program package.[26] For geometry optimizations, the 6-31G(*d,p*) basis set was used for the C, N, O, F, Cl, H elements, and the LANL2DZ[27] pseudopotential with the corresponding basis set, augmented with *d* polarization and *p* diffuse functions,[28] for I. Based on these optimized geometries, single-point calculations were carried out with the same basis set for I and the 6-311+G(*2d,2p*) basis set for all other elements. The stationary points were confirmed as minima (no imaginary frequencies) or transition states (only one imaginary frequency) by analytical frequency calculations at the same theory level as the geometry optimizations. The reported energies are Gibbs free energies, which include zero-point vibrational corrections, thermal corrections at 298 K (or 403 K for reaction with anilines), and solvation free energies. The latter are calculated as single-point corrections on the optimized structures using the conductor-like polarizable continuum model (CPCM)[29] method with the UFF radii and with the parameters for THF or DMF, according to experiments. All energies are also corrected for dispersion effects using the B3LYP-D3 method of Grimme,[30] with Becke and Johnson (BJ) damping.[31]

7.1 Illustrative Figures

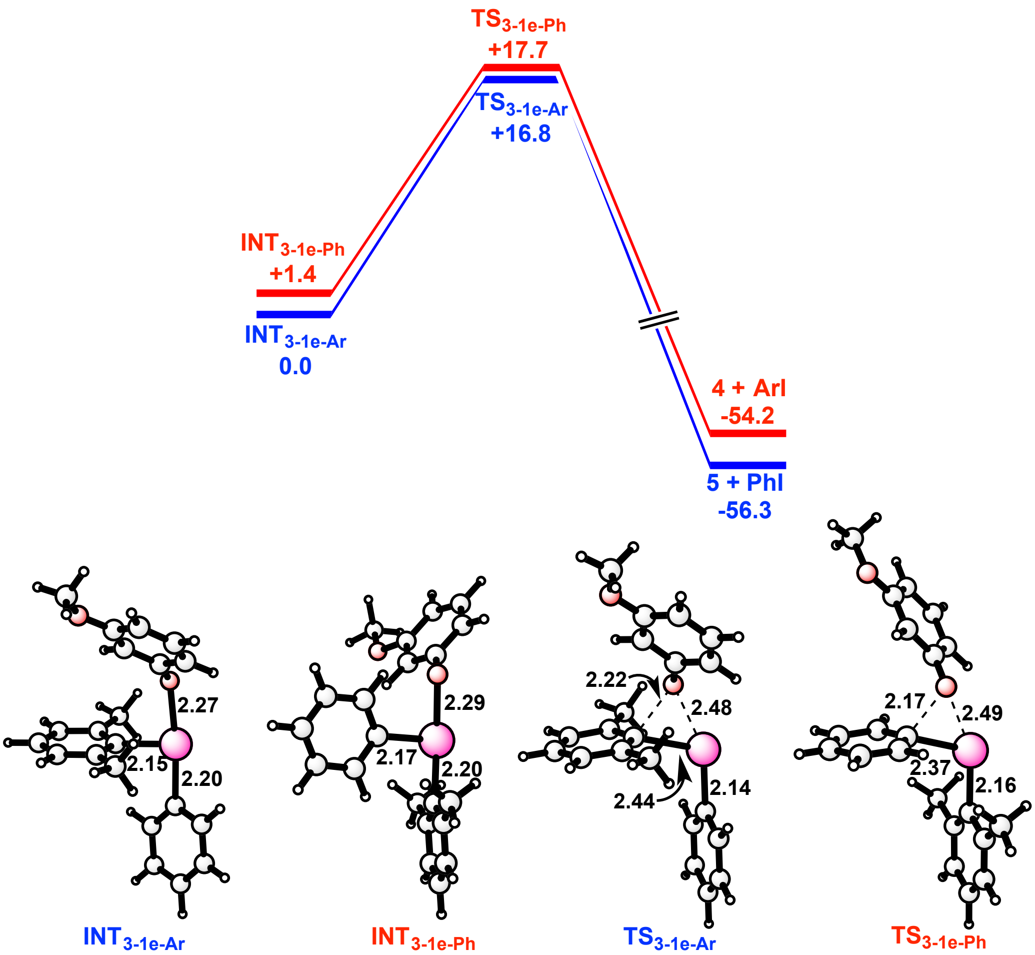

Figure S5: Free energy profile and optimized structures of starting complexes and transition states for the reaction of phenol **3** with **1e**. Distances are in angstroms and energies are in kcal/mol. This figure is given in black and white in the paper.

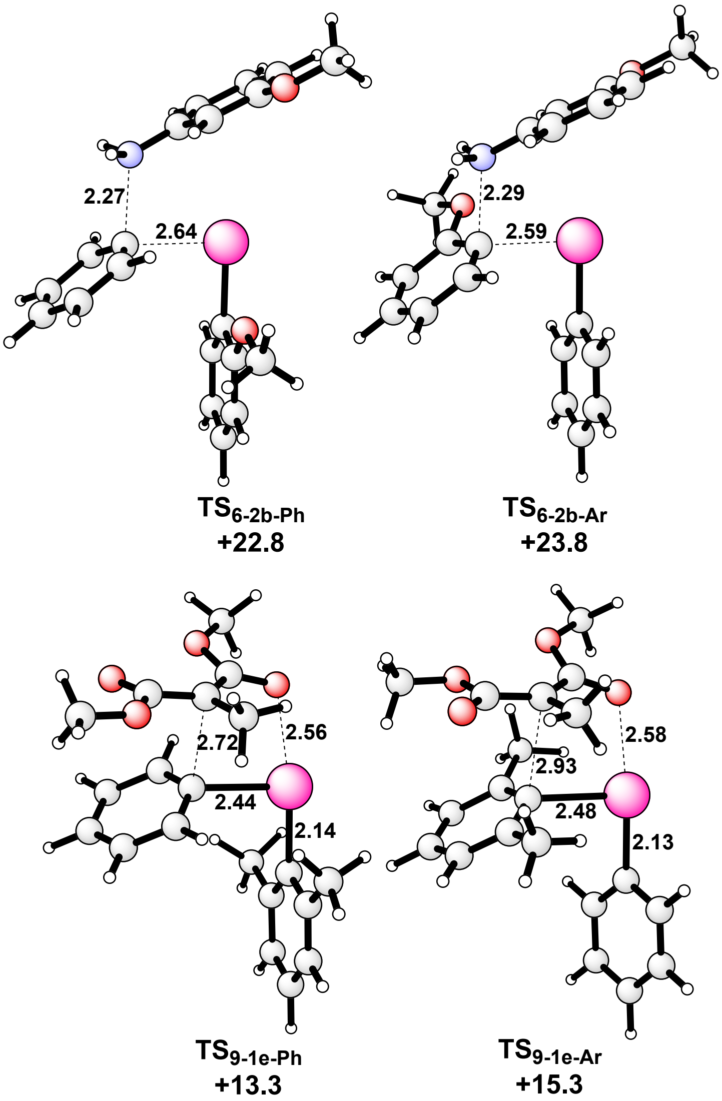

Figure S6. Optimized TS structures of aniline **6** with salt **2b**, and malonate **9** with salt **1e**. Distances are in angstroms and energies are in kcal/mol. This figure is given in black and white in the paper.

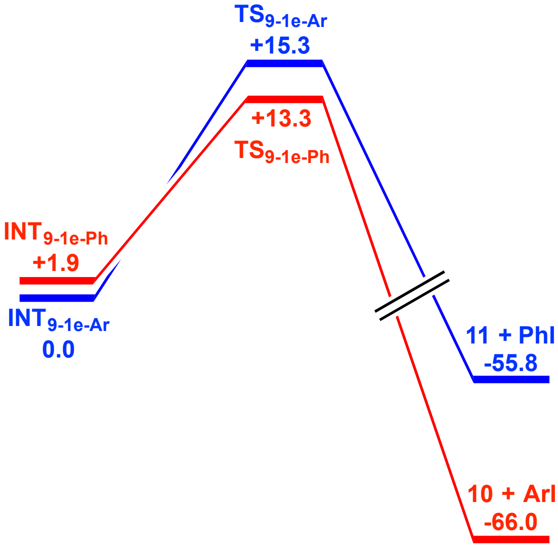

Figure S7: Free energy profile for the reaction of malonate **9** with **1e**. Energies are given in kcal/mol. TS structures are given in Figure S6.

7.2 Correlation plots between absolute barriers and Hammett σ for the ligand coupling between phenoxide and aryl(phenyl)iodonium salts with one *para*-substituted aryl group.

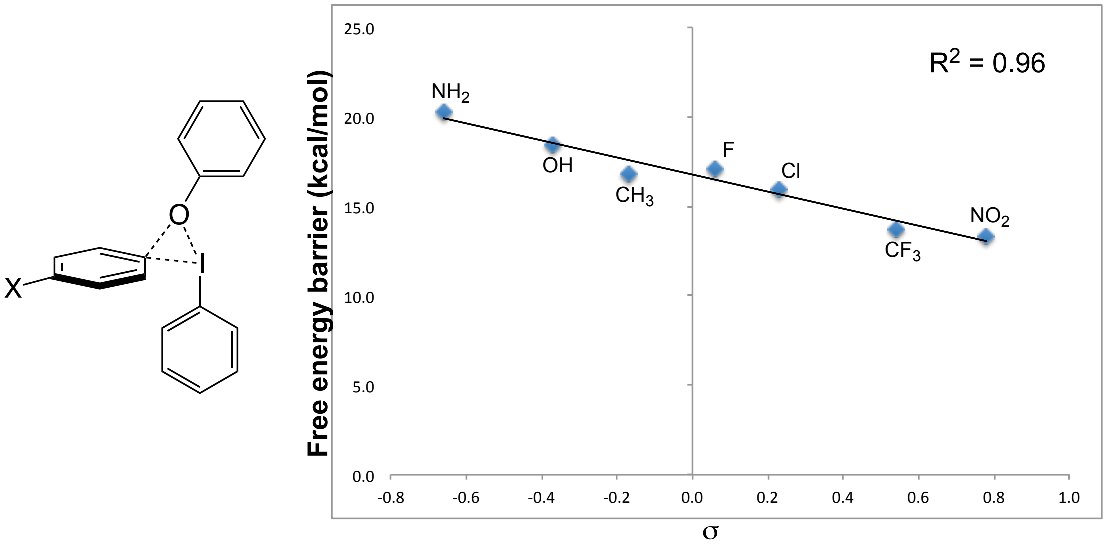

Figure S8. *Substituted aryl transfer.*

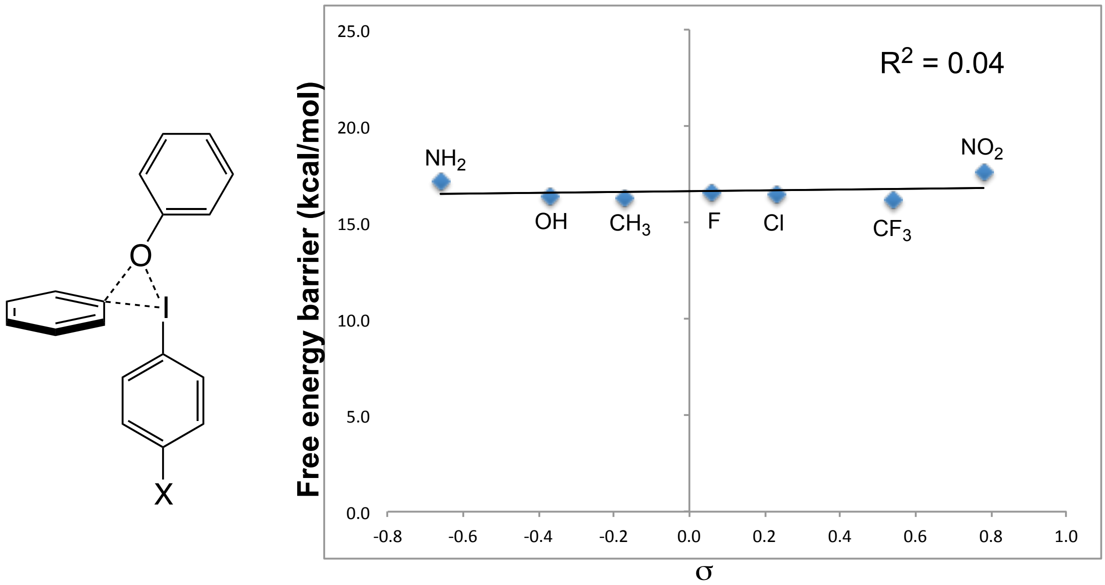

Figure S9. *Phenyl transfer.*

7.3 Optimized structures and Cartesian coordinates of stationary points

- $INT_{3\text{-}1a}$

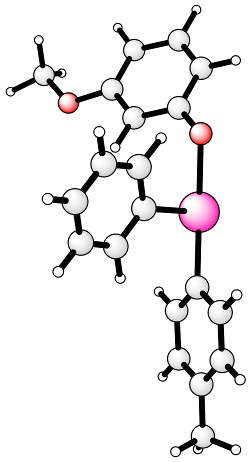

B3LYP/BS2 energy: -935.576772626 a.u.
ZPE: 0.33432 a.u.
Thermal correction to Gibbs Free Energy: 0.275986 a.u.
Solvation energy: -7.60 kcal/mol
Dispersion correction: -60.65 kcal/mol

I	0.48046700	-0.43620000	-0.90243900
C	0.38877000	1.61676000	-0.19824300
C	-0.58395200	2.43561800	-0.76036900
C	-0.64899700	3.76463300	-0.33175900
C	0.23452700	4.24393300	0.63633200
C	1.19297600	3.39238400	1.18686700
C	1.28251800	2.06042500	0.77018500
H	-1.27363500	2.02659500	-1.49069700
H	-1.40033300	4.42086200	-0.76103500
H	0.17377100	5.27714700	0.96492400
H	1.87708900	3.75502000	1.94853900
H	2.02875800	1.40294900	1.19865600
C	2.50795100	-0.67461200	-0.13038800
C	2.71153200	-1.40212200	1.04635200
C	4.00991500	-1.66642400	1.48937700
C	5.12492200	-1.21428000	0.77257200
C	4.90470400	-0.49194400	-0.41003400
C	3.61312100	-0.22487500	-0.86275600
H	1.86351200	-1.76616100	1.62071800
H	4.15638400	-2.23497100	2.40459000
H	5.75647500	-0.13942900	-0.98708700
H	3.47181600	0.33262800	-1.78487600
C	6.52760300	-1.47847000	1.26578100
H	6.56150000	-2.34427500	1.93291600
H	7.21553000	-1.66182000	0.43472600
H	6.91725000	-0.61852800	1.82473800
C	-2.69359300	-0.46477100	-1.08676700
C	-3.74916300	-1.07662800	-1.80583200
C	-2.85556100	-0.27410300	0.29689000
C	-4.90796200	-1.46341200	-1.14773100
H	-3.62866600	-1.22414700	-2.87425100
C	-4.02833900	-0.67881800	0.94596600
H	-2.08244900	0.20543900	0.88775600
C	-5.07144600	-1.27754800	0.23187800
H	-5.71183200	-1.92902700	-1.71304400
H	-5.98518200	-1.59543200	0.71842700
O	-1.59815900	-0.07593300	-1.75577400
O	-4.05704400	-0.43655700	2.29790000
C	-5.22433800	-0.79755500	3.01081200
H	-5.04683500	-0.51397800	4.05040000
H	-6.11081800	-0.26547500	2.64001700
H	-5.41661600	-1.87801000	2.96204000

- TS_{isomer}

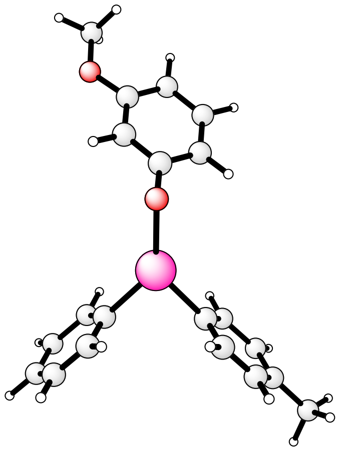

B3LYP/BS2 energy: -935.555786091 a.u.
ZPE: 0.332919 a.u.
Thermal correction to Gibbs Free Energy: 0.274296 a.u.
Solvation energy: -11.10 kcal/mol
Dispersion correction: -57.45 kcal/mol

I	0.36619700	0.18774500	-0.62698900
C	1.36530700	2.01376400	0.03430000
C	2.11086600	2.75549700	-0.88216600
C	2.70430800	3.94874100	-0.46231300
C	2.53756400	4.39211100	0.85134800
C	1.77389600	3.64690600	1.75189100
C	1.17589000	2.45113000	1.34526600
H	2.23166100	2.41481700	-1.90584700
H	3.29180700	4.53198500	-1.16553200
H	2.99655100	5.32285100	1.17104000
H	1.63673800	3.99524100	2.77151400
H	0.57411000	1.87510100	2.04147300
C	2.15449900	-0.95900900	-0.11021800
C	2.26429800	-1.51215000	1.16468300
C	3.37988000	-2.29336400	1.47092300
C	4.37924300	-2.53858800	0.51916500
C	4.22939700	-1.98580700	-0.76048100
C	3.12102100	-1.20210800	-1.08551600
H	1.49530300	-1.34341700	1.91248400
H	3.46953900	-2.72384900	2.46518200
H	4.98594100	-2.17535000	-1.51800800
H	3.01841500	-0.79357800	-2.08628300
C	5.59517500	-3.36338600	0.86747300
H	5.37608200	-4.08466200	1.65982500
H	5.96757300	-3.91411200	-0.00121300

H 6.41326600 -2.72500000 1.22367800
C -2.76050900 -0.70528100 -0.83234100
C -2.78517800 -1.98930000 -0.22553100
C -3.87307600 0.13800600 -0.63103900
C -3.87666400 -2.38420100 0.53784600
H -1.95209700 -2.66452000 -0.40216300
C -4.96111500 -0.27520600 0.14168500
H -3.88991100 1.12137600 -1.08940100
C -4.97790600 -1.54371800 0.73900900
H -3.88186700 -3.37343700 0.99035200
H -5.81461300 -1.87904100 1.33910100
O -1.73013300 -0.31006900 -1.56909100
O -5.97769900 0.64251300 0.26137300
C -7.11882600 0.27521800 1.01079700
H -7.79847200 1.12927100 0.97030000
H -7.62273300 -0.60388000 0.58642300
H -6.87151600 0.06464000 2.06057600

- $TS_{3\text{-}1a\text{-}Ph}$

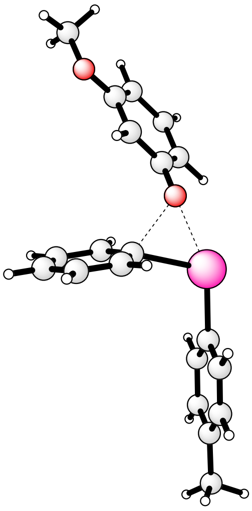

B3LYP/BS2 energy: -935.5499026890 a.u.
ZPE: 0.332506 a.u.
Thermal correction to Gibbs Free Energy: 0.275244 a.u.
Solvation energy: -7.58 kcal/mol
Dispersion correction: -60.05 kcal/mol

I 0.94862400 -0.95360100 -0.65483400
O -1.41680300 -0.42511500 -1.17440600
C -2.50334900 -0.74131300 -0.48272900
C -2.53592900 -1.84664900 0.40814300
C -3.66803800 0.04405800 -0.61181800
C -3.69921900 -2.14395600 1.10719500
H -1.65396900 -2.47186000 0.51055400
C -4.82478300 -0.26223400 0.10724800
H -3.66589900 0.89407700 -1.28484500
C -4.85380900 -1.36406700 0.97581900
H -3.71804300 -3.00136700 1.77561300
H -5.74447600 -1.61916000 1.53661400
O -5.88921500 0.57609600 -0.10495300
C -7.09623700 0.31063200 0.58439700
H -6.96620700 0.36346400 1.67393700
H -7.80070100 1.08418300 0.27220800
H -7.50783900 -0.67422100 0.32549000
C -0.17880600 1.10119300 -0.27557400
C 0.04323200 2.10160800 -1.21080200
C -0.58412500 1.33736800 1.02890400
C -0.17161400 3.42202100 -0.80209200
H 0.34422500 1.87109400 -2.22558400
C -0.78797000 2.66941600 1.40740600
H -0.77034900 0.52409300 1.72105000
C -0.57954500 3.71097700 0.50209800
H -0.01923700 4.22376000 -1.51971000
H -1.11294300 2.88090200 2.42253400
C 2.96832200 -0.56021400 -0.07484200
C 3.88838700 -0.09215900 -1.01839600
C 3.36526800 -0.75545200 1.25232200
C 5.20285100 0.16772900 -0.63021200
H 3.58711800 0.07061500 -2.04863500
C 4.68318000 -0.49109300 1.62377600
H 2.65527900 -1.10925000 1.99348600
C 5.62339200 -0.02934000 0.69204100
H 5.91243600 0.53212300 -1.36898800
H 4.98448500 -0.64441800 2.65716900
C 7.05555500 0.22419300 1.09735700
H 7.65440700 -0.69175400 1.01806200
H 7.52554600 0.97683500 0.45785700
H 7.12360100 0.56741200 2.13393000
H -0.73696700 4.74015000 0.80888300

- $TS_{3\text{-}1a\text{-}Ar}$

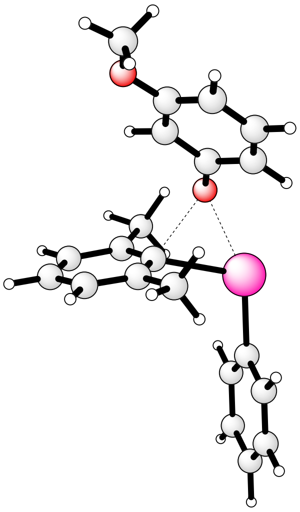

B3LYP/BS2 energy: -935.548642447 a.u.
ZPE: 0.332445 a.u.
Thermal correction to Gibbs Free Energy: 0.274601 a.u.
Solvation energy: -7.49 kcal/mol
Dispersion correction: -60.05 kcal/mol

I 1.31614300 -1.15661500 -0.51519400
O -1.04056300 -0.74404900 -1.15399700
C -2.14300700 -1.04050800 -0.47908800
C -2.17945300 -2.07694900 0.49086200
C -3.32252400 -0.30121600 -0.70803000
C -3.35968300 -2.35478600 1.16922900
H -1.28581100 -2.66704100 0.67093200
C -4.49642200 -0.58587600 -0.00830200
H -3.31762700 0.49610800 -1.44269100

C	-4.52862500	-1.62021300	0.93950900
H	-3.38080600	-3.16039300	1.89917000
H	-5.43237300	-1.85827400	1.48662000
O	-5.57358100	0.20417900	-0.31938200
C	-6.79670300	-0.04114600	0.34877800
H	-6.70482000	0.09584300	1.43483800
H	-7.50852400	0.68815200	-0.04310400
H	-7.17565400	-1.05301600	0.15150200
C	0.11426000	0.88922500	-0.32927100
C	0.33863600	1.82476900	-1.32807800
C	-0.34133900	1.21550600	0.93605700
C	0.07242700	3.16295300	-1.02750300
H	0.68321000	1.53260000	-2.31271700
C	-0.59426600	2.56684800	1.20051800
H	-0.52705800	0.45758500	1.68843900
C	-0.39278600	3.55923400	0.23474000
H	0.23074000	3.90980600	-1.80238900
H	-0.95676700	2.84203900	2.18820600
C	3.30510600	-0.66092000	0.10419500
C	4.23614200	-0.21668900	-0.84120900
C	3.66010200	-0.77402700	1.45296500
C	5.53108700	0.10698400	-0.43124000
H	3.95832600	-0.12628500	-1.88671500
C	4.95813700	-0.44884700	1.85174000
H	2.93468900	-1.11378300	2.18557400
C	5.89301500	-0.00906100	0.91235400
H	6.25590800	0.44951700	-1.16421500
H	5.23642700	-0.53908300	2.89785100
H	6.90130000	0.24347500	1.22686000
C	-0.69228200	5.01090900	0.52878500
H	0.07951900	5.67167100	0.12028600
H	-1.64815600	5.31837200	0.08675700
H	-0.75589500	5.19513400	1.60502500

- INT$_{3\text{-}1e\text{-}Ar}$

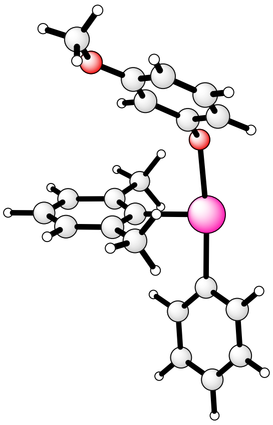

B3LYP/BS2 energy: -974.902204559 a.u.
ZPE: 0.362424 a.u.
Thermal correction to Gibbs Free Energy: 0.305344 a.u.
Solvation energy: -7.62 kcal/mol
Dispersion correction: -68.78 kcal/mol

I	0.99534100	-0.85418800	0.74628400
O	-1.07960400	-0.94718600	1.66428600
C	-2.12355500	-1.14562900	0.84869400
C	-2.35662600	-2.40649800	0.24575400
C	-3.03064100	-0.10394300	0.58518700
C	-3.46160500	-2.59333700	-0.57546300
H	-1.67504100	-3.22444300	0.46152300
C	-4.14344700	-0.30982600	-0.23743700
H	-2.87772800	0.87089800	1.03515300
C	-4.36968100	-1.55793200	-0.83161200
H	-3.63626500	-3.56775100	-1.02553200
H	-5.22615800	-1.73490100	-1.47040400
O	-4.95715200	0.78295800	-0.40707700
C	-6.11082600	0.63462500	-1.21224400
H	-6.79528500	-0.12512000	-0.81128200
H	-6.61267500	1.60447800	-1.20699900
H	-5.85719700	0.36834800	-2.24754900
C	0.39755300	1.12104100	0.13899200
C	0.14651300	1.35266800	-1.22236100
C	0.24173200	2.08838800	1.14489800
C	-0.22957800	2.65674600	-1.57708200
C	-0.13712700	3.37205300	0.72539500
C	-0.36311300	3.65658000	-0.61856900
H	-0.43127900	2.87393000	-2.62203500
H	-0.26347200	4.14803600	1.47491800
C	2.96979800	-0.54110600	-0.17195300
C	3.68556800	0.65075800	-0.03021200
C	3.53920700	-1.62421800	-0.84762200
C	4.96871200	0.75748400	-0.57212700
H	3.24673800	1.49908700	0.48702700
C	4.82610200	-1.51460000	-1.38344500
H	2.98526200	-2.55297100	-0.96831800
C	5.54054300	-0.32385900	-1.24684500
H	5.52253500	1.68611200	-0.46424300
H	5.26366900	-2.35716700	-1.91178900
H	-0.65912800	4.65768200	-0.91789100
H	6.53897100	-0.23728500	-1.66546500
C	0.41659200	1.79569500	2.61464800
H	-0.25156800	0.97954200	2.90858600
H	1.44249000	1.49142300	2.85253900
H	0.18274500	2.68059200	3.21108900
C	0.23878200	0.28491500	-2.28437600
H	1.25429200	-0.11527600	-2.37096500
H	-0.43151200	-0.55045500	-2.05528700
H	-0.04784100	0.69098000	-3.25711200

- INT$_{3\text{-}1e\text{-}Ph}$

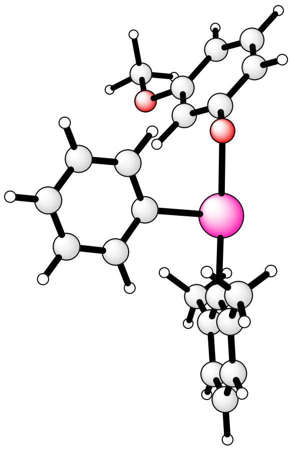

B3LYP/BS2 energy: -974.901388727 a.u.
ZPE: 0.362308 a.u.

Thermal correction to Gibbs Free Energy: 0.304185 a.u.
Solvation energy: -7.47 kcal/mol
Dispersion correction: -63.30 kcal/mol

I	0.56420000	-0.34896100	-0.82011800
O	-1.42125200	0.30178800	-1.76755800
C	-2.57403800	-0.14087100	-1.24722100
C	-3.55486800	-0.68793700	-2.11025000
C	-2.86684200	-0.06450900	0.12653700
C	-4.77049700	-1.11960400	-1.59812000
H	-3.33459000	-0.74666300	-3.17136300
C	-4.09487100	-0.51294200	0.62713000
H	-2.15089400	0.36149900	0.82198400
C	-5.06447200	-1.04480100	-0.22988200
H	-5.51670100	-1.53266600	-2.27277700
H	-6.01956100	-1.39635700	0.13992600
O	-4.25272800	-0.38186300	1.98549500
C	-5.48415000	-0.78708400	2.55205700
H	-5.67263500	-1.85852700	2.39968300
H	-5.40737900	-0.58781000	3.62304100
H	-6.32992000	-0.21934200	2.14120600
C	0.64278900	1.64139200	0.05338200
C	1.62861800	1.94909900	0.98548400
C	-0.31313200	2.55964900	-0.36646000
C	1.64712000	3.24108200	1.52115400
C	-0.27014700	3.84538600	0.18111600
C	0.70331000	4.18737100	1.12092700
H	2.40529500	3.49786100	2.25526600
H	-1.00781800	4.57722200	-0.13463500
C	2.49972700	-0.89591000	0.06367600
C	3.68620400	-0.61882500	-0.64649700
C	2.51135900	-1.63246700	1.26642500
C	4.89570300	-1.10130100	-0.12742500
C	3.74566300	-2.09388000	1.74681900
C	4.92652400	-1.83473800	1.05619700
H	5.81990400	-0.89354200	-0.66016900
H	3.77355000	-2.65956800	2.67428500
H	0.72604200	5.18826200	1.54155600
H	5.87302700	-2.20195900	1.44269900
C	1.25703100	-1.92592500	2.06177400
H	1.50695700	-2.42457100	3.00154400
H	0.70602100	-1.01120900	2.30454400
H	0.56574400	-2.57618900	1.51491200
C	3.70300600	0.19125800	-1.92398600
H	3.15354600	-0.30547800	-2.73104700
H	3.24342100	1.17522700	-1.78184000
H	4.72887700	0.34717900	-2.26677300
H	-1.06317000	2.25846100	-1.09047800
H	2.36531000	1.21705800	1.29419200

-TS$_{3-1e-Ph}$

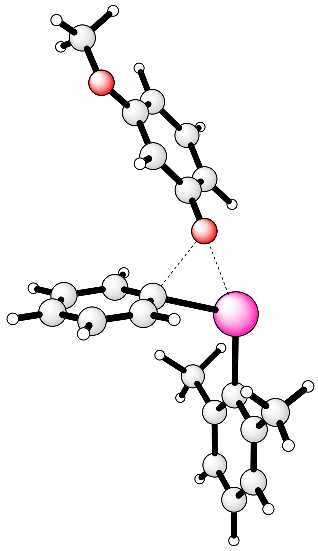

B3LYP/BS2 energy: -974.87465915 a.u.
ZPE: 0.360553 a.u.
Thermal correction to Gibbs Free Energy: 0.30235 a.u.
Solvation energy: -7.53 kcal/mol
Dispersion correction: -66.60 kcal/mol

I	1.05101800	-0.82020900	-0.62779500
O	-1.30378400	-0.27613600	-1.22604600
C	-2.41971100	-0.67778500	-0.63595500
C	-2.48692000	-1.88705800	0.10687800
C	-3.58529200	0.11289800	-0.72424500
C	-3.68107900	-2.27523400	0.70118500
H	-1.60528500	-2.51789800	0.17326000
C	-4.77345000	-0.28600500	-0.10937800
H	-3.55897300	1.04074000	-1.28456700
C	-4.83563800	-1.48929300	0.61010700
H	-3.72505700	-3.21077600	1.25360000
H	-5.75096600	-1.81679500	1.08736900
O	-5.83415000	0.56877300	-0.26815200
C	-7.07041700	0.21664400	0.32354600
H	-6.99331900	0.13460900	1.41630700
H	-7.76582900	1.02099100	0.07542100
H	-7.46093900	-0.72961400	-0.07464900
C	-0.13285000	1.16018500	-0.09428900
C	0.13922100	2.26348000	-0.89047300
C	-0.64202800	1.24604200	1.19294500
C	-0.12963100	3.52746700	-0.35453000
C	-0.89711000	2.52447000	1.70245800
C	-0.63871800	3.66385100	0.93883000
H	0.06153700	4.40649600	-0.96422300
H	-1.30448200	2.61632300	2.70565100
C	3.05577500	-0.44517700	0.07763900
C	4.01941400	0.03049600	-0.83486700
C	3.36055400	-0.68756800	1.43211900
C	5.31840400	0.25009000	-0.35602400
C	4.67461600	-0.45137600	1.85839400
C	5.64611700	0.01217900	0.97573000
H	6.07676700	0.61380300	-1.04388900
H	4.92996300	-0.63435400	2.89851200
H	-0.83674700	4.65026300	1.34598300

H	6.65869200	0.18970600	1.32627700
C	2.34035500	-1.18047800	2.43242400
H	2.80224300	-1.31913800	3.41304000
H	1.51628900	-0.46872500	2.54709100
H	1.90094600	-2.13668500	2.12874700
C	3.71928500	0.30974100	-2.29023400
H	3.36594100	-0.58465500	-2.81447900
H	2.94379800	1.07432600	-2.40410400
H	4.61637100	0.66530600	-2.80300700
H	-0.87493900	0.35826500	1.77007000
H	0.51810900	2.15239500	-1.89926500

-TS$_{3\text{-}1e\text{-}Ar}$

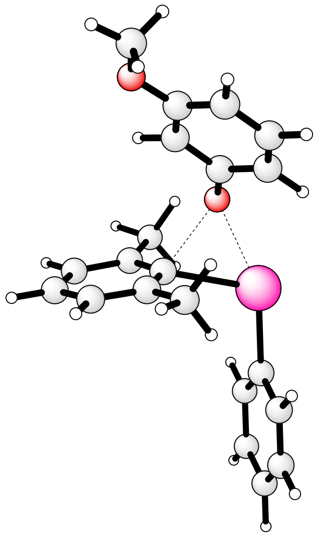

B3LYP/BS2 energy: -974.876689458 a.u.
ZPE: 0.360557 a.u.
Thermal correction to Gibbs Free Energy: 0.302654 a.u.
Solvation energy: -6.83 kcal/mol
Dispersion correction: -67.10 kcal/mol

I	-1.25630200	-0.91905500	-0.65132400
O	1.09559100	-0.31635900	-1.15024400
C	2.23833500	-0.75517700	-0.64056800
C	2.39442800	-2.09932500	-0.20843700
C	3.34144900	0.11481600	-0.52044300
C	3.61282700	-2.53000800	0.29983000
H	1.55807200	-2.78564700	-0.30575300
C	4.55493700	-0.33324900	0.00752700
H	3.24937700	1.14533400	-0.84467900
C	4.70649500	-1.66402200	0.42298500
H	3.72483800	-3.56498600	0.61400600
H	5.64186100	-2.02860000	0.82893100
O	5.54855700	0.61022100	0.07638800
C	6.80706200	0.21624400	0.58924300
H	7.26629200	-0.57651900	-0.01660100
H	7.44136000	1.10430800	0.55386500
H	6.73501500	-0.13146300	1.62878500
C	-0.12744400	1.14399200	-0.01093000
C	0.19280800	1.22848200	1.33915500
C	-0.25148100	2.20227400	-0.90593600
C	0.37442400	2.53779000	1.82334300
C	-0.06035700	3.47808000	-0.35140000
C	0.24428300	3.64970000	0.99760000
H	0.62378300	2.66149200	2.87404800
H	-0.13619100	4.33902800	-1.01065800
C	-3.26319200	-0.64936000	0.04867900
C	-4.16394700	0.12539100	-0.68985900
C	-3.66078900	-1.24679300	1.24989300
C	-5.46855400	0.29728100	-0.22242900
H	-3.85600300	0.58939000	-1.62171500
C	-4.96906800	-1.07279600	1.70622500
H	-2.96055000	-1.84585900	1.82408600
C	-5.87228300	-0.30078200	0.97298700
H	-6.16867300	0.89842300	-0.79552000
H	-5.27931300	-1.53989400	2.63656200
H	0.39167500	4.64699300	1.40016800
H	-6.88795000	-0.16491200	1.33246700
C	-0.51489100	2.02407400	-2.37709900
H	0.19055700	1.29850800	-2.78930300
H	-1.52745900	1.64682800	-2.56475800
H	-0.40767700	2.97458700	-2.90558400
C	0.33771500	0.04514800	2.26240500
H	-0.62888600	-0.44395100	2.43165000
H	1.02053300	-0.70271500	1.85198300
H	0.72362100	0.36811600	3.23239800

- INT$_{6\text{-}1a}$

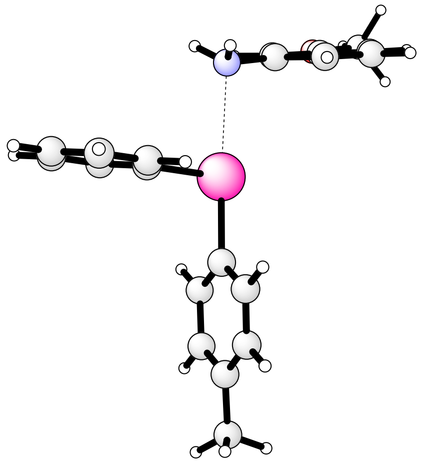

B3LYP/BS2 energy: -916.12811498 a.u.
ZPE: 0.359608 a.u.
Thermal correction to Gibbs Free Energy: 0.297426 a.u.
Solvation energy: -37.52 kcal/mol
Dispersion correction: -60.83 kcal/mol

I	0.31655800	0.18145400	-0.18505700
C	-2.96606700	0.96063000	-0.52308100
C	-3.28257600	0.50861700	-1.81325500
C	-3.59413700	0.40736300	0.59139400
C	-4.24948000	-0.48304500	-1.96160300
H	-2.79853700	0.94343800	-2.68345500
C	-4.56690400	-0.59213500	0.42755300
H	-3.37006700	0.74812100	1.59819300
C	-4.89785600	-1.04220800	-0.85859700
H	-4.51092800	-0.82658600	-2.95782700
H	-5.64789500	-1.80823200	-1.00793400
O	-5.12194300	-1.04977400	1.57622700
C	-6.14926100	-2.03503600	1.49469100

H	-5.77991200	-2.96241700	1.04105900
H	-6.45336000	-2.23248800	2.52268400
H	-7.01071400	-1.66740700	0.92527600
C	1.48247900	1.93571400	0.17275700
C	2.00766200	2.62048900	-0.92264000
C	1.68020800	2.34436000	1.49131900
C	2.77039400	3.76462700	-0.67402000
H	1.83927400	2.28207200	-1.93893500
C	2.44732400	3.49117400	1.71245500
H	1.25956900	1.79451100	2.32584200
C	2.98899500	4.19649900	0.63582000
H	3.19239900	4.31358900	-1.50990100
H	2.61791100	3.82787200	2.73012700
C	1.91019400	-1.23255300	-0.08526300
C	2.60613400	-1.55717600	-1.25206700
C	2.20010300	-1.84185200	1.13717100
C	3.62180800	-2.50886200	-1.17803100
H	2.37216000	-1.08507300	-2.20055200
C	3.22060100	-2.79120500	1.18213800
H	1.65242000	-1.58987800	2.03933000
C	3.94455100	-3.14167900	0.03262600
H	4.17086900	-2.76431500	-2.07998300
H	3.45522900	-3.26751600	2.12981600
C	5.02308400	-4.19353100	0.08836500
H	4.61394000	-5.17757200	-0.17062300
H	5.82798300	-3.97939900	-0.61989500
H	5.45611400	-4.27176300	1.08868000
H	3.58325900	5.08605200	0.81814500
N	-1.93245600	1.93014500	-0.34410100
H	-1.86721100	2.56851600	-1.13207000
H	-2.06088700	2.47744300	0.50250100

- TS$_{6\text{-}1a\text{-}Ph}$

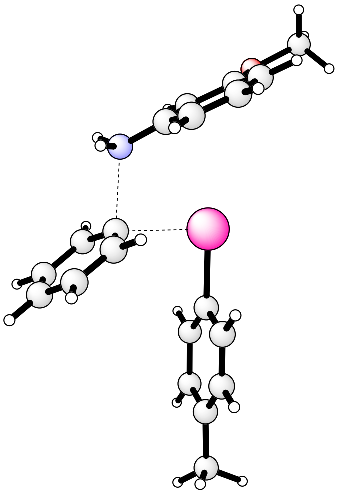

B3LYP/BS2 energy: -916.088077828 a.u.
ZPE: 0.35863 a.u.
Thermal correction to Gibbs Free Energy: 0.300842 a.u.
Solvation energy: -37.05 kcal/mol
Dispersion correction: -65.27 kcal/mol

I	0.13479600	-0.70828200	-0.08562000
C	2.68446700	1.34839800	0.20470100
C	2.99311100	1.25954500	1.57448100
C	3.22865800	0.43727800	-0.70904600
C	3.85993800	0.26018200	2.00064700
H	2.57505500	1.97103000	2.28001300
C	4.10483600	-0.56616000	-0.26230800
H	3.02123400	0.50858500	-1.77249800
C	4.41631700	-0.65756200	1.10357700
H	4.11645200	0.18941000	3.05289800
H	5.09179300	-1.42019300	1.46969000
O	4.58369400	-1.38426000	-1.22390300
C	5.51882600	-2.40292000	-0.86230700
H	5.07285900	-3.12412600	-0.16798000
H	5.77753700	-2.90812300	-1.79230600
H	6.42231700	-1.97094600	-0.41799000
C	-0.49127600	1.84919500	-0.24420800
C	-0.98471100	2.31258200	0.95866200
C	-0.96916100	2.17331400	-1.49769500
C	-2.08488300	3.18085800	0.88174000
H	-0.56201600	2.02542700	1.91468300
C	-2.06971000	3.04498500	-1.53265600
H	-0.54247500	1.77612400	-2.41158400
C	-2.62304100	3.54580400	-0.35362000
H	-2.50909600	3.56584800	1.80460400
H	-2.48324800	3.32097000	-2.49837600
C	-1.93279200	-1.12883100	0.04253300
C	-2.53049400	-1.21361600	1.30208700
C	-2.66434800	-1.33649400	-1.12887800
C	-3.89098000	-1.50841000	1.37754200
H	-1.95506200	-1.05801400	2.20796100
C	-4.02339900	-1.63034000	-1.02395700
H	-2.19380300	-1.27451000	-2.10393400
C	-4.65856000	-1.72446100	0.22327300
H	-4.36113900	-1.57541700	2.35474100
H	-4.59773800	-1.79285300	-1.93177000
C	-6.12055500	-2.08050600	0.32271700
H	-6.24808200	-3.16672300	0.40486200
H	-6.58367900	-1.63107200	1.20522700
H	-6.67449700	-1.75325900	-0.56104900
H	-3.47275400	4.21867700	-0.39689600
N	1.74614300	2.29365000	-0.24332200
H	1.65408300	3.11513500	0.34555300
H	1.84206600	2.56447100	-1.21722800

- TS$_{6\text{-}1a\text{-}Ar}$

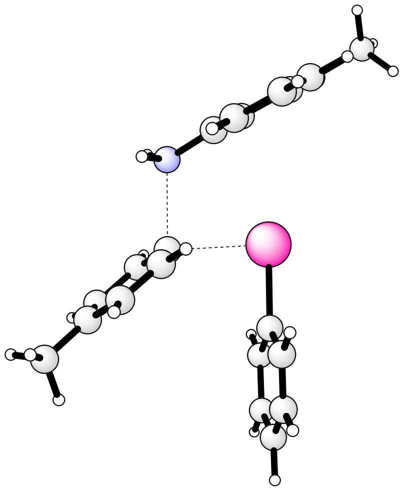

B3LYP/BS2 energy: -916.087154717 a.u.
ZPE: 0.358617 a.u.

Thermal correction to Gibbs Free Energy: 0.300697 a.u.
Solvation energy: -37.02 kcal/mol
Dispersion correction: -65.22 kcal/mol

I	0.26927000	-0.92907600	0.01174900
C	2.27790600	1.67471800	0.07694100
C	2.63987100	1.76349800	1.43396200
C	2.99569600	0.85035000	-0.79870000
C	3.73203400	1.03084100	1.88373300
H	2.08686200	2.40875200	2.10970500
C	4.09754300	0.11650200	-0.32827500
H	2.74608400	0.79044400	-1.85373300
C	4.46318900	0.20411600	1.02425600
H	4.02960300	1.10191000	2.92510400
H	5.31137200	-0.34863700	1.40781800
O	4.72692000	-0.63722100	-1.25528200
C	5.88146900	-1.38590200	-0.86911000
H	5.63465900	-2.13054600	-0.10390700
H	6.21898300	-1.89293100	-1.77248700
H	6.67613500	-0.72698300	-0.50205300
C	-0.95403100	1.39910900	-0.22209200
C	-1.47299300	1.81162300	0.98663000
C	-1.56140600	1.54122700	-1.45148200
C	-2.74242500	2.40698100	0.94219400
H	-0.94632800	1.68682900	1.92589100
C	-2.82913900	2.14304900	-1.44643900
H	-1.10908400	1.20533900	-2.37749800
C	-3.43807400	2.58084100	-0.26250800
H	-3.18548300	2.74073100	1.87701200
H	-3.34086200	2.26694000	-2.39747700
C	-1.64530200	-1.82771300	0.13373600
C	-2.24316000	-1.98167400	1.38661600
C	-2.25601900	-2.27350800	-1.04073500
C	-3.49552300	-2.59565200	1.45498200
H	-1.74882700	-1.64145500	2.28960900
C	-3.50796400	-2.88508900	-0.94908900
H	-1.77155100	-2.15714600	-2.00370400
C	-4.12600100	-3.04491800	0.29283600
H	-3.97229000	-2.72584800	2.42152200
H	-3.99422800	-3.24022000	-1.85232900
N	1.13052600	2.33890500	-0.38796000
H	0.86869300	3.16252200	0.14393100
H	1.12535800	2.54170600	-1.38276100
C	-4.81475800	3.20108300	-0.28264100
H	-5.59196700	2.43020800	-0.22116000
H	-4.96010400	3.88017500	0.56140800
H	-4.98594800	3.76370900	-1.20431900
H	-5.09691100	-3.52592300	0.35581600

- $INT_{6\text{-}2b}$

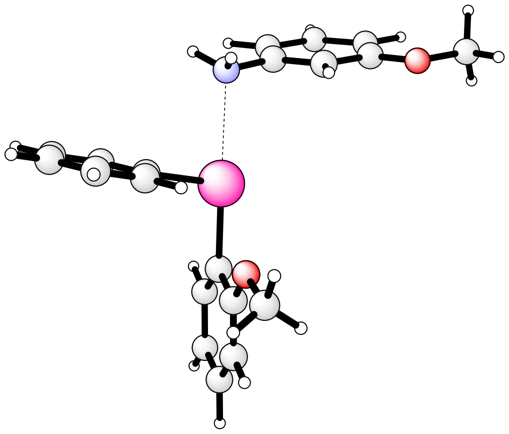

B3LYP/BS2 energy: -991.360811921 a.u.
ZPE: 0.365365 a.u.
Thermal correction to Gibbs Free Energy: 0.305086 a.u.
Solvation energy: -36.92 kcal/mol
Dispersion correction: -62.93 kcal/mol

I	-0.31605900	0.09111300	-0.27043500
C	2.87783900	1.28886000	-0.13876600
C	3.41052300	1.38537600	-1.43252600
C	3.44085100	0.41854700	0.79335200
C	4.52195500	0.61373800	-1.76238900
H	2.97606500	2.06745000	-2.15816300
C	4.56348500	-0.35148100	0.44841000
H	3.04684000	0.33383400	1.80205400
C	5.10840000	-0.25509800	-0.83963600
H	4.94872800	0.69326400	-2.75755000
H	5.97519100	-0.83702700	-1.12563800
O	5.03767200	-1.15326100	1.43343800
C	6.19965500	-1.93810000	1.17560700
H	7.06019200	-1.30704500	0.92513500
H	6.40449000	-2.47790400	2.10024700
H	6.02592300	-2.65727000	0.36636000
C	-1.65942900	1.70554800	0.11214800
C	-2.17635300	1.84833800	1.39895400
C	-1.96997900	2.57304500	-0.93462000
C	-3.04583800	2.91609200	1.63774100
H	-1.92726200	1.14631900	2.18558900
C	-2.84088400	3.63290600	-0.66858500
H	-1.55888100	2.43431000	-1.92835500
C	-3.37433300	3.80349200	0.61076500
H	-3.46434200	3.04880900	2.63048800
H	-3.10070900	4.32070500	-1.46712500
C	-1.78264600	-1.38618200	-0.69453100
C	-2.43044200	-2.03239500	0.37667400
C	-2.03495300	-1.72586300	-2.02405700
C	-3.35162800	-3.04337800	0.07138200
C	-2.95525000	-2.73456800	-2.30909900
C	-3.60469500	-3.38329700	-1.25841800
H	-3.87324700	-3.56357600	0.86542300
H	-3.15833800	-3.00750200	-3.33881600
H	-4.04975400	4.62996600	0.80722000
N	1.69844800	2.01855200	0.20639600
H	1.68548400	2.28485300	1.18726200

H	1.59058800	2.85847800	-0.35523300
H	-4.32268000	-4.16929500	-1.47056400
O	-2.11793600	-1.62235400	1.62958400
C	-2.69084100	-2.31836000	2.74299700
H	-2.26211100	-1.85592300	3.63165300
H	-3.78002000	-2.20588400	2.76035100
H	-2.42596300	-3.38038100	2.72079400
H	-1.51872400	-1.21345100	-2.82945700

- $TS_{6\text{-}2b\text{-}Ph}$

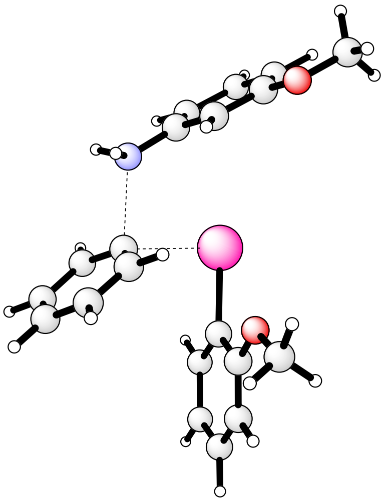

B3LYP/BS2 energy: -991.319091906 a.u.
ZPE: 0.364112 a.u.
Thermal correction to Gibbs Free Energy: 0.305749 a.u.
Solvation energy: -36.97 kcal/mol
Dispersion correction: -67.20 kcal/mol

I	-0.02448500	-0.39614400	-0.90752700
C	2.55296900	1.43873000	-0.07639100
C	2.88386400	1.32462400	-1.44077700
C	3.13646200	0.59200100	0.87123600
C	3.81429200	0.36294400	-1.82580800
H	2.44500500	1.99662000	-2.17214600
C	4.07344700	-0.36984200	0.46850800
H	2.89394300	0.66995500	1.92645700
C	4.40969100	-0.48793100	-0.89181400
H	4.09028800	0.27588600	-2.87185200
H	5.13329200	-1.22186600	-1.22302600
O	4.58803400	-1.12843900	1.46001200
C	5.57727700	-2.10822400	1.13935100
H	6.46426400	-1.64527100	0.69276600
H	5.84940700	-2.57249000	2.08680500
H	5.17711000	-2.87111400	0.46186900
C	-0.64072500	1.81648700	0.40160800
C	-0.94268500	1.53509100	1.71887800
C	-1.30399500	2.71462400	-0.41027300
C	-2.03999300	2.22285300	2.26020000
H	-0.39017800	0.80785900	2.30124700
C	-2.39538200	3.38220300	0.16815800
H	-1.02266000	2.89327900	-1.44174100
C	-2.75987200	3.14080200	1.49336000
H	-2.31862200	2.02731400	3.29175100
H	-2.95108400	4.09198000	-0.43778300
C	-2.04807800	-0.95614200	-0.75796100
C	-2.48967300	-1.63730800	0.39475900
C	-2.90823200	-0.67828400	-1.82091600
C	-3.83280400	-2.03593900	0.44599300
C	-4.24183300	-1.08021900	-1.75014800
C	-4.69247400	-1.75739200	-0.61709200
H	-4.20937900	-2.56427400	1.31338400
H	-4.91443000	-0.87048400	-2.57470600
H	-3.60424900	3.66565700	1.92721100
N	1.55954800	2.35112700	0.32289400
H	1.60678300	2.63260900	1.29711600
H	1.46763900	3.16705800	-0.27443600
H	-5.72741200	-2.07874000	-0.55378900
O	-1.58422000	-1.85643100	1.37779100
C	-1.97575400	-2.63880300	2.50844200
H	-1.08318100	-2.72892200	3.12735700
H	-2.76882000	-2.14316000	3.07942700
H	-2.30907300	-3.63632100	2.20310400
H	-2.53727700	-0.16051700	-2.69865000

- $TS_{6\text{-}2b\text{-}Ar}$

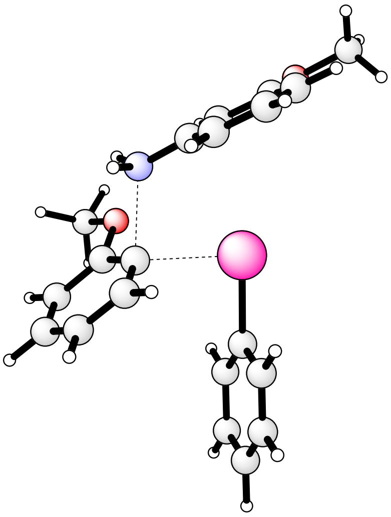

B3LYP/BS2 energy: -991.319759214 a.u.
ZPE: 0.364329 a.u.
Thermal correction to Gibbs Free Energy: 0.307039 a.u.
Solvation energy: -35.91 kcal/mol
Dispersion correction: -67.88 kcal/mol

I	0.03551400	-0.90202700	-0.20347400
C	2.28572700	1.09100200	1.03778400
C	2.69533300	0.45929700	2.22678300
C	2.86060000	0.73819000	-0.19164400
C	3.69436000	-0.50391800	2.16051400
H	2.24888200	0.73414100	3.17742800
C	3.87079300	-0.23692700	-0.23995300
H	2.56487300	1.23303600	-1.11110500
C	4.28492900	-0.86625300	0.94424000
H	4.02936600	-0.98829300	3.07226200
H	5.06328100	-1.61874500	0.93156100
O	4.36668700	-0.49396700	-1.47022600
C	5.42899100	-1.44046800	-1.60178900
H	5.11414800	-2.43968200	-1.27998000
H	5.67394100	-1.46266100	-2.66326400

H	6.31031100	-1.12851600	-1.03044800
C	-0.92121400	1.35894900	0.62667900
C	-1.67509200	1.19095000	1.76551700
C	-1.22764600	2.23365300	-0.41627600
C	-2.87395800	1.91957900	1.85646400
C	-2.43711000	2.93447100	-0.30489000
C	-3.24900600	2.77296400	0.82262800
H	-3.50057100	1.79576500	2.73377200
H	-2.73072800	3.62419700	-1.08859700
C	-1.94787900	-1.64118000	-0.22487700
C	-2.38063000	-2.43259400	0.84226200
C	-2.77146100	-1.35125600	-1.31508900
C	-3.68108200	-2.94040600	0.81093600
H	-1.72340000	-2.65817100	1.67489100
C	-4.06911400	-1.86614600	-1.32468800
H	-2.41406300	-0.74448100	-2.13939200
C	-4.52255500	-2.65720200	-0.26664800
H	-4.03082900	-3.56044100	1.63048100
H	-4.72162700	-1.65154000	-2.16529300
N	1.23093800	2.00963700	1.05023700
H	1.04745400	2.46006400	1.93969400
H	1.21841300	2.66285400	0.27374300
H	-5.53100600	-3.05806300	-0.28499100
H	-4.17699400	3.33096000	0.88871100
O	-0.31662700	2.36039200	-1.42019800
C	-0.66000600	3.17758700	-2.54684100
H	0.17836200	3.09428000	-3.23775500
H	-1.57196000	2.81344900	-3.03101000
H	-0.79027800	4.22461900	-2.25279200
H	-1.38071700	0.50402800	2.55126000

- INT$_{9\text{-}1d}$

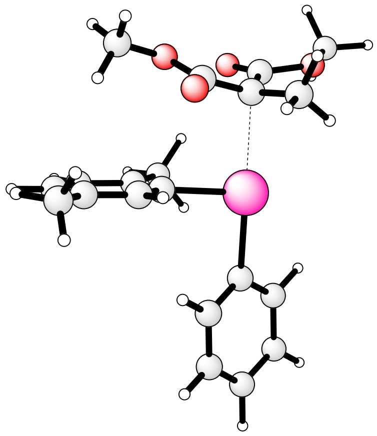

B3LYP/BS2 energy: -1088.50812807 a.u.
ZPE: 0.385728 a.u.
Thermal correction to Gibbs Free Energy: 0.322089 a.u.
Solvation energy: -10.00 kcal/mol
Dispersion correction: -71.50 kcal/mol

I	0.43743100	-0.84143000	-0.53847200
C	0.33570100	1.11753500	0.32691800
C	0.28671100	2.17735800	-0.57474500
C	0.33373000	1.27814400	1.72026200
C	0.26081400	3.49704600	-0.09865100
C	0.31267300	2.60681000	2.16678200
C	0.28110900	3.68828500	1.28552300
H	0.31110300	2.79097000	3.23770200
C	2.66462100	-0.79580800	-0.27006700
C	3.25478700	-1.95013300	0.24639300
C	3.45117700	0.28632900	-0.66813400
C	4.64626400	-2.02078000	0.37148800
H	2.64200900	-2.79308100	0.55930000
C	4.84061400	0.21087900	-0.53868600
H	2.99403800	1.18836700	-1.06435800
C	5.43811100	-0.94071900	-0.02010400
H	5.10669600	-2.91659300	0.77933600
H	5.45601300	1.05260500	-0.84505800
H	0.26168300	4.69890700	1.68516400
H	6.51842500	-0.99469300	0.07815400
C	0.32209000	0.13106900	2.69682600
H	-0.58692200	-0.46670000	2.56675600
H	1.18943600	-0.52371000	2.55666300
H	0.34627100	0.50423500	3.72351200
C	-2.18185900	-0.90637300	-0.78342300
C	-2.48388200	-1.70154600	0.42214600
C	-2.61017900	0.50535200	-0.85808000
O	-2.54927500	1.18365000	-1.87867600
O	-2.54428400	-1.33109600	1.58546000
O	-3.07316800	1.01915000	0.30778900
O	-2.61752500	-3.03158400	0.10446700
C	-2.23787700	-1.62531400	-2.12365800
H	-3.22533600	-2.06535700	-2.30650600
H	-2.02557300	-0.91530000	-2.92456200
H	-1.51960300	-2.45307600	-2.17891000
C	-3.52512300	2.37408000	0.23969400
H	-3.90105600	2.60582000	1.23718300
H	-2.70732300	3.05037100	-0.02244200
H	-4.32054400	2.48537500	-0.50273400
C	-2.85563500	-3.89663800	1.21829600
H	-3.78702500	-3.63235600	1.72684000
H	-2.92372200	-4.90309300	0.80244400
H	-2.04021100	-3.83919400	1.94529600
C	0.21059200	4.65481400	-1.06749600
H	1.15233800	4.75502900	-1.62004800
H	-0.58542200	4.51557300	-1.80637100
H	0.03028300	5.59927700	-0.54709300
H	0.23798600	1.98913200	-1.64128700

- TS$_{9\text{-}1d\text{-}Ph}$

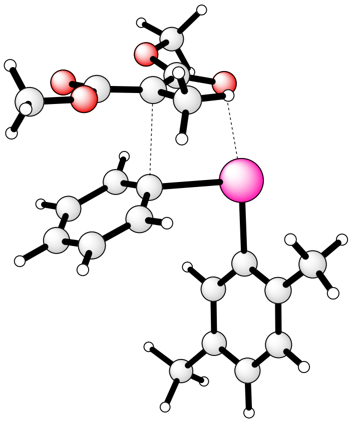

B3LYP/BS2 energy: -1088.48484677 a.u.
ZPE: 0.384185 a.u.

Thermal correction to Gibbs Free Energy: 0.322661 a.u.
Solvation energy: -10.40 kcal/mol
Dispersion correction: -72.56 kcal/mol

I	0.72342500	-1.46524400	0.07114600
C	-0.42508900	0.67290900	0.13476600
C	-0.10410600	1.49174100	-0.93739700
C	-0.74621200	1.15663900	1.39576400
C	-0.16034500	2.87640500	-0.74237400
C	-0.79375400	2.54440100	1.56663300
C	-0.49855100	3.40307500	0.50585900
H	0.06476100	3.53675000	-1.57550600
H	-1.07562500	2.94529100	2.53617500
C	2.56564100	-0.38968500	-0.12139200
C	3.39773100	-0.60268500	-1.23421900
C	2.91878400	0.49047500	0.90471900
C	4.60069800	0.11943400	-1.26271400
C	4.12176000	1.20317500	0.85929100
C	4.95711100	1.00152800	-0.24615400
H	-0.53130800	4.47839800	0.65174200
H	5.89771600	1.54253400	-0.31392600
C	-2.70678900	-0.59175400	-0.64707700
C	-2.45013600	-1.48553300	0.42422300
C	-3.49616200	0.62304500	-0.46500000
O	-3.88717000	1.12143700	0.57899100
O	-1.60779300	-2.42437900	0.34700600
O	-3.72592200	1.23477700	-1.67569800
O	-3.09469000	-1.26769500	1.59706700
C	-2.30361500	-1.00856900	-2.03582400
H	-3.17933000	-1.07211800	-2.69168400
H	-1.62712400	-0.28390300	-2.50970500
H	-1.81535400	-1.98352000	-2.01284000
C	-4.42790900	2.47490400	-1.58955400
H	-4.54926700	2.82093800	-2.61763400
H	-5.40479800	2.34393200	-1.11503600
H	-3.85957000	3.20656200	-1.00700000
C	-2.83264500	-2.20869700	2.64101800
H	-1.77603700	-2.21683200	2.92513900
H	-3.44410100	-1.88113500	3.48300000
H	-3.11663300	-3.22182100	2.34147800
H	0.16201500	1.08306400	-1.90642500
H	-1.00823200	0.49269400	2.21136800
C	3.06112200	-1.54822200	-2.36354400
H	3.00950800	-2.58861500	-2.02267200
H	2.09200100	-1.31425800	-2.81666700
H	3.82088400	-1.49405400	-3.14714700
C	4.51378800	2.13970600	1.97757000
H	3.64428500	2.44658000	2.56526800
H	5.22383100	1.66180500	2.66375200
H	4.99620300	3.04254200	1.59004800
H	2.24358500	0.64128900	1.74154900
H	5.26772700	-0.01504700	-2.11050300

- $TS_{9\text{-}1d\text{-}Ar}$

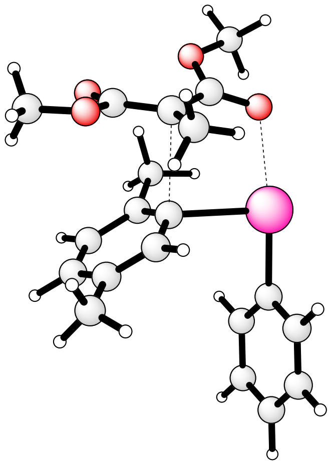

B3LYP/BS2 energy: -1088.48586239 a.u.
ZPE: 0.384598 a.u.
Thermal correction to Gibbs Free Energy: 0.32376 a.u.
Solvation energy: -9.38 kcal/mol
Dispersion correction: -73.27 kcal/mol

I	1.02454600	-1.50831300	-0.40035500
C	-0.09717400	0.59072100	0.24523400
C	0.28276400	1.60512700	-0.61696600
C	-0.40384300	0.76297700	1.59385800
C	0.31477100	2.92806600	-0.14724800
C	-0.34946400	2.09607600	2.03737600
C	-0.00195800	3.15225700	1.19663200
C	2.87591100	-0.49963200	-0.08085000
C	3.75145200	-0.35073600	-1.16059700
C	3.22226600	-0.03027700	1.18938200
C	4.98631000	0.27178900	-0.96251300
C	4.45630000	0.59602200	1.37379800
C	5.33910000	0.74542800	0.30199800
H	4.72684900	0.96501900	2.35891500
H	0.03084600	4.16310200	1.59411900
H	6.29905000	1.23032800	0.45246500
C	-2.41696100	-0.39388000	-0.90206100
C	-2.21999600	-1.68577800	-0.33863600
C	-3.24747900	0.60447800	-0.24014400
O	-3.61085600	0.62968300	0.92874200
O	-1.30776400	-2.47013900	-0.71500900
O	-3.55213800	1.63370100	-1.09696200
O	-3.05645100	-2.06965700	0.65697700
C	-1.98311000	-0.17182300	-2.32676800
H	-2.84919200	-0.15259700	-3.00111400
H	-1.47567000	0.79014900	-2.45802900
H	-1.32114200	-0.97625900	-2.65232400
C	-4.29189600	2.70300800	-0.50611500
H	-4.46756000	3.42216900	-1.30818900
H	-5.24326700	2.34737800	-0.10020500
H	-3.72576900	3.16978400	0.30578600
C	-2.81143800	-3.35788600	1.22485600
H	-1.83154300	-3.40266400	1.71025300
H	-3.59966500	-3.50126800	1.96518900
H	-2.85842700	-4.14180200	0.46398600
H	3.47676700	-0.71195900	-2.14702400
H	5.66796800	0.38824600	-1.80014400

H	2.53607400	-0.13680400	2.02285600
H	0.54451900	1.39403800	-1.64873600
H	-0.60568400	2.29701700	3.07498600
C	-0.85947900	-0.33398900	2.51938400
H	-0.73249100	-0.03582000	3.56371900
H	-1.92059000	-0.53405800	2.34166600
H	-0.30168100	-1.26158200	2.35619900
C	0.66945500	4.06150800	-1.08079600
H	1.46681700	3.77609900	-1.77422800
H	-0.19587600	4.36004600	-1.68501200
H	1.00323800	4.94257100	-0.52525000

- INT$_{9\text{-}1e\text{-}Ar}$

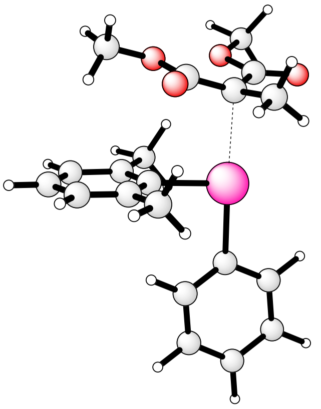

B3LYP/BS2 energy: -1088.50614479 a.u.
ZPE: 0.386516 a.u.
Thermal correction to Gibbs Free Energy: 0.326000 a.u.
Solvation energy: -9.67 kcal/mol
Dispersion correction: -74.10 kcal/mol

I	0.50135400	-0.77102500	-0.40871000
C	0.25864700	1.28389600	0.18685800
C	0.33679500	2.27203000	-0.80954200
C	0.04191000	1.55253400	1.54872600
C	0.21916000	3.60352800	-0.37947200
C	-0.07012600	2.90233300	1.91214200
C	0.02191000	3.91636300	0.96234300
H	0.28043400	4.39556400	-1.11997600
H	-0.23461300	3.14821200	2.95715400
C	2.71293300	-0.61668900	-0.04537700
C	3.37366300	-1.84482500	0.00289800
C	3.42004600	0.57763900	0.08064000
C	4.75932700	-1.87861400	0.18147300
H	2.82158900	-2.77841600	-0.09394600
C	4.80721700	0.53618100	0.26045000
H	2.90870600	1.53423900	0.04561400
C	5.47651200	-0.68755200	0.31071800
H	5.27439200	-2.83449200	0.22236100
H	5.36223600	1.46519200	0.36031400
H	-0.06492600	4.95479800	1.26861200
H	6.55321400	-0.71350800	0.45052000
C	-0.08904400	0.48048100	2.60167300
H	-0.94327400	-0.16810200	2.38241000
H	0.80850300	-0.14493100	2.65606600
H	-0.24339700	0.93167500	3.58464800
C	0.51523200	1.97771600	-2.27843200
H	1.38879300	1.34599600	-2.46840500
H	-0.37399500	1.47017900	-2.66649700
H	0.64464000	2.90784800	-2.83665000
C	-2.09563200	-1.03416400	-0.90287500
C	-2.34832600	-2.03062700	0.15162100
C	-2.64772500	0.32960000	-0.93015700
O	-2.66236500	1.03446300	-1.93652400
O	-2.13460800	-3.22750200	-0.01060400
O	-3.10761300	0.80011200	0.25871800
O	-2.75920800	-1.55270100	1.36256600
C	-1.95975100	-1.67217000	-2.27805500
H	-2.89277000	-2.16523700	-2.57807200
H	-1.72247000	-0.91468300	-3.02582800
H	-1.18951100	-2.45070900	-2.28424000
C	-3.62147600	2.13545100	0.21987900
H	-3.97932300	2.33971100	1.23012200
H	-2.84118000	2.84888300	-0.05819700
H	-4.44177100	2.21905100	-0.49816300
C	-2.98432200	-2.56100800	2.35472000
H	-2.06138500	-3.09840100	2.59185900
H	-3.35068300	-2.02868100	3.23385300
H	-3.72634100	-3.28745200	2.01341700

- INT$_{9\text{-}1e\text{-}Ph}$

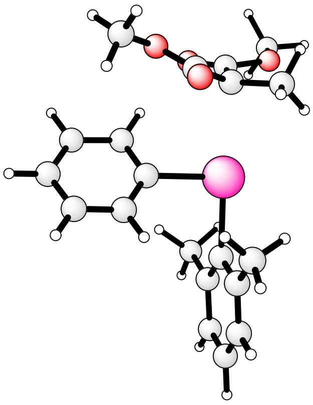

B3LYP/BS2 energy: -1088.50494339 a.u.
ZPE: 0.385958 a.u.
Thermal correction to Gibbs Free Energy: 0.324832 a.u.
Solvation energy: -9.87 kcal/mol
Dispersion correction: -72.02 kcal/mol

I	-0.34741300	-0.65697800	0.29061200
C	-0.15437400	1.39593900	-0.36280800
C	-1.08347400	2.32485800	0.10103700
C	0.89233000	1.72973600	-1.21694100
C	-0.95895600	3.65026600	-0.32112700
C	0.99150900	3.06570800	-1.62532000
C	0.07478500	4.01980400	-1.18446200
H	-1.67223300	4.38943100	0.03143800
H	1.79816400	3.34843300	-2.29517000
C	-2.57004600	-0.50554000	0.05950800
C	-3.35765400	-0.47528400	1.22514100
C	-3.13430000	-0.56442400	-1.22792000
C	-4.75273700	-0.48260000	1.07104200
C	-4.53298300	-0.56943700	-1.32960800
C	-5.33580700	-0.52802700	-0.19227700
H	-5.38130600	-0.45180700	1.95726800

H	-4.99003800	-0.60691100	-2.31506400
H	0.16487300	5.05177900	-1.51060900
H	-6.41756800	-0.53418900	-0.29074800
C	2.28870700	-0.93099300	0.68489800
C	2.75724100	-1.31663400	-0.65500800
C	2.70875300	0.34887700	1.28904100
O	2.44981300	0.67720100	2.44070000
O	2.98521800	-0.59909600	-1.62272100
O	3.42224000	1.16262700	0.46538800
O	2.85321400	-2.68001700	-0.76846700
C	2.11757300	-2.04544400	1.70592900
H	3.04751800	-2.60736500	1.85145100
H	1.82131000	-1.61530900	2.66401800
H	1.36490900	-2.78371600	1.39622300
C	3.81553900	2.40968100	1.04524200
H	4.40423400	2.91576500	0.27862200
H	2.94243500	3.01230300	1.31193600
H	4.41586400	2.25236700	1.94530700
C	3.24066400	-3.15555000	-2.06058900
H	4.23020400	-2.78016900	-2.33552200
H	3.25646000	-4.24371900	-1.98248200
H	2.52789600	-2.83897400	-2.82770900
H	-1.89305800	2.03300200	0.75938800
H	1.62889200	0.99849200	-1.53892200
C	-2.29944400	-0.61294200	-2.48739800
H	-1.63827500	0.25610700	-2.56809200
H	-1.66248100	-1.50408100	-2.51527200
H	-2.93878400	-0.63199000	-3.37341600
C	-2.77535500	-0.43331700	2.62319800
H	-2.22464600	-1.34922000	2.86502800
H	-2.07627300	0.39893400	2.75692300
H	-3.57092000	-0.32309800	3.36430700

- TS$_{9\text{-}1e\text{-}Ph}$

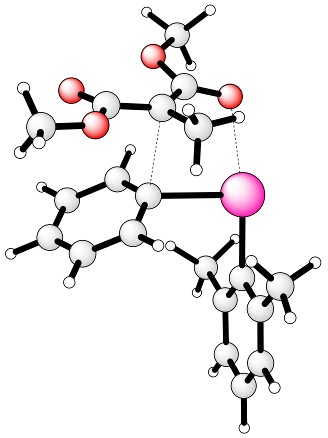

B3LYP/BS2 energy: -1088.48177252 a.u.
ZPE: 0.384444 a.u.
Thermal correction to Gibbs Free Energy: 0.324696 a.u.
Solvation energy: -10.30 kcal/mol
Dispersion correction: -74.67 kcal/mol

I	0.89841000	-1.30848600	-0.30323300
C	-0.41712200	0.59590500	0.48609200
C	-0.10890400	1.78218300	-0.16000500
C	-0.86623700	0.53536500	1.79776100
C	-0.30698200	2.97858000	0.54043200
C	-1.05650000	1.74325300	2.47753800
C	-0.77427500	2.96120700	1.85559200
H	-0.09255500	3.92175000	0.04513000
H	-1.43571200	1.72055300	3.49527400
C	2.68618300	-0.13690500	-0.13512200
C	3.26144100	0.38094700	-1.31392700
C	3.27114100	0.05352100	1.13340600
C	4.44408700	1.12360600	-1.18927000
C	4.45371000	0.80249500	1.19910800
C	5.03459300	1.33692800	0.05259600
H	4.90219900	1.53389500	-2.08491500
H	4.91800200	0.96415700	2.16796100
H	-0.91909800	3.89277300	2.39384600
H	5.95072000	1.91563600	0.12666900
C	-2.49516600	-0.42570500	-0.94401900
C	-2.30823700	-1.59134800	-0.15211700
C	-3.35379600	0.67158800	-0.51040500
O	-3.90175800	0.82127900	0.57030900
O	-1.39648800	-2.43411900	-0.37755700
O	-3.44405400	1.63203000	-1.49293500
O	-3.11537300	-1.75847700	0.92288000
C	-1.92313600	-0.41400900	-2.33596700
H	-2.68976900	-0.12833800	-3.06266000
H	-1.10802000	0.31680800	-2.45751600
H	-1.53696800	-1.39993400	-2.59977800
C	-4.20424500	2.78384300	-1.12781100
H	-4.20321200	3.43226900	-2.00596500
H	-5.22878000	2.51218100	-0.85758900
H	-3.74980600	3.29795000	-0.27527900
C	-2.91073700	-2.95360500	1.68032100
H	-1.89946600	-3.00264200	2.09537200
H	-3.64555700	-2.91322200	2.48552500
H	-3.07334400	-3.84396300	1.06589400
H	0.24445200	1.80094500	-1.18567300
H	-1.10869000	-0.40822800	2.27259300
C	2.68385600	-0.50540500	2.40789000
H	1.69724300	-0.07887000	2.61419900
H	2.56340600	-1.59281800	2.35473800
H	3.33233000	-0.27970700	3.25797600
C	2.68021800	0.16094300	-2.69234100
H	2.68526500	-0.89925200	-2.96908600
H	1.64130400	0.49901700	-2.76381000
H	3.26239300	0.70277100	-3.44162800

- $TS_{9\text{-}1e\text{-}Ar}$

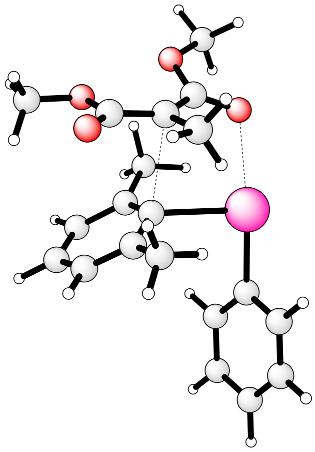

B3LYP/BS2 energy: -1088.47821554 a.u.
ZPE: 0.385130 a.u.
Thermal correction to Gibbs Free Energy: 0.325029 a.u.
Solvation energy: -9.64 kcal/mol
Dispersion correction: -75.75 kcal/mol

I -1.01159900 -1.41868000 0.04212400
C 0.08709200 0.79640100 -0.17534400
C -0.29929800 1.73637300 0.78115800
C 0.48799900 1.09097100 -1.48105100
C -0.26099300 3.08037200 0.36717300
C 0.49720100 2.45409900 -1.82811800
C 0.12639800 3.43976900 -0.91872800
H -0.53968400 3.84409100 1.08867800
H 0.80976100 2.72468200 -2.83350200
C -2.88957100 -0.40967700 -0.07224500
C -3.84019600 -0.66823000 0.91973800
C -3.17940600 0.45194000 -1.13346700
C -5.09478900 -0.05848000 0.84316700
C -4.43508100 1.05948700 -1.19584500
C -5.39293700 0.80465700 -0.21213700
H -5.83423300 -0.25731800 1.61349300
H -4.66250400 1.73231900 -2.01763300
H 0.13459100 4.48485500 -1.21342700
H -6.36841100 1.27852700 -0.26767700
C 2.32373200 -0.55835600 1.14321600
C 2.27896500 -1.56809200 0.14251900
C 3.03503700 0.70571900 1.07364500
O 3.08473300 1.52007300 1.99287500
O 1.36385300 -2.43384500 0.11595600
O 3.59631000 0.97953800 -0.14201100
O 3.26182100 -1.60949900 -0.79522900
C 1.72310900 -0.93759400 2.47455900
H 1.97697200 -0.17658800 3.21336600
H 0.63303200 -1.04768200 2.44751400
H 2.11193600 -1.90478900 2.81659500
C 4.21620900 2.26311400 -0.23650000
H 4.65791400 2.30442400 -1.23385400
H 3.48062600 3.06424500 -0.11539000
H 4.98990600 2.38597100 0.52633900
C 3.19547900 -2.69570400 -1.72323600
H 2.29875100 -2.63957200 -2.34759200
H 4.08824500 -2.60070800 -2.34322900
H 3.19587500 -3.65766200 -1.20362200
H -3.60933700 -1.33332300 1.74653400
H -2.43499400 0.66270300 -1.89364400
C -0.67866300 1.44100000 2.21053400
H 0.21978800 1.44213400 2.83466100
H -1.35612200 2.21114700 2.58874200
H -1.16517500 0.47148100 2.32562300
C 0.96973100 0.08284700 -2.49291600
H 0.89095900 0.48934400 -3.50475000
H 2.01967200 -0.15150100 -2.29721600
H 0.40333400 -0.85058200 -2.45204800

- INT-NH_2

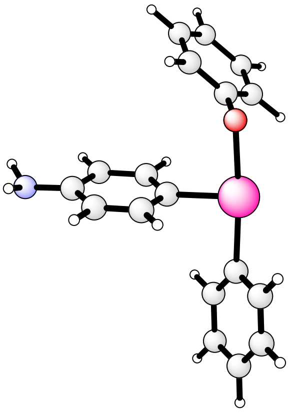

B3LYP/BS2 energy: -837.06414942 a.u.
ZPE: 0.290591 a.u.
Thermal correction to Gibbs Free Energy: 0.236844 a.u.
Solvation energy: -9.87 kcal/mol
Dispersion correction: -55.76 kcal/mol

I 0.52819000 -1.07533500 -0.52027500
O -1.59584500 -1.27673600 -1.29954300 C -2.64871900 -1.24997900 -0.47907700
C -2.78973500 -2.14028800 0.61202800
C -3.69177400 -0.32106500 -0.70350100
C -3.91341800 -2.09282800 1.43600700
H -2.01338200 -2.88085300 0.78841000
C -4.81647000 -0.29114000 0.11803100
H -3.59240800 0.36008000 -1.54364400
C -4.93656100 -1.17024800 1.19887900
H -3.99656700 -2.79205800 2.26513900
H -5.81353400 -1.14263200 1.83905800
C 0.06266600 0.97727800 -0.26966600
C 0.64450100 1.92338700 -1.11425800
C -0.85802900 1.35403100 0.70944100
C 0.31372600 3.26557000 -0.96436600
H 1.35303400 1.62731600 -1.88022000
C -1.18527500 2.69963800 0.84991100
H -1.33076000 0.61650700 1.34681800
C -0.60776600 3.67615400 0.01836100
H 0.77062800 4.00539400 -1.61643400

H	-1.90073600	2.99572600	1.61228200
C	2.58205000	-0.68948300	0.16219000
C	3.63661400	-1.15941900	-0.62486100
C	2.84419800	-0.07187700	1.38887300
C	4.95608100	-1.00737100	-0.18629300
H	3.44077300	-1.63805600	-1.58187500
C	4.16295400	0.08259700	1.82053600
H	2.02777500	0.29821100	2.00302800
C	5.21939200	-0.38558700	1.03452300
H	5.77456600	-1.36972600	-0.80229900
H	4.36516500	0.56590100	2.77254600
H	-5.60715400	0.42799700	-0.08502300
N	-0.89946500	5.02151900	0.19617800
H	-0.72814100	5.62250300	-0.59717500
H	-1.77101700	5.22900600	0.66239400
H	6.24408700	-0.26550100	1.37411100

- TS-NH$_2$-Ph

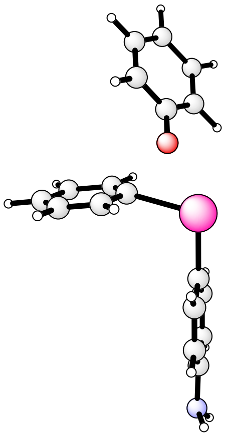

B3LYP/BS2 energy: -837.04028536 a.u.
ZPE: 0.289219 a.u.
Thermal correction to Gibbs Free Energy: 0.23719 a.u.
Solvation energy: -8.94 kcal/mol
Dispersion correction: -54.77 kcal/mol

I	0.37716900	-0.92692000	-0.63451400
O	-1.99958000	-0.32996500	-1.09503100
C	-3.09128600	-0.60473300	-0.39657000
C	-3.13665000	-1.64529900	0.56464900
C	-4.26575000	0.16678900	-0.58642600
C	-4.30393400	-1.90903100	1.27955400
H	-2.25025300	-2.25510400	0.71841900
C	-5.42091000	-0.10071400	0.13883000
H	-4.23357000	0.96770400	-1.31879100
C	-5.45265700	-1.14065000	1.07645600
H	-4.31636800	-2.72161300	2.00229600
H	-6.35964300	-1.34855700	1.63657100
C	-0.70101800	1.15130500	-0.21161500
C	-0.47296800	2.15882700	-1.13822100
C	-1.07031800	1.38380800	1.10448500
C	-0.64242400	3.47943700	-0.70908100
H	-0.19887600	1.93365600	-2.16194600
C	-1.23007900	2.71549600	1.50422200
H	-1.26390800	0.56727800	1.79093900
C	-1.01358200	3.76287900	0.60728300
H	-0.48299400	4.28614200	-1.41972200
H	-1.52637400	2.92268300	2.52904000
C	2.41157000	-0.59775800	-0.10887300
C	3.32816300	-0.15082200	-1.06922000
C	2.84389900	-0.81178100	1.20627200
C	4.65752800	0.06595900	-0.72409500
H	3.00967800	0.02473400	-2.09227000
C	4.17283500	-0.59574100	1.55269700
H	2.14581800	-1.15278100	1.96463700
C	5.10355300	-0.15334500	0.59323200
H	5.36048900	0.41238500	-1.47757400
H	4.49770200	-0.76553000	2.57618400
H	-1.13506600	4.79199700	0.93037200
H	-6.30935300	0.50408400	-0.02712200
N	6.41753500	0.11087900	0.94995400
H	6.75630200	-0.34200800	1.78627200
H	7.09454300	0.11748100	0.20100600

- TS-NH$_2$-Ar

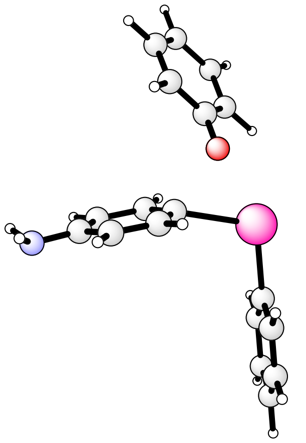

B3LYP/BS2 energy: -837.03560737 a.u.
ZPE: 0.289077 a.u.
Thermal correction to Gibbs Free Energy: 0.236602 a.u.
Solvation energy: -8.48 kcal/mol
Dispersion correction: -54.65 kcal/mol

- INT-OH

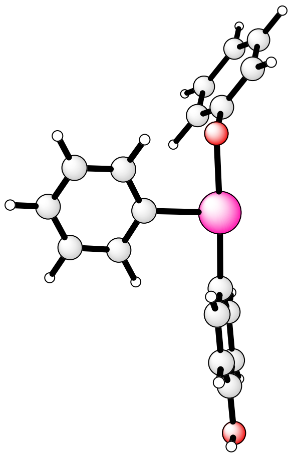

B3LYP/BS2 energy: -856.936628494 a.u.
ZPE: 0.278592 a.u.

Thermal correction to Gibbs Free Energy: 0.226545 a.u.
Solvation energy: -8.69 kcal/mol
Dispersion correction: -54.17 kcal/mol

I	0.00536900	-0.54335600	-0.60648600
O	-2.17951500	-0.29128600	-1.18891700
C	-3.15237600	-0.65570300	-0.33811000
C	-4.22186000	-1.44470200	-0.81693600
C	-3.17510800	-0.26998300	1.02028400
C	-5.26611700	-1.81969100	0.02437700
H	-4.20932000	-1.74174600	-1.86165100
C	-4.22280800	-0.65883900	1.85547600
H	-2.37132900	0.34837300	1.41067600
C	-5.27676400	-1.43438400	1.36878300
H	-6.07869100	-2.42400100	-0.37242100
H	-6.09057000	-1.73316100	2.02287100
C	-0.02977500	1.57941300	-0.13629800
C	0.99303300	2.13920800	0.62118000
C	-1.09795100	2.32078700	-0.62861000
C	0.93582100	3.50988200	0.89321800
H	1.81400200	1.54095000	0.99678700
C	-1.12835100	3.69051000	-0.34964100
H	-1.88179200	1.82398200	-1.19043300
C	-0.11772600	4.28506700	0.40722100
H	1.72097700	3.96341200	1.49121100
H	-1.95279300	4.28754100	-0.72818700
C	2.12249800	-0.68465700	-0.11649700
C	3.09772500	-0.36210900	-1.06695100
C	2.51859700	-1.22525500	1.11503100
C	4.44923100	-0.56247900	-0.78881800
H	2.81164600	0.05192200	-2.03000400
C	3.86620500	-1.42998500	1.40071200
H	1.77339700	-1.48801700	1.86132300
C	4.83620900	-1.09792300	0.44657000
H	5.20245100	-0.30764800	-1.53176600
H	4.18548400	-1.84654500	2.35039600
H	-0.15249100	5.34903900	0.62165600
H	-4.21535600	-0.34645400	2.89736700
O	6.13828800	-1.32022400	0.77655600
H	6.70676900	-1.06264600	0.03885400

- TS-OH-Ph

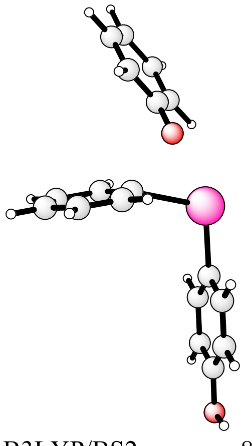

B3LYP/BS2 energy: -856.910274185 a.u.
ZPE: 0.276865 a.u.

Thermal correction to Gibbs Free Energy: 0.225072 a.u.
Solvation energy: -8.50 kcal/mol
Dispersion correction: -53.59 kcal/mol

I	0.37453600	-0.93064300	-0.62971100
O	-1.98690500	-0.32747200	-1.09463900
C	-3.08122700	-0.60198500	-0.39883100
C	-3.12672400	-1.63354900	0.57161500
C	-4.25740300	0.16282200	-0.60296600
C	-4.29668800	-1.89506900	1.28303500
H	-2.23906900	-2.23870100	0.73589200
C	-5.41511800	-0.10234100	0.11892600
H	-4.22454400	0.95652500	-1.34306200
C	-5.44720200	-1.13324100	1.06648200
H	-4.30965300	-2.70069900	2.01337600
H	-6.35618300	-1.33942900	1.62389800
C	-0.68576000	1.15712200	-0.20954900
C	-0.45180400	2.16088000	-1.13822500
C	-1.05826100	1.39170800	1.10487800
C	-0.61857900	3.48289100	-0.71181200
H	-0.17638500	1.93279700	-2.16091300
C	-1.21488300	2.72482600	1.50147100
H	-1.25655600	0.57708700	1.79221100
C	-0.99249900	3.76964400	0.60297800
H	-0.45534200	4.28775000	-1.42362100
H	-1.51346000	2.93487100	2.52497900
C	2.41446700	-0.60593100	-0.10058900
C	3.32768300	-0.16169200	-1.06236600
C	2.84161300	-0.81984900	1.21750700
C	4.65950100	0.05784400	-0.71340400
H	3.00991600	0.01263000	-2.08559300
C	4.16956300	-0.60215100	1.56863400
H	2.13975000	-1.15892200	1.97295300
C	5.08386200	-0.16182000	0.60266600
H	5.36764800	0.40015200	-1.46510300
H	4.51676900	-0.76625800	2.58330000
H	-1.11206200	4.79967800	0.92373000
H	-6.30516800	0.49696700	-0.05752200
O	6.36952300	0.03396000	1.00481700
H	6.89974500	0.33706900	0.25606600

- TS-OH-Ar

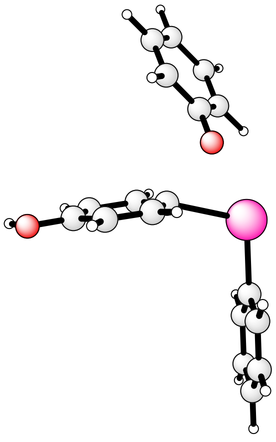

B3LYP/BS2 energy: -856.906921924 a.u.
ZPE: 0.276642 a.u.

Thermal correction to Gibbs Free Energy: 0.224474 a.u.
Solvation energy: -8.23 kcal/mol
Dispersion correction: -53.54 kcal/mol

I	0.77379600	-1.14910900	-0.51800500
O	-1.59444100	-0.70553100	-1.12836100
C	-2.69557300	-0.98900800	-0.44900600
C	-2.73167600	-1.98581100	0.55814300
C	-3.89062800	-0.27051900	-0.70788000
C	-3.90949500	-2.25918400	1.25161100
H	-1.83041400	-2.55771400	0.76259400
C	-5.05562300	-0.54552800	-0.00164000
H	-3.86597700	0.49543100	-1.47694300
C	-5.07770000	-1.54206300	0.98235500
H	-3.91488700	-3.03885500	2.00969900
H	-5.99259300	-1.75704200	1.52661400
C	-0.40975200	0.92024900	-0.31049800
C	-0.18967700	1.85884500	-1.30847000
C	-0.85367800	1.24724800	0.95793900
C	-0.43431100	3.20025300	-1.01165000
H	0.13788700	1.56559600	-2.29865800
C	-1.09113100	2.59804700	1.23907700
H	-1.04708200	0.48825000	1.70742300
C	-0.87997000	3.57467400	0.26163900
H	-0.28703100	3.96672100	-1.76569500
H	-1.44417900	2.87940800	2.22923300
C	2.77445100	-0.68059200	0.07945600
C	3.69490600	-0.22646400	-0.87168500
C	3.14863400	-0.82106700	1.42056000
C	4.99825300	0.07986200	-0.47529300
H	3.40242000	-0.11513700	-1.91112600
C	4.45502800	-0.51337000	1.80546700
H	2.43187400	-1.16910400	2.15778000
C	5.37925500	-0.06345700	0.86037300
H	5.71464600	0.43028700	-1.21277700
H	4.74822600	-0.62519400	2.84539900
H	-5.95965700	0.01797700	-0.22003700
O	-1.08868000	4.90857500	0.48688800
H	-1.42137300	5.02943500	1.38534300
H	6.39403200	0.17566100	1.16423300

- INT-Me

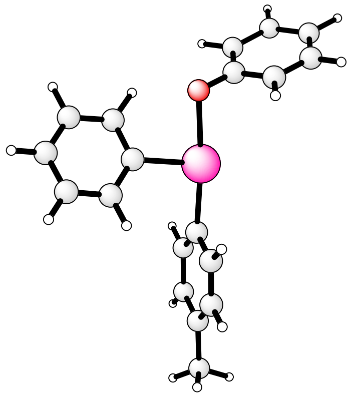

B3LYP/BS2 energy: -821.016487779 a.u.
ZPE: 0.301661 a.u.
Thermal correction to Gibbs Free Energy: 0.247254 a.u.
Solvation energy: -6.56 kcal/mol
Dispersion correction: -55.44 kcal/mol

I	-0.23927500	-0.18294600	-0.24973400
O	-2.36020500	0.72977000	-0.40850400
C	-3.27667600	-0.20458100	-0.14118500
C	-3.54774900	-1.25101900	-1.05389100
C	-4.01057400	-0.18013700	1.06812200
C	-4.51704600	-2.21484400	-0.77279800
H	-3.01111100	-1.26528100	-1.99931000
C	-4.98183700	-1.14087700	1.33467600
H	-3.80564100	0.61851100	1.77612200
C	-5.24140000	-2.16806700	0.41987000
H	-4.71297200	-3.00340600	-1.49568300
H	-5.99817800	-2.91694300	0.63445800
C	0.36963000	1.89399600	-0.10915700
C	1.67069400	2.25050700	-0.44942900
C	-0.57703100	2.81860200	0.32105800
C	2.03691100	3.59535200	-0.34082500
H	2.39015500	1.51388500	-0.78630100
C	-0.18556300	4.15685700	0.42324700
H	-1.58838900	2.49302300	0.53218200
C	1.11432500	4.54622300	0.09736500
H	3.04796500	3.89186600	-0.60470400
H	-0.90957100	4.89362100	0.75895200
C	1.81779800	-0.86046000	-0.01381400
C	2.52291700	-1.34021400	-1.12169600
C	2.39833200	-0.92808300	1.25748200
C	3.80368300	-1.87341000	-0.95600300
H	2.08430500	-1.29583800	-2.11517400
C	3.67842200	-1.45938000	1.41043400
H	1.85928000	-0.56359600	2.12763000
C	4.40048800	-1.94326900	0.30915200
H	4.34630400	-2.23853100	-1.82468800
H	4.12348500	-1.50220300	2.40174700
H	1.40706300	5.58876600	0.17945900
H	-5.54092600	-1.09273200	2.26631900
C	5.77161400	-2.54977500	0.48976500
H	6.36502600	-2.47889100	-0.42621200
H	6.32546300	-2.05592700	1.29385400
H	5.69932100	-3.61287700	0.75105600

- TS-Me-Ph

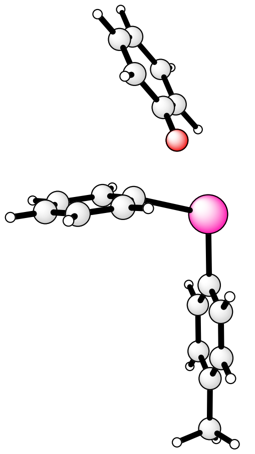

B3LYP/BS2 energy: -820.989811644 a.u.
ZPE: 0.300003 a.u.

Thermal correction to Gibbs Free Energy: 0.246535 a.u.
Solvation energy: -6.41 kcal/mol
Dispersion correction: -55.66 kcal/mol

I	0.35466500	-0.94148200	-0.63536700
O	-2.00714700	-0.32991900	-1.08884900
C	-3.09824400	-0.59558300	-0.38511400
C	-3.14146200	-1.61901900	0.59419800
C	-4.27337100	0.17113500	-0.58894700
C	-4.30816800	-1.87095100	1.31438100
H	-2.25475800	-2.22559500	0.75830800
C	-5.42773000	-0.08442100	0.14165100
H	-4.24222200	0.95856800	-1.33576900
C	-5.45757300	-1.10737100	1.09794700
H	-4.31952800	-2.67049500	2.05141300
H	-6.36406200	-1.30618800	1.66206800
C	-0.68991600	1.15313500	-0.21755100
C	-0.45412100	2.15147800	-1.15135900
C	-1.05662100	1.39437500	1.09718100
C	-0.61264300	3.47601100	-0.72954300
H	-0.18298400	1.91799200	-2.17391100
C	-1.20510800	2.72986400	1.48890900
H	-1.25745300	0.58338100	1.78806100
C	-0.98050900	3.76987000	0.58535800
H	-0.44755500	4.27709600	-1.44512700
H	-1.49948700	2.94552100	2.51247200
C	2.40082900	-0.61379500	-0.11330700
C	3.31076800	-0.17953500	-1.08284100
C	2.82733400	-0.82446900	1.20194900
C	4.64333800	0.03451500	-0.73108300
H	2.98897200	-0.01466900	-2.10651500
C	4.16357000	-0.60606500	1.53692000
H	2.12799400	-1.16149200	1.96080000
C	5.09176100	-0.17112100	0.58100400
H	5.34632300	0.36583300	-1.49146100
H	4.48941100	-0.77808700	2.55981200
H	-1.09369300	4.80180100	0.90232400
H	-6.31696600	0.51609800	-0.03475200
C	6.52902100	0.09435000	0.95954900
H	7.20614200	-0.09916500	0.12238400
H	6.66999000	1.14104700	1.25687300
H	6.84289600	-0.52815300	1.80243000

- TS-Me-Ar

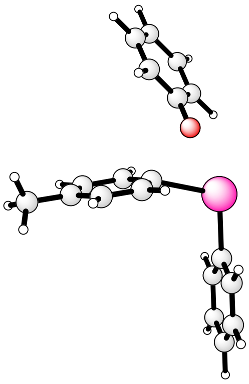

B3LYP/BS2 energy: -820.988529752 a.u.
ZPE: 0.29993 a.u.

Thermal correction to Gibbs Free Energy: 0.246077 a.u.
Solvation energy: -6.36 kcal/mol
Dispersion correction: -55.66 kcal/mol

I	0.77415200	-1.15472400	-0.51495500
O	-1.58836300	-0.72273500	-1.12805100
C	-2.68724300	-1.01238000	-0.44556900
C	-2.72016400	-2.02080500	0.54932200
C	-3.88214100	-0.28994800	-0.69149100
C	-3.89551700	-2.30137700	1.24417000
H	-1.81845000	-2.59538100	0.74415000
C	-5.04483700	-0.57228100	0.01592200
H	-3.85973500	0.48508600	-1.45146300
C	-5.06414000	-1.58025800	0.98800200
H	-3.89839600	-3.08969200	1.99326900
H	-5.97697500	-1.80055100	1.53358000
C	-0.40777600	0.90308000	-0.31460500
C	-0.19046400	1.83588000	-1.31800300
C	-0.84368500	1.23453800	0.95590400
C	-0.44277900	3.17616100	-1.01624800
H	0.13800300	1.53945900	-2.30693800
C	-1.08381100	2.58842400	1.22169300
H	-1.02488500	0.47950800	1.71234400
C	-0.88758800	3.57779700	0.25213700
H	-0.28987500	3.92071100	-1.79452900
H	-1.43122400	2.86767000	2.21361800
C	2.77486000	-0.68029500	0.08229100
C	3.69793600	-0.23816200	-0.87188200
C	3.14567000	-0.80573500	1.42573800
C	5.00077600	0.07115700	-0.47607400
H	3.40793000	-0.13847500	-1.91322800
C	4.45149700	-0.49493500	1.81020600
H	2.42670300	-1.14414300	2.16528400
C	5.37842300	-0.05713900	0.86207500
H	5.71944300	0.41199500	-1.21585900
H	4.74207800	-0.59485200	2.85207200
H	-5.94899100	-0.00504100	-0.19206200
H	6.39280800	0.18420100	1.16552400
C	-1.16334300	5.03358900	0.54915100
H	-2.06494800	5.38329900	0.03149900
H	-1.31417000	5.20018600	1.61937800
H	-0.33648800	5.67439500	0.22397700

- INT-F

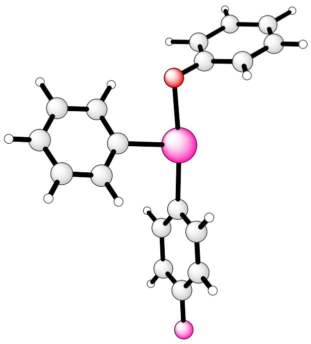

B3LYP/BS2 energy: -880.956546135 a.u.
ZPE: 0.266089 a.u.
Thermal correction to Gibbs Free Energy: 0.213426 a.u.

Solvation energy: -6.49 kcal/mol
Dispersion correction: -52.29 kcal/mol

I	-0.23318500	-0.18605300	-0.22485500
O	-2.31956600	0.76971900	-0.38099900
C	-3.26001100	-0.14935600	-0.13517600
C	-3.55499600	-1.16669800	-1.07197700
C	-3.99348000	-0.13283000	1.07367800
C	-4.54923600	-2.11138400	-0.81404800
H	-3.01664600	-1.17325100	-2.01645100
C	-4.98969600	-1.07410600	1.31733300
H	-3.76925100	0.64436400	1.79941000
C	-5.27382400	-2.07278100	0.37878700
H	-4.76432000	-2.87792400	-1.55480100
H	-6.04998200	-2.80655600	0.57534600
C	0.42392200	1.87681700	-0.11315500
C	1.72032500	2.20475500	-0.49687100
C	-0.48928100	2.82282400	0.34219200
C	2.11808200	3.54191300	-0.40634100
H	2.41273000	1.45199100	-0.85398700
C	-0.06683200	4.15291000	0.42568600
H	-1.50059300	2.52157900	0.58597600
C	1.22990500	4.51345400	0.05689900
H	3.12592000	3.81610500	-0.70420000
H	-0.76419000	4.90605000	0.78076100
C	1.81631900	-0.90143100	0.00298000
C	2.48972300	-1.43409300	-1.10252700
C	2.41351700	-0.94427500	1.26908500
C	3.75921700	-1.99765100	-0.95216400
H	2.03294700	-1.41177300	-2.08815900
C	3.68216000	-1.50079000	1.43127600
H	1.89476500	-0.54234100	2.13464400
C	4.33210100	-2.01856900	0.31443600
H	4.30161000	-2.41506600	-1.79375000
H	4.16664500	-1.54404700	2.40094300
H	1.54677900	5.54986600	0.12506200
H	-5.54888500	-1.03295400	2.24913600
F	5.55559000	-2.55947000	0.46720300

- TS-F-Ph

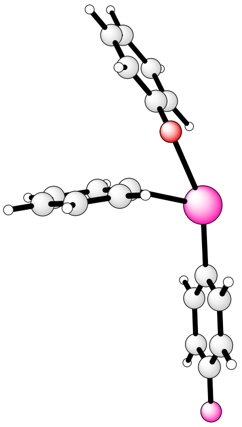

B3LYP/BS2 energy: -880.929611399 a.u.
ZPE: 0.264458 a.u.
Thermal correction to Gibbs Free Energy: 0.212876 a.u.
Solvation energy: -6.33 kcal/mol
Dispersion correction: -52.50 kcal/mol

I	0.37111800	-0.92566200	-0.62892300
O	-1.97668500	-0.32359200	-1.09249800
C	-3.07055300	-0.60538700	-0.39775700
C	-3.10886300	-1.63011500	0.57989300
C	-4.25306900	0.14628500	-0.61259600
C	-4.27886000	-1.89759100	1.28916100
H	-2.21647200	-2.22603200	0.75181600
C	-5.41056600	-0.12463700	0.10728400
H	-4.22518800	0.93429700	-1.35887600
C	-5.43570800	-1.14854200	1.06268900
H	-4.28675300	-2.69790200	2.02529300
H	-6.34462100	-1.35940800	1.61836700
C	-0.67573700	1.17039000	-0.20627600
C	-0.43754600	2.17173500	-1.13558400
C	-1.05225300	1.40342700	1.10668800
C	-0.60350300	3.49414500	-0.70930100
H	-0.15972400	1.94305200	-2.15741700
C	-1.20786900	2.73705200	1.50253500
H	-1.25516900	0.58888000	1.79274700
C	-0.98080000	3.78113500	0.60437000
H	-0.43675100	4.29864500	-1.42057100
H	-1.50973900	2.94773200	2.52489800
C	2.41821400	-0.60485200	-0.10010700
C	3.33097700	-0.16944900	-1.06878000
C	2.83793400	-0.81467500	1.21936100
C	4.66487200	0.04841000	-0.72383000
H	3.01001200	-0.00166600	-2.09190300
C	4.17023400	-0.59916200	1.57121600
H	2.13317800	-1.14716500	1.97472300
C	5.06041800	-0.17080000	0.59171700
H	5.39311700	0.38266500	-1.45483100
H	4.52251200	-0.75684900	2.58484800
H	-1.09956800	4.81135000	0.92467700
H	-6.30583200	0.46430400	-0.07699600
F	6.34681000	0.03807700	0.92800600

- TS-F-Ar

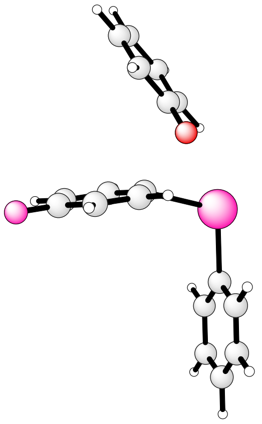

B3LYP/BS2 energy: -880.928298169 a.u.
ZPE: 0.264395 a.u.
Thermal correction to Gibbs Free Energy: 0.212419 a.u.
Solvation energy: -6.26 kcal/mol
Dispersion correction: -52.56 kcal/mol

I	0.76026400	-1.15627500	-0.51679700

O	-1.59885900	-0.68797700	-1.12240800
C	-2.70625700	-0.95942400	-0.44560100
C	-2.75222800	-1.94570400	0.57077000
C	-3.89492700	-0.23669700	-0.71848900
C	-3.93495400	-2.20512600	1.26132400
H	-1.85533800	-2.52066200	0.78564700
C	-5.06498300	-0.49751700	-0.01514100
H	-3.86217500	0.52098700	-1.49537100
C	-5.09728600	-1.48377100	0.97873200
H	-3.94867700	-2.97667800	2.02743100
H	-6.01591000	-1.68743000	1.52093300
C	-0.39385500	0.90861900	-0.30714600
C	-0.16354900	1.84721200	-1.30407200
C	-0.83193700	1.23773600	0.96727800
C	-0.39682200	3.19260600	-1.00458500
H	0.16144700	1.55195100	-2.29438400
C	-1.05934100	2.58759300	1.25497800
H	-1.02867200	0.47618000	1.71322900
C	-0.83449700	3.54115400	0.26896800
H	-0.24456100	3.96408400	-1.75208300
H	-1.40800900	2.89630700	2.23482600
C	2.76168000	-0.68938900	0.07816800
C	3.68607300	-0.25466600	-0.87812700
C	3.13098600	-0.81122500	1.42236500
C	4.98979100	0.05153300	-0.48293500
H	3.39690900	-0.15840600	-1.91999100
C	4.43777100	-0.50337400	1.80571600
H	2.41078300	-1.14438300	2.16303600
C	5.36630000	-0.07256500	0.85593100
H	5.70988900	0.38684000	-1.22375900
H	4.72773700	-0.59994500	2.84800300
H	-5.96441500	0.06924000	-0.24325000
F	-1.04613800	4.84440000	0.55529700
H	6.38130200	0.16673500	1.15875200

- INT-Cl

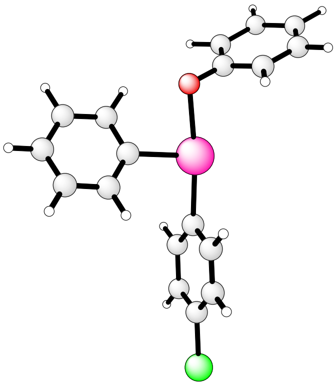

B3LYP/BS2 energy: -1241.31010348 a.u.
ZPE: 0.264638 a.u.
Thermal correction to Gibbs Free Energy: 0.211245 a.u.
Solvation energy: -6.57 kcal/mol
Dispersion correction: -54.46 kcal/mol

I	-0.48027400	-0.14324000	-0.24663200
O	-2.65676800	0.56407600	-0.35938500
C	-3.49171600	-0.45184500	-0.10812200
C	-3.67812300	-1.49755500	-1.04122500
C	-4.21937100	-0.50939500	1.10253500
C	-4.56344800	-2.54337900	-0.77654800
H	-3.14609900	-1.44927000	-1.98809800
C	-5.10767400	-1.55156600	1.35257000
H	-4.07850900	0.28992400	1.82514000
C	-5.28447600	-2.57872900	0.41848300
H	-4.69669600	-3.33139800	-1.51397200
H	-5.97582600	-3.39165200	0.62007600
C	-0.07404600	1.98092300	-0.11622500
C	1.15630800	2.47089100	-0.54351800
C	-1.07584100	2.80099600	0.39344400
C	1.39106700	3.84528500	-0.44217200
H	1.92145100	1.81473000	-0.94103100
C	-0.81602000	4.17137800	0.48684600
H	-2.03222400	2.37447000	0.66873700
C	0.41066600	4.69349100	0.07436200
H	2.34465200	4.24568800	-0.77374900
H	-1.58483200	4.82794000	0.88353100
C	1.65036300	-0.59677700	-0.05209800
C	2.35970500	-1.06741800	-1.16161400
C	2.27936100	-0.53115700	1.19634200
C	3.69448400	-1.46109800	-1.03323300
H	1.88226600	-1.12890200	-2.13590600
C	3.61267300	-0.91729000	1.33567700
H	1.73726100	-0.17556200	2.06803200
C	4.30758900	-1.37886600	0.21620500
H	4.25107000	-1.82435200	-1.89030200
H	4.10747500	-0.86627400	2.29952200
H	0.60117900	5.75986100	0.14996100
H	-5.66545400	-1.56743400	2.28591900
Cl	5.98435000	-1.86858800	0.38696900

- TS-Cl-Ph

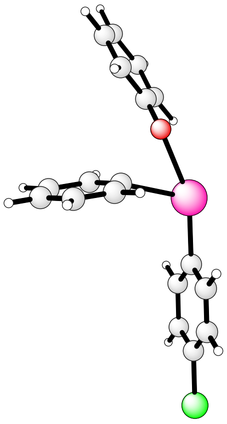

B3LYP/BS2 energy: -1241.28318913 a.u.
ZPE: 0.262975 a.u.
Thermal correction to Gibbs Free Energy: 0.21049 a.u.
Solvation energy: -6.40 kcal/mol
Dispersion correction: -54.62 kcal/mol

I	0.05468900	-0.92984500	-0.67467000
O	-2.29906100	-0.32239300	-1.06656300
C	-3.37117500	-0.60091600	-0.33753600
C	-3.37738200	-1.61501400	0.65188900
C	-4.56223800	0.14409700	-0.52702600
C	-4.52578800	-1.87918600	1.39677700

H	-2.47807600	-2.20559500	0.80496400
C	-5.69778700	-0.12353500	0.22801900
H	-4.55850500	0.92393900	-1.28232500
C	-5.69162400	-1.13717200	1.19473700
H	-4.50965900	-2.67135900	2.14147700
H	-6.58366500	-1.34553300	1.77798000
C	-0.96423600	1.17677800	-0.22584100
C	-0.74540200	2.17309400	-1.16461500
C	-1.30663100	1.41378200	1.09530200
C	-0.89489400	3.49756000	-0.73789000
H	-0.49411500	1.94015700	-2.19231500
C	-1.44633800	2.74925300	1.49100800
H	-1.49689500	0.60174400	1.78796900
C	-1.23756300	3.78960500	0.58402900
H	-0.74257100	4.29934000	-1.45539300
H	-1.72172500	2.96422100	2.51990200
C	2.12120800	-0.62115400	-0.20970700
C	3.00897000	-0.20267900	-1.20705400
C	2.57883500	-0.82290600	1.09718100
C	4.35461700	0.00565500	-0.90298500
H	2.66168100	-0.04019100	-2.22252100
C	3.92320000	-0.61656500	1.40736100
H	1.89531200	-1.14226500	1.87746000
C	4.79908300	-0.20409000	0.40270200
H	5.05055100	0.32700400	-1.67004300
H	4.28744600	-0.77329500	2.41670200
H	-1.34373900	4.82122500	0.90417600
H	-6.60038000	0.45977300	0.06273600
Cl	6.49054000	0.05541900	0.78830300

- TS-Cl-Ar

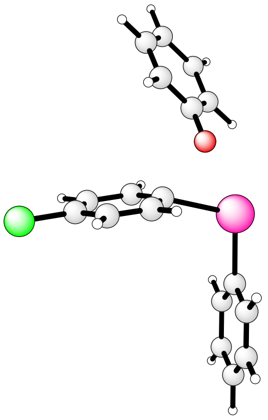

B3LYP/BS2 energy: -1241.28332339 a.u.
ZPE: 0.263031 a.u.
Thermal correction to Gibbs Free Energy: 0.210428 a.u.
Solvation energy: -6.49 kcal/mol
Dispersion correction: -54.90 kcal/mol

I	0.89663500	-1.35906700	-0.47654200
O	-1.47788900	-1.07493200	-1.12120500
C	-2.56826600	-1.38501400	-0.43042500
C	-2.56597000	-2.37221900	0.58513800
C	-3.78440600	-0.70528500	-0.68935600
C	-3.73042500	-2.67458900	1.28921100
H	-1.64692800	-2.91595700	0.78769800
C	-4.93576900	-1.00737800	0.02871200
H	-3.78885700	0.05281200	-1.46643100
C	-4.92088600	-1.99450600	1.02176400
H	-3.70737200	-3.44689700	2.05426800
H	-5.82488400	-2.23068200	1.57519500
C	-0.37987500	0.61563000	-0.37845800
C	-0.19931100	1.52213000	-1.41574300
C	-0.84937300	0.98258400	0.87522700
C	-0.52017200	2.86184800	-1.18383200
H	0.15463500	1.20400500	-2.38902000
C	-1.16471700	2.32690200	1.09425500
H	-1.00394600	0.24992200	1.65911500
C	-0.99353300	3.25518800	0.06841300
H	-0.40343000	3.59149300	-1.97798800
H	-1.54048100	2.64310600	2.06141700
C	2.84964000	-0.71131500	0.11023000
C	3.76910900	-0.31316300	-0.86637600
C	3.18893200	-0.67569800	1.46714100
C	5.03964500	0.11551300	-0.47712000
H	3.50216100	-0.33828400	-1.91827700
C	4.46217900	-0.24451000	1.84427100
H	2.47153400	-0.98115500	2.22226900
C	5.38650800	0.15040200	0.87496500
H	5.75676800	0.42265100	-1.23284100
H	4.72932700	-0.21751400	2.89668200
H	-5.85717600	-0.47238000	-0.18782100
H	6.37544900	0.48562700	1.17286900
Cl	-1.37736400	4.94730100	0.35653900

- INT-CF_3

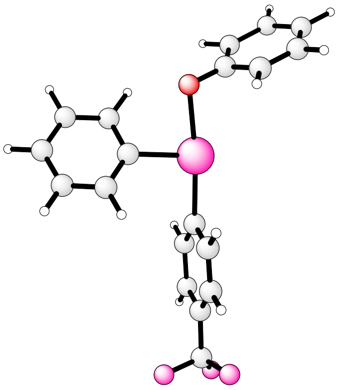

B3LYP/BS2 energy: -1118.84177128 a.u.
ZPE: 0.278993 a.u.
Thermal correction to Gibbs Free Energy: 0.22168 a.u.
Solvation energy: -6.62 kcal/mol
Dispersion correction: -56.32 kcal/mol

I	-0.95598800	-0.09112500	-0.25836600
O	-3.18876600	0.40116500	-0.32466200
C	-3.90973600	-0.70099300	-0.08046400
C	-3.98636600	-1.75288000	-1.02174200
C	-4.62282600	-0.84497700	1.13151000
C	-4.75291300	-2.89008500	-0.76281400
H	-3.46695400	-1.63998300	-1.97008700
C	-5.39312000	-1.97841200	1.37548200
H	-4.56685800	-0.04043000	1.85978800
C	-5.46144800	-3.01168900	0.43390500
H	-4.80338800	-3.68202200	-1.50622200

H	-6.06080400	-3.89559400	0.63103100
C	-0.74985900	2.05932700	-0.11763000
C	0.40772600	2.67091400	-0.58912700
C	-1.80591700	2.77331600	0.43998700
C	0.51139100	4.06077400	-0.48051800
H	1.21522500	2.09698400	-1.02767300
C	-1.67767000	4.16180600	0.53909700
H	-2.70536000	2.25410900	0.74628700
C	-0.52517700	4.80455900	0.08496400
H	1.40669100	4.55532600	-0.84567800
H	-2.49033400	4.73691900	0.97300600
C	1.21715000	-0.33534700	-0.10130800
C	1.93762400	-0.77376100	-1.21647800
C	1.86292200	-0.17721300	1.13029700
C	3.30396200	-1.04169000	-1.10592000
H	1.44245900	-0.90865400	-2.17420100
C	3.22713200	-0.44085500	1.24079700
H	1.30773000	0.15033800	2.00449600
C	3.94651400	-0.87274100	0.12188400
H	3.86562300	-1.38644600	-1.96763400
H	3.73121000	-0.32559500	2.19466500
H	-0.43664100	5.88380200	0.16555600
H	-5.94302800	-2.06106500	2.30993800
C	5.43046200	-1.10565100	0.23500400
F	6.12686400	0.03608400	0.02847400
F	5.77318000	-1.55839000	1.46047500
F	5.86915600	-2.00478300	-0.67163100

- TS-CF_3-Ph

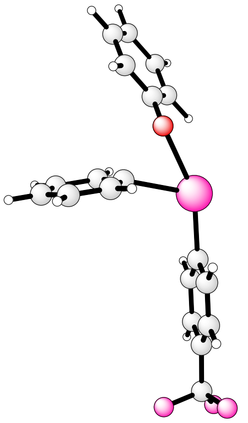

B3LYP/BS2 energy: -1118.81491378 a.u.
ZPE: 0.277357 a.u.
Thermal correction to Gibbs Free Energy: 0.220511 a.u.
Solvation energy: -6.42 kcal/mol
Dispersion correction: -56.48 kcal/mol

I	-0.48555400	-0.95178500	-0.72113300
O	-2.83867300	-0.32329800	-1.04082500
C	-3.88824000	-0.58648700	-0.27431700
C	-3.87048000	-1.59249400	0.72318000
C	-5.07812400	0.16797300	-0.43093500
C	-4.99608300	-1.84031500	1.50741200
H	-2.97198100	-2.19021800	0.85116100
C	-6.19041700	-0.08332300	0.36318000
H	-5.09218300	0.94164600	-1.19239400
C	-6.16128700	-1.08936600	1.33744800

H	-4.96253600	-2.62663700	2.25765700
H	-7.03546600	-1.28498400	1.95129700
C	-1.45809500	1.17426200	-0.25717600
C	-1.25303400	2.15874400	-1.21082500
C	-1.76183700	1.42491200	1.07050500
C	-1.37473500	3.48852200	-0.79120700
H	-1.03230600	1.91423700	-2.24280300
C	-1.87429900	2.76528300	1.45840900
H	-1.94422000	0.62068700	1.77436300
C	-1.67754000	3.79532000	0.53700600
H	-1.23217700	4.28246200	-1.51930900
H	-2.11931400	2.99212500	2.49238300
C	1.60118700	-0.66055700	-0.32330900
C	2.46122700	-0.27101200	-1.35595000
C	2.09270900	-0.84818900	0.97341500
C	3.81580200	-0.07419400	-1.08996900
H	2.08386200	-0.12661800	-2.36319300
C	3.44767600	-0.65048600	1.23457000
H	1.42752400	-1.15065800	1.77569600
C	4.30805400	-0.26341100	0.20373600
H	4.49046800	0.21777700	-1.88758900
H	3.83735800	-0.80400100	2.23527200
H	-1.76230200	4.83076500	0.85106600
H	-7.09277100	0.50679600	0.22287400
C	5.75950500	0.00708000	0.50216300
F	5.95482800	1.29132700	0.87912900
F	6.21797000	-0.77205000	1.50562400
F	6.54251800	-0.21104800	-0.57628800

- TS-CF_3-Ar

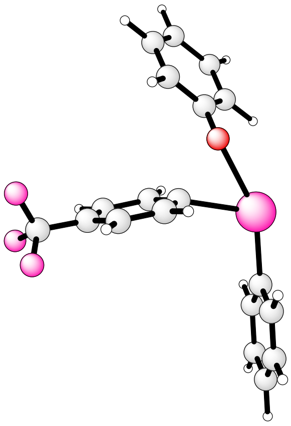

B3LYP/BS2 energy: -1118.81775636 a.u.
ZPE: 0.277361 a.u.
Thermal correction to Gibbs Free Energy: 0.220302 a.u.
Solvation energy: -6.57 kcal/mol
Dispersion correction: -56.90 kcal/mol

I	1.52398200	-1.33451300	-0.46129900
O	-0.80008000	-1.84444400	-1.12464800
C	-1.73660300	-2.48069700	-0.42850300
C	-1.41971700	-3.41624200	0.58558500
C	-3.10491000	-2.21725300	-0.68267500
C	-2.42719000	-4.07024300	1.29300400
H	-0.37554000	-3.64243500	0.78517900
C	-4.10032700	-2.86686200	0.03877200
H	-3.35087700	-1.49938200	-1.45901800

C	-3.77206700	-3.79946700	1.03005600
H	-2.15937400	-4.79601900	2.05703100
H	-4.55380900	-4.30835300	1.58606500
C	-0.30930800	0.10919600	-0.40790100
C	-0.41256800	1.01389300	-1.46121100
C	-0.88892500	0.32544700	0.83654200
C	-1.15304500	2.17559100	-1.25332200
H	0.04728800	0.81430500	-2.42144000
C	-1.62657500	1.49663000	1.02015400
H	-0.80236200	-0.40221400	1.63507400
C	-1.75534600	2.42544300	-0.01456200
H	-1.25460200	2.89384600	-2.06093000
H	-2.09280100	1.68723900	1.98084300
C	3.14857000	-0.06889300	0.12136000
C	3.89289200	0.59514100	-0.85976000
C	3.44446500	0.09762800	1.47852800
C	4.94645900	1.42633000	-0.47420800
H	3.65862800	0.46622900	-1.91183000
C	4.50006400	0.93177200	1.85168400
H	2.86185500	-0.41623200	2.23660200
C	5.24991700	1.59514800	0.87823800
H	5.52819500	1.94162000	-1.23295500
H	4.73426700	1.06221200	2.90416000
H	-5.14395000	-2.64873400	-0.17398600
H	6.06951400	2.24344500	1.17328200
C	-2.57350400	3.67054000	0.16850400
F	-1.98310600	4.74527700	-0.40680300
F	-2.76772600	3.96509200	1.47321200
F	-3.79922900	3.55969800	-0.39682800

- INT-NO_2

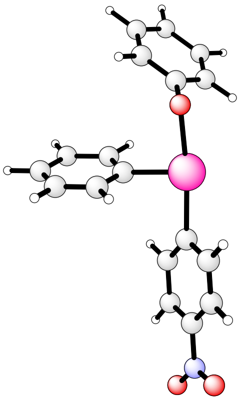

B3LYP/BS2 energy: -986.250813874 a.u.
ZPE: 0.27668 a.u.
Thermal correction to Gibbs Free Energy: 0.221151 a.u.
Solvation energy: -9.18 kcal/mol
Dispersion correction: -57.24 kcal/mol

I	-0.37359100	-0.85851600	-0.65246600
O	-2.52737800	-0.97035600	-1.21773400
C	-3.49375600	-0.92717200	-0.28602400
C	-3.57911300	-1.87224800	0.76002300
C	-4.48458700	0.07604300	-0.35516200
C	-4.60901900	-1.80670600	1.69766300
H	-2.84007900	-2.66798800	0.81227700
C	-5.51701100	0.12525300	0.57979800

H	-4.42441600	0.79867600	-1.16344100
C	-5.58520800	-0.80938900	1.61698500
H	-4.65608200	-2.54825000	2.49159100
H	-6.38918500	-0.76667200	2.34575800
C	-0.72759900	1.22473700	-0.36945800
C	-0.20975500	2.12802700	-1.29726300
C	-1.50370200	1.63112800	0.71553200
C	-0.46828300	3.48802500	-1.11686600
H	0.38444900	1.78832700	-2.13830600
C	-1.75251800	2.99800300	0.87484400
H	-1.91567200	0.91271500	1.41353000
C	-1.23657300	3.92198400	-0.03397400
H	-0.07032600	4.20480400	-1.82885500
H	-2.35486800	3.33176900	1.71415100
C	1.76011300	-0.51510500	-0.17540900
C	2.71048900	-1.14109500	-0.98946200
C	2.17081500	0.22271700	0.94075100
C	4.07062000	-1.02942800	-0.70010000
H	2.40164800	-1.71331700	-1.86096500
C	3.52602500	0.34600800	1.23993600
H	1.43940000	0.71272100	1.57643800
C	4.45419600	-0.28377800	0.41153300
H	4.82814400	-1.49749600	-1.31663900
H	3.87387700	0.91257900	2.09500100
H	-1.43496600	4.98112500	0.09873800
H	-6.27364000	0.90223700	0.50007100
N	5.88804100	-0.15449500	0.72123100
O	6.19990100	0.51031700	1.70832300
O	6.68559500	-0.71876600	-0.02650800

- TS-NO_2-Ph

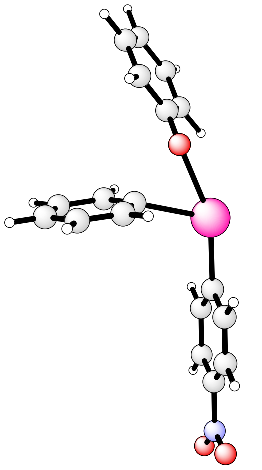

B3LYP/BS2 energy: -986.225899137 a.u.
ZPE: 0.275212 a.u.
Thermal correction to Gibbs Free Energy: 0.220674 a.u.
Solvation energy: -8.10 kcal/mol
Dispersion correction: -56.03 kcal/mol

I	-0.16795800	-0.91669400	-0.70957400
O	-2.50594000	-0.29957500	-1.05810000
C	-3.56914700	-0.59357600	-0.32180900
C	-3.55662300	-1.60920000	0.66597800
C	-4.76955600	0.13822200	-0.50386300
C	-4.69712500	-1.88695600	1.41784900

H	-2.65047500	-2.19079900	0.81232200
C	-5.89674000	-0.14303900	0.25821500
H	-4.77918100	0.91858100	-1.25847700
C	-5.87225000	-1.15762200	1.22387000
H	-4.66760900	-2.67984800	2.16117300
H	-6.75804600	-1.37676900	1.81250200
C	-1.14948800	1.20027200	-0.20971200
C	-0.93252300	2.20672700	-1.13646500
C	-1.48197500	1.41440700	1.11679000
C	-1.07163600	3.52536400	-0.68711400
H	-0.69016800	1.98830100	-2.16941000
C	-1.61103300	2.74449800	1.53444500
H	-1.67326800	0.59202800	1.79687800
C	-1.40276500	3.79737600	0.64183900
H	-0.92032800	4.33764500	-1.39276900
H	-1.87815300	2.94488100	2.56835700
C	1.91301000	-0.63233100	-0.27240000
C	2.78818700	-0.21437900	-1.28374300
C	2.38272300	-0.84524200	1.03048100
C	4.13710300	-0.01623800	-0.99715800
H	2.42482200	-0.04663600	-2.29232100
C	3.73008800	-0.64901800	1.32437400
H	1.70400100	-1.16425300	1.81455500
C	4.58476200	-0.23733800	0.30370200
H	4.84009100	0.30302100	-1.75656400
H	4.12551400	-0.80727500	2.32005800
H	-1.50093800	4.82429000	0.97902400
H	-6.80705500	0.42972900	0.09943200
N	6.00995200	-0.02777600	0.61080100
O	6.37781300	-0.22888900	1.76723600
O	6.74222800	0.33531000	-0.30846000

- TS-NO_2-Ar

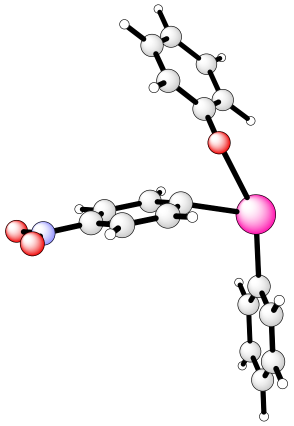

B3LYP/BS2 energy: -986.231432932 a.u.
ZPE: 0.275534 a.u.
Thermal correction to Gibbs Free Energy: 0.221441 a.u.
Solvation energy: -8.70 kcal/mol
Dispersion correction: -56.71 kcal/mol

I	1.05125500	-1.44534300	-0.46796500
O	-1.31971300	-1.33968900	-1.12882200
C	-2.39591800	-1.71101300	-0.43763700
C	-2.34072700	-2.70587500	0.56647200
C	-3.64216400	-1.09021800	-0.69161700
C	-3.48800700	-3.07414700	1.26745800
H	-1.39514100	-3.20424200	0.76359700
C	-4.77699400	-1.45741500	0.02335100
H	-3.68581400	-0.32663200	-1.46196500
C	-4.71103100	-2.45204300	1.00613900
H	-3.42577400	-3.85180600	2.02460700
H	-5.60157400	-2.73903500	1.55717000
C	-0.33555600	0.38468000	-0.40012900
C	-0.22160500	1.29958100	-1.45031100
C	-0.83604900	0.73551800	0.85516100
C	-0.65528600	2.60329000	-1.24009400
H	0.16604800	0.99727900	-2.41552000
C	-1.26622500	2.04383200	1.05277000
H	-0.92460600	0.00258300	1.64842900
C	-1.16611000	2.96537200	0.00924200
H	-0.60467000	3.34410700	-2.02871400
H	-1.67342300	2.36022800	2.00533100
C	2.93209800	-0.61907100	0.13001600
C	3.82485200	-0.16139100	-0.84491400
C	3.24742500	-0.53203800	1.49012500
C	5.04833100	0.38250500	-0.44888800
H	3.57471400	-0.22816600	-1.89904100
C	4.47355800	0.01508900	1.87294700
H	2.55000100	-0.88590000	2.24274700
C	5.37224500	0.47130600	0.90642500
H	5.74599000	0.73743800	-1.20168500
H	4.72393900	0.08414900	2.92741800
H	-5.72412700	-0.96761700	-0.18792700
H	6.32424700	0.89656500	1.20939200
N	-1.60204600	4.34105600	0.22899800
O	-2.04151800	4.63399100	1.34338600
O	-1.50489500	5.13183500	

Computational references

[25] (a) Becke, A. D. *J. Chem. Phys.* **1993**, *98*, 5648-5652. (b) Lee, C.; Yang, W.; Parr, R. G. *Phys. Rev.* **1988**, *B37*, 785-789.
[26] Gaussian 09, Revision A.02, Gaussian, Inc., Wallingford CT, **2009**.
[27] Hay, P. J.; Wadt, W. R. *J. Chem. Phys.* **1985**, *82*, 270-283.
[28] Check, C. E.; Faust, T. O.; Bailey, J. M.; Wright, B. J.; Gilbert, T. M.; Sunderlin, L. S. *J. Phys. Chem. A* **2001**, *105*, 8111-8116.
[29] (a) Klamt, A.; Schüürmann, G. *J. Chem. Soc., Perkin. Trans 2.* **1993**, 799-805. (b) Andzelm, J.; Kölmel, C.; Klamt, A. *J. Chem. Phys.* **1995**, *103*, 9312-9320. (c) Barone, V.; Cossi, M. *J. Phys. Chem. A* **1998**, *102*, 1995-2001. (d) Cossi, M.; Gega, N.; Scalmani, G.; Barone, V. *J. Comput. Chem.* **2003**, *24*, 669-691.
[30] Grimme, S.; Antony, J.; Ehrlich, S.; Krieg, H. *J. Chem. Phys.* **2010**, *132*, 154104.
[31] Grimme, S.; Ehrlich, S.; Goerigk, L. *J. Comput. Chem.* **2011**, *32*, 1456-1465.

8. Copies of ^{1}H and ^{13}C NMR Spectra of Novel Compounds

Start on the next page.

Parameter	Value
1 Solvent	DMSO-d6
2 Temperature	297.0
3 Number of Scans	16
4 Spectrometer Frequency	400.13
5 Nucleus	1H

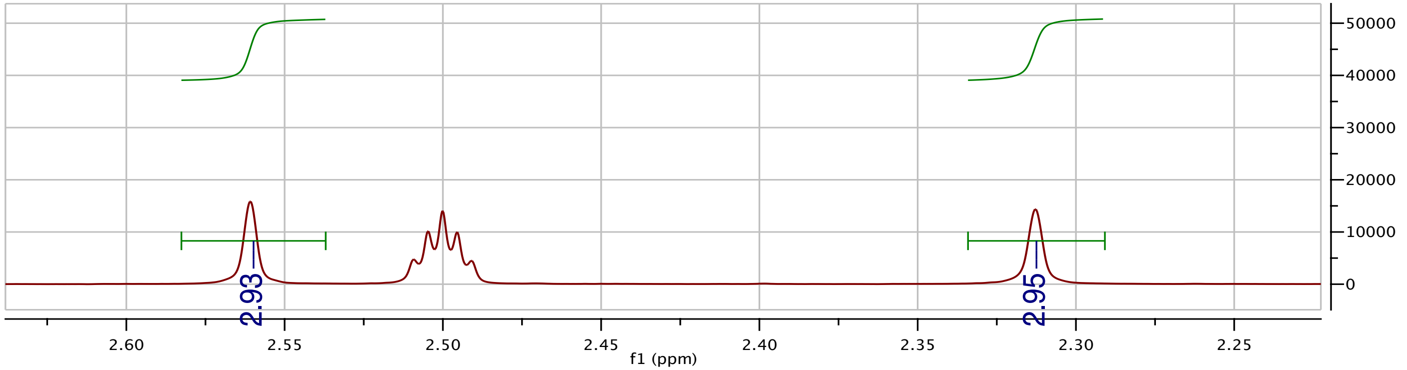

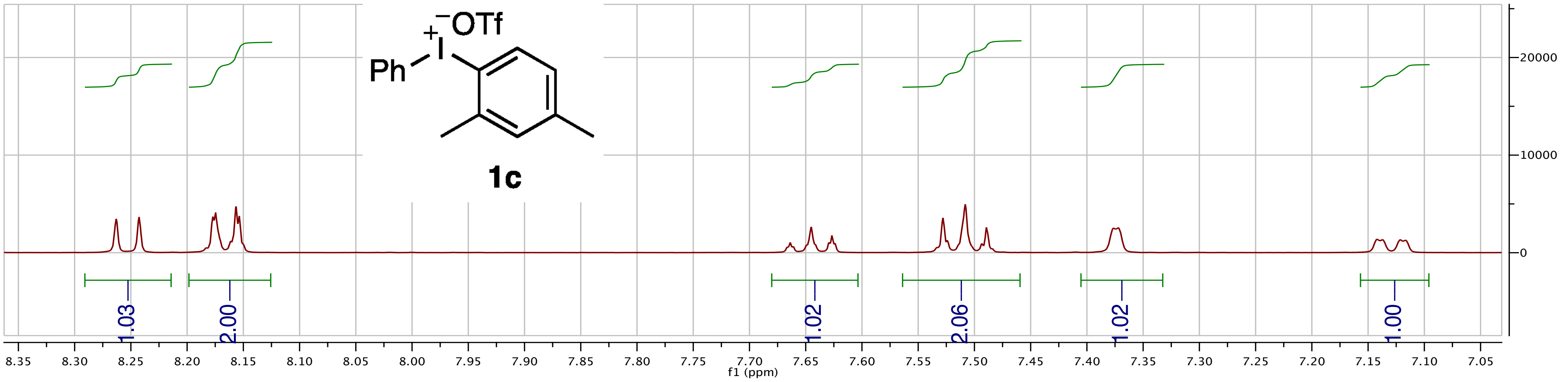

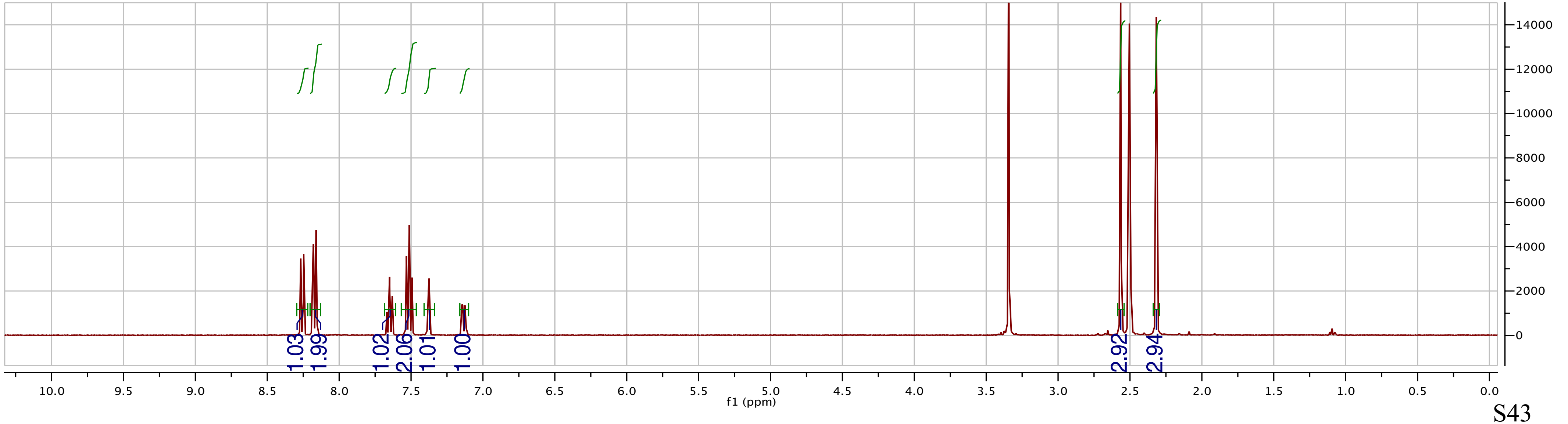

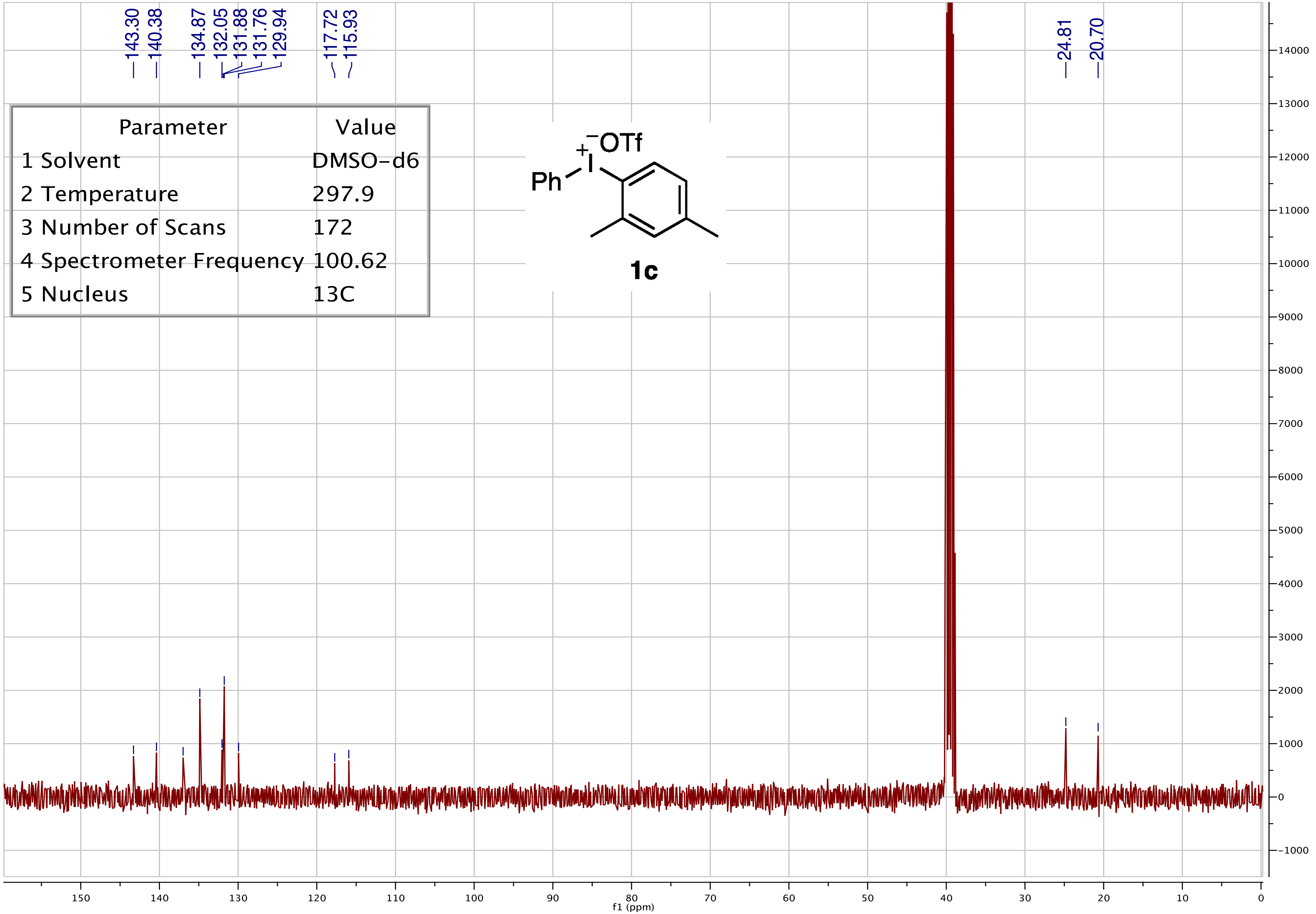

Parameter	Value
1 Solvent	DMSO-d6
2 Temperature	297.9
3 Number of Scans	172
4 Spectrometer Frequency	100.62
5 Nucleus	13C

Parameter	Value
1 Solvent	DMSO-d6
2 Temperature	300.2
3 Number of Scans	16
4 Spectrometer Frequency	400.13
5 Nucleus	1H

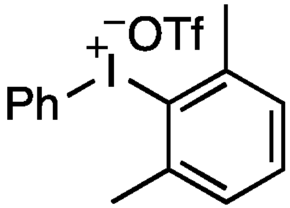

1e

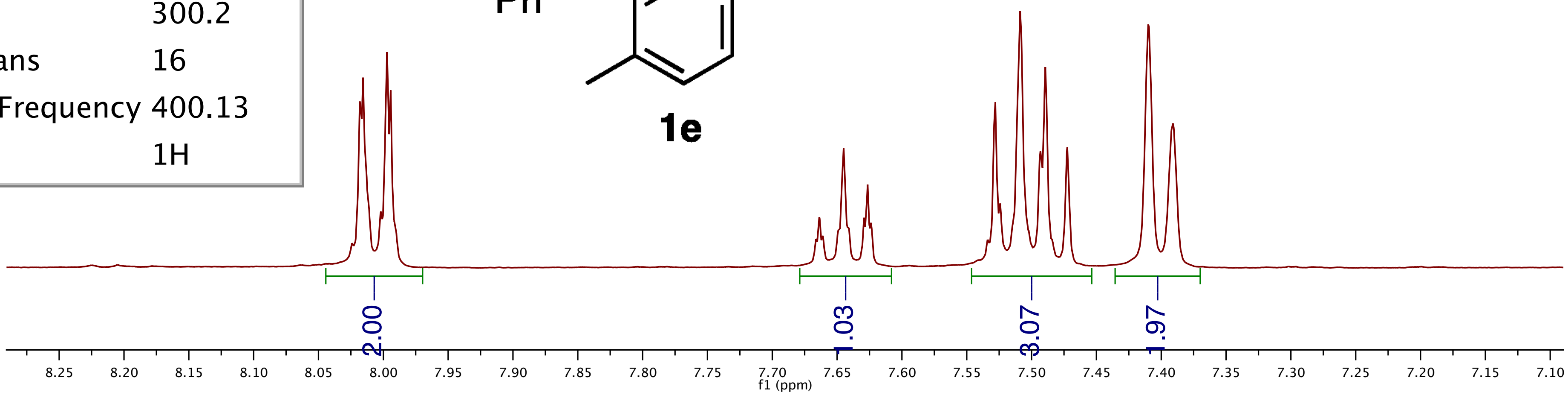

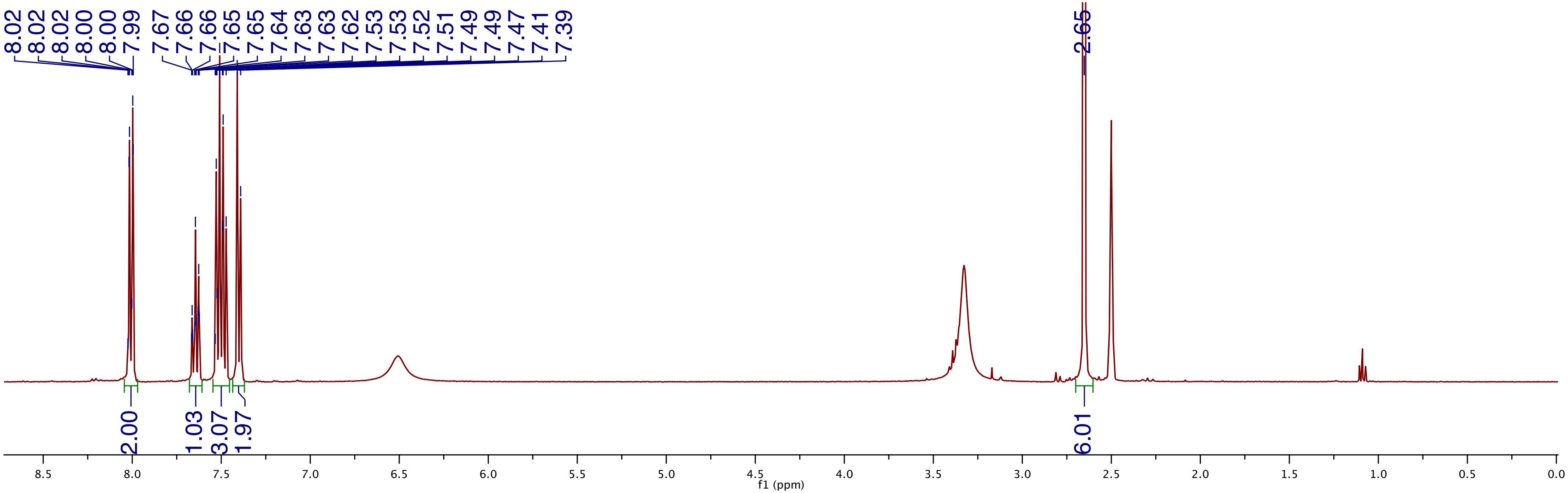

Parameter	Value
1 Solvent	DMSO-d6
2 Temperature	300.2
3 Number of Scans	172
4 Spectrometer Frequency	100.62
5 Nucleus	13C

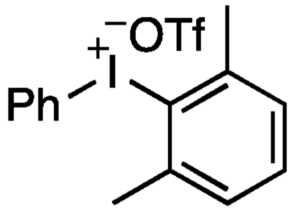

1e

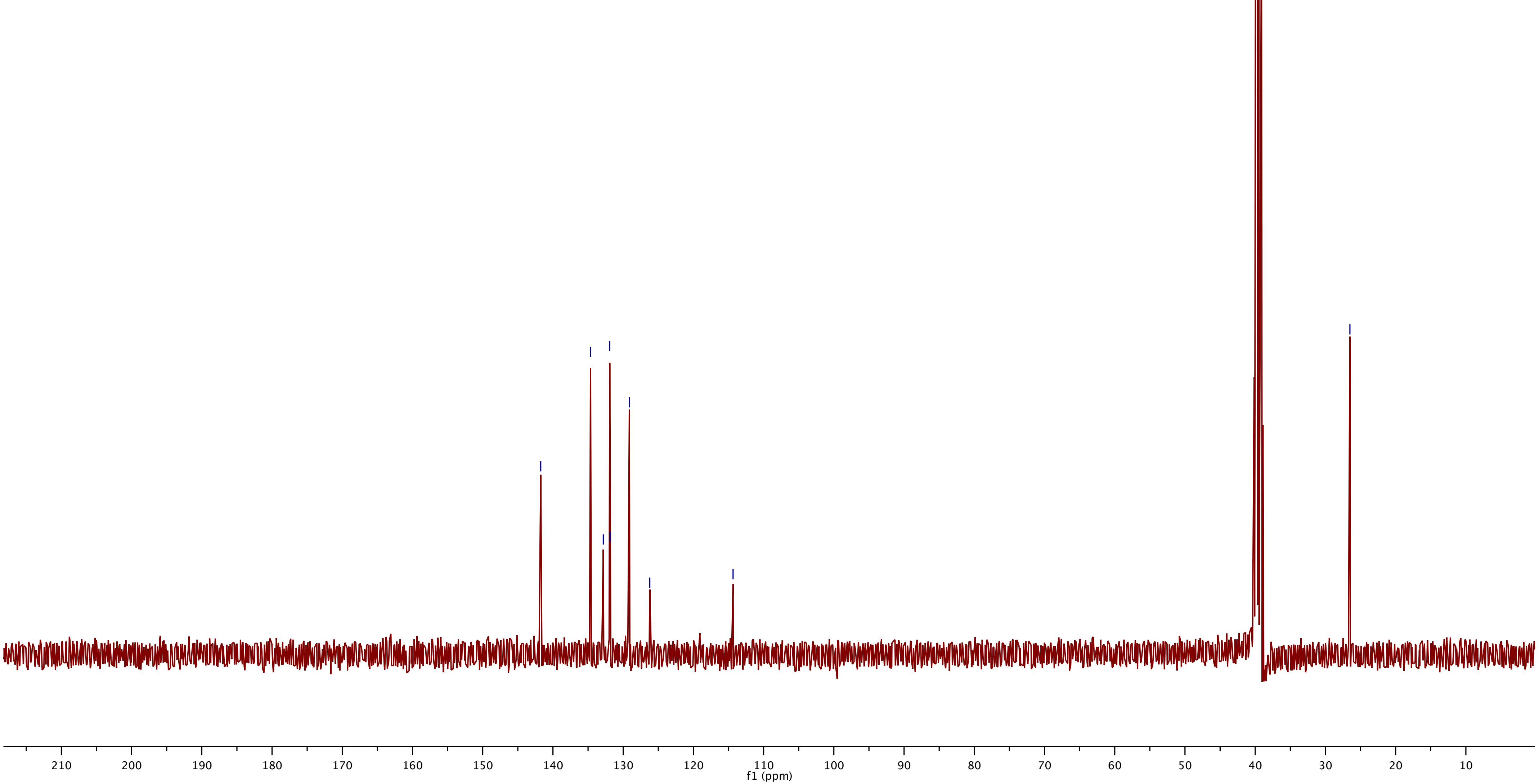

Parameter	Value
1 Solvent	DMSO-d6
2 Temperature	300.2
3 Number of Scans	16
4 Spectrometer Frequency	400.13
5 Nucleus	1H

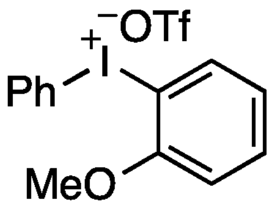

2b

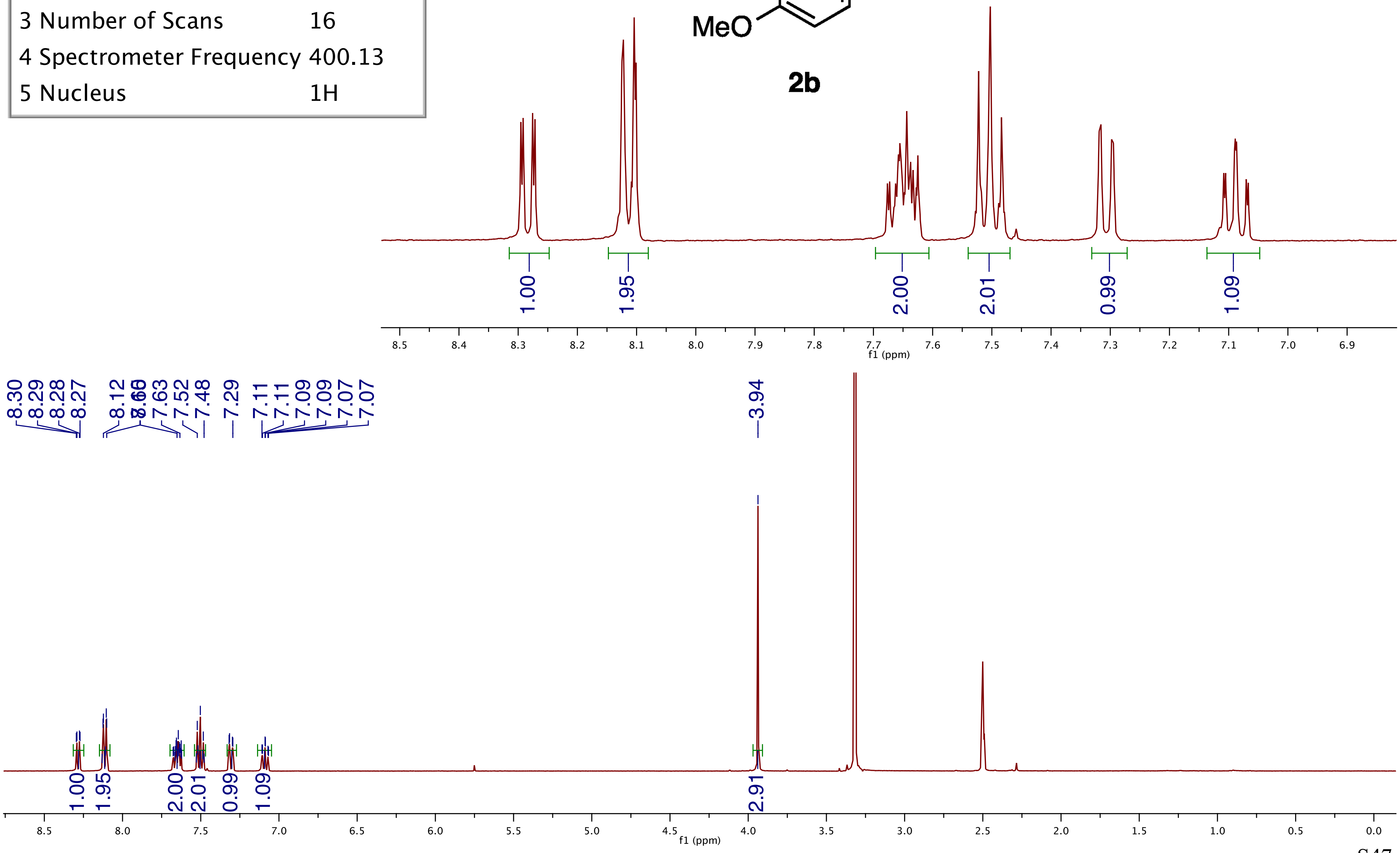

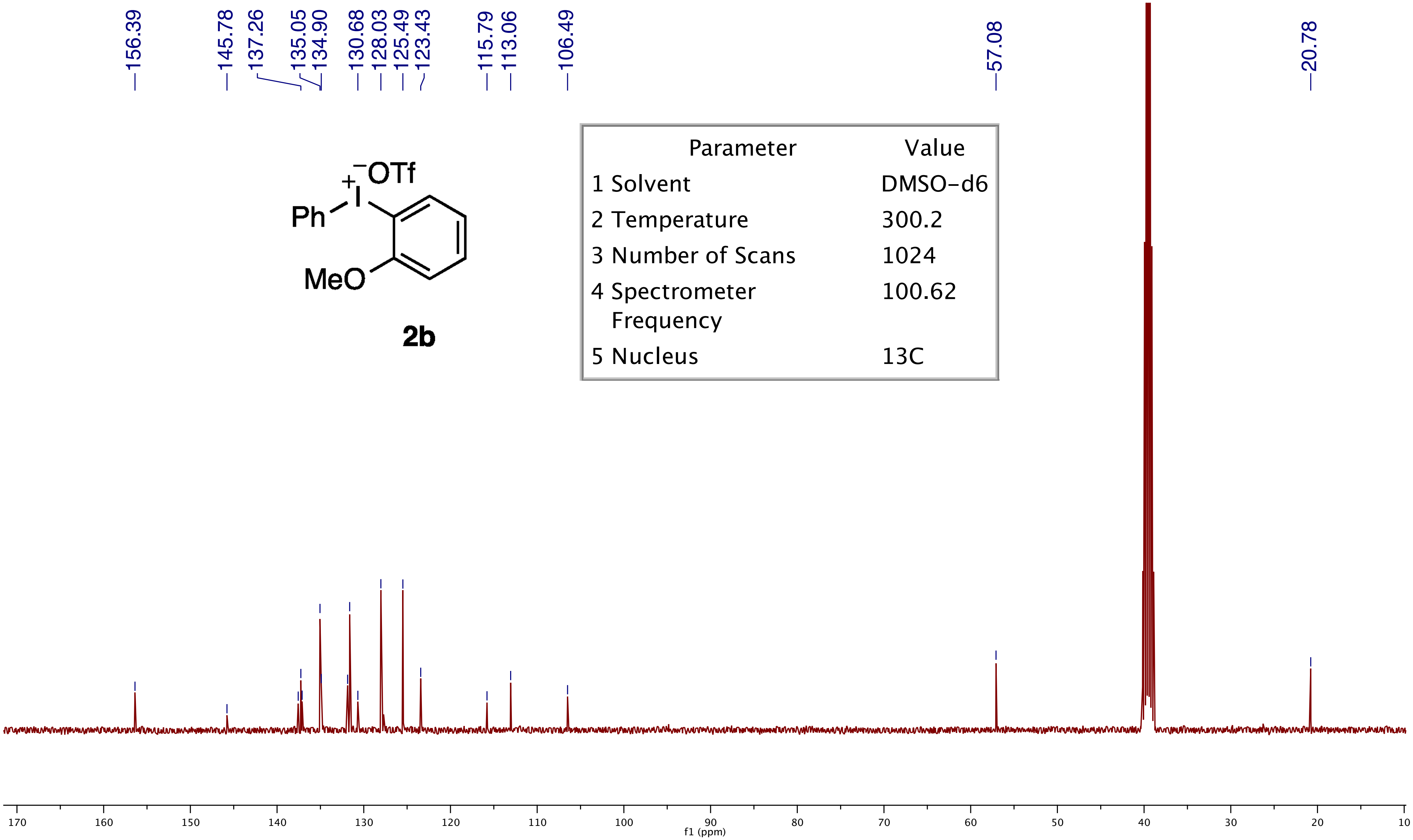

2b

	Parameter	Value
1	Solvent	DMSO-d6
2	Temperature	300.2
3	Number of Scans	1024
4	Spectrometer Frequency	100.62
5	Nucleus	13C

Parameter	Value
1 Solvent	DMSO-d6
2 Temperature	300.1
3 Number of Scans	16
4 Spectrometer Frequency	400.13
5 Nucleus	1H

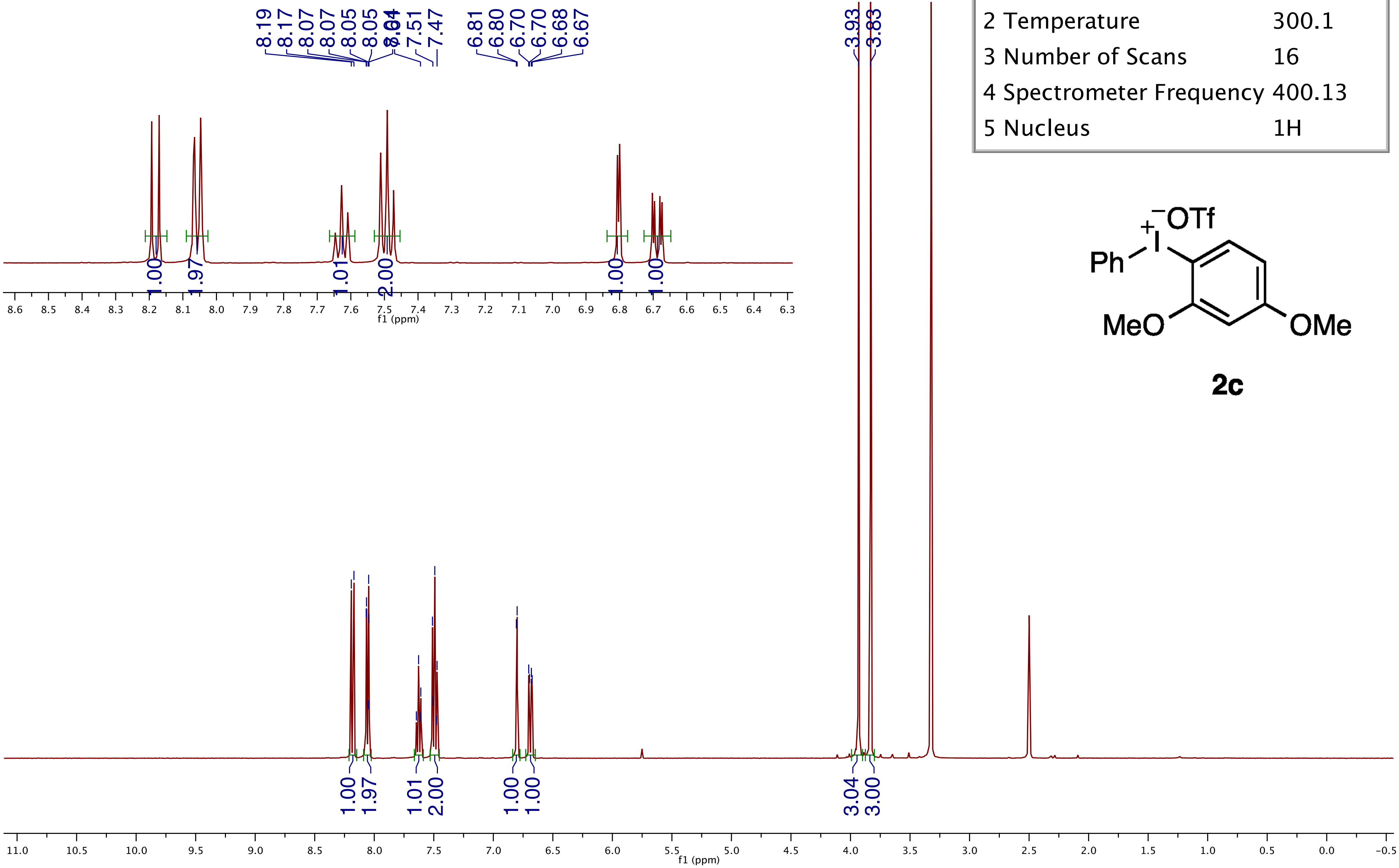

2c

Parameter	Value
1 Solvent	DMSO-d6
2 Temperature	300.2
3 Number of Scans	1024
4 Spectrometer Frequency	100.62
5 Nucleus	13C

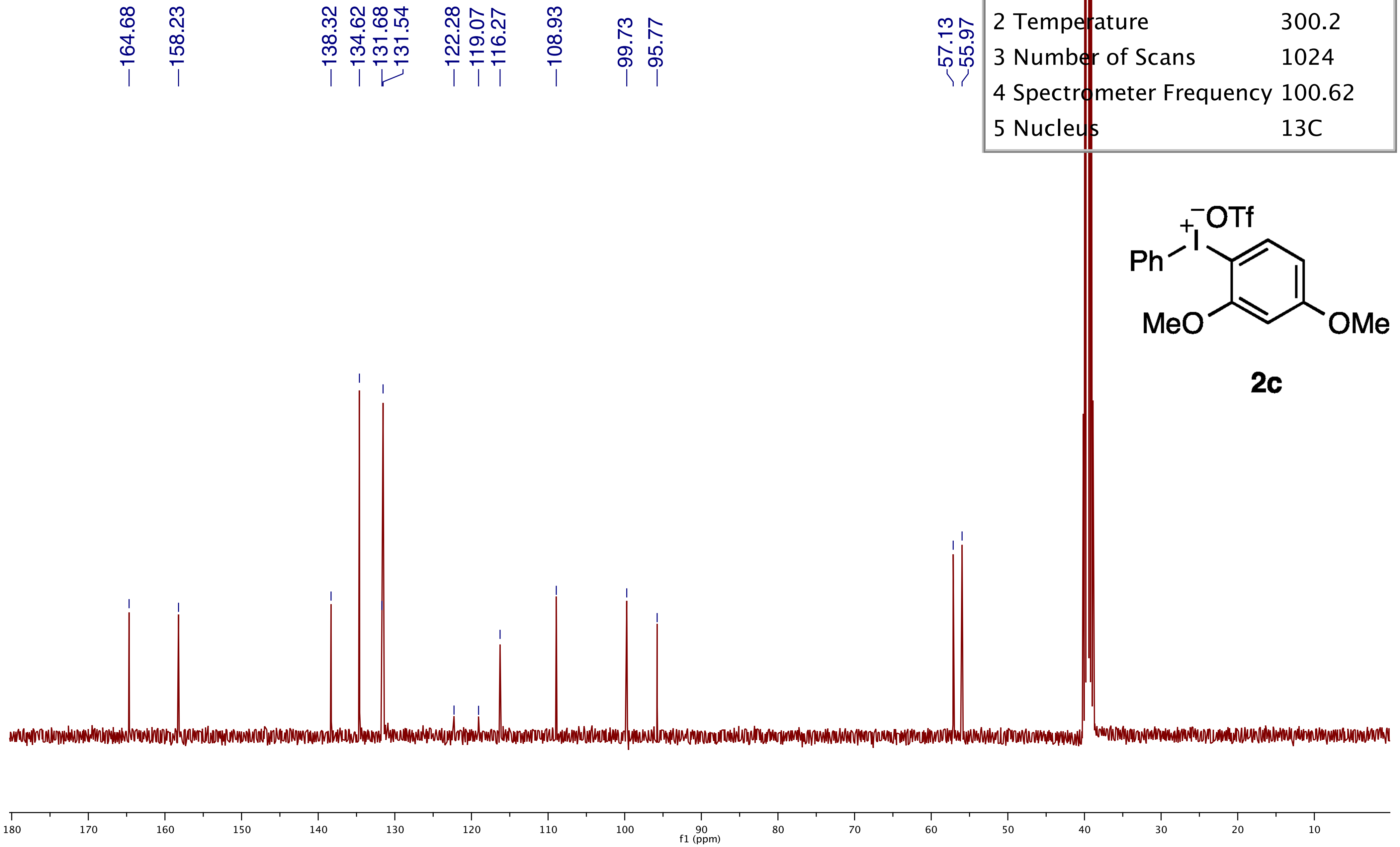

2c

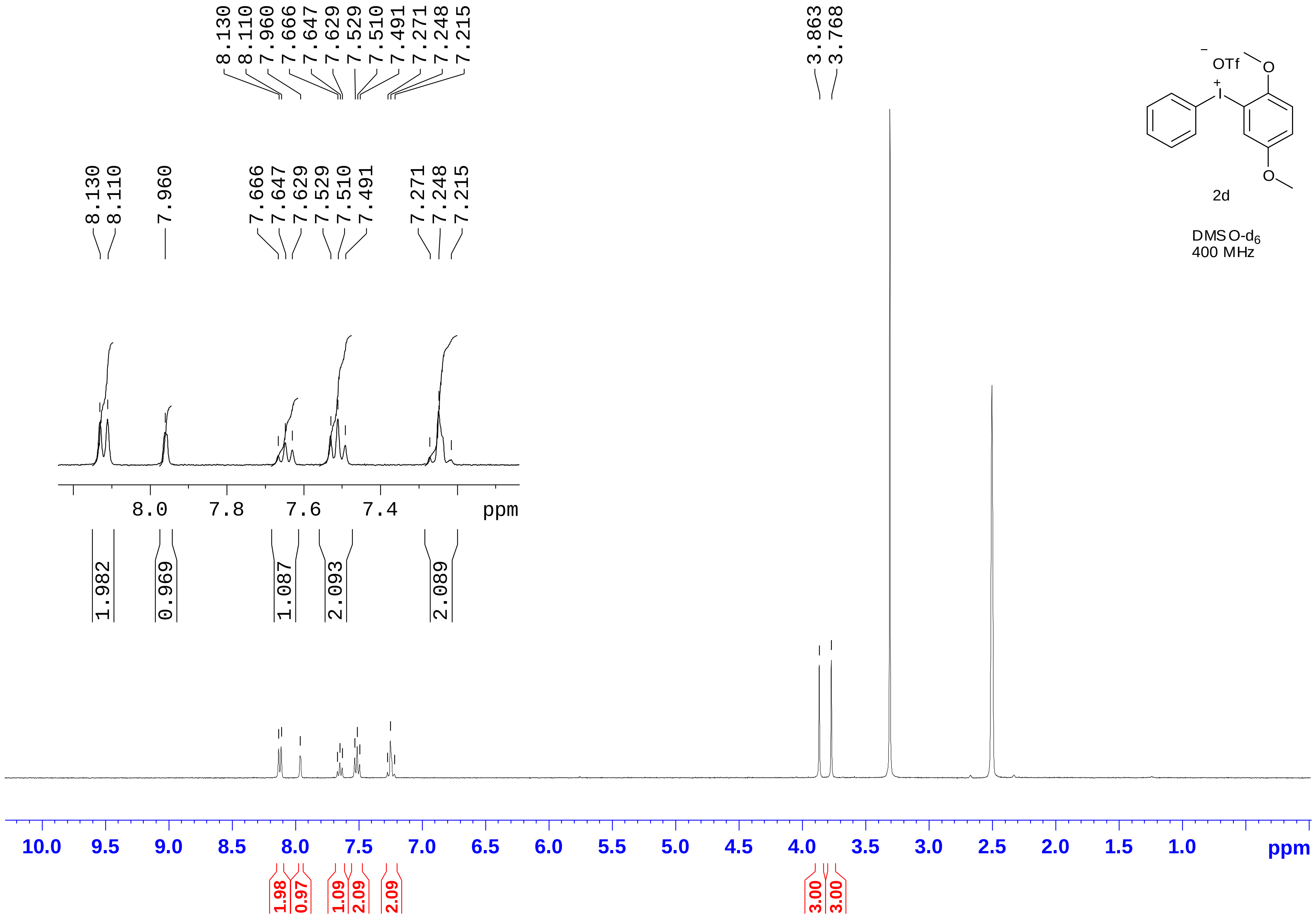
8.130
8.110
7.960
7.666
7.647
7.629
7.529
7.510
7.491
7.271
7.248
7.215
3.863
3.768
OTf
I
O
O
2d
DMSO-d6
400 MHz
8.0
7.8
7.6
7.4
ppm
1.982
0.969
1.087
2.093
2.089
10.0
9.5
9.0
8.5
8.0
7.5
7.0
6.5
6.0
5.5
5.0
4.5
4.0
3.5
3.0
2.5
2.0
1.5
1.0
ppm
1.98
0.97
1.09
2.09
2.09
3.00
3.00

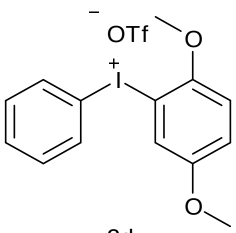

2d

DMSO-d_6
100 MHz

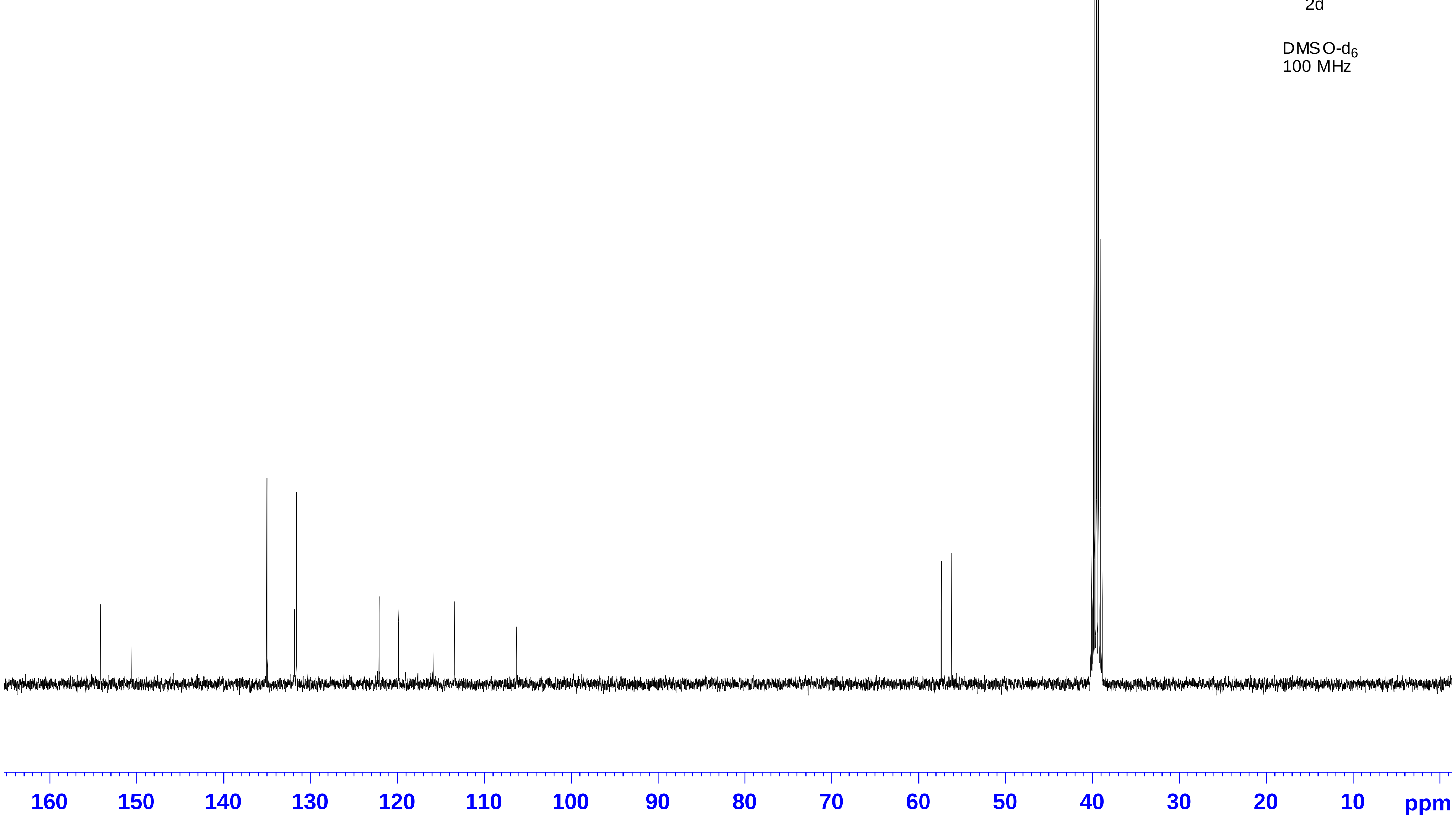

Parameter	Value
1 Solvent	DMSO-d6
2 Temperature	300.2
3 Number of Scans	16
4 Spectrometer Frequency	400.13
5 Nucleus	1H

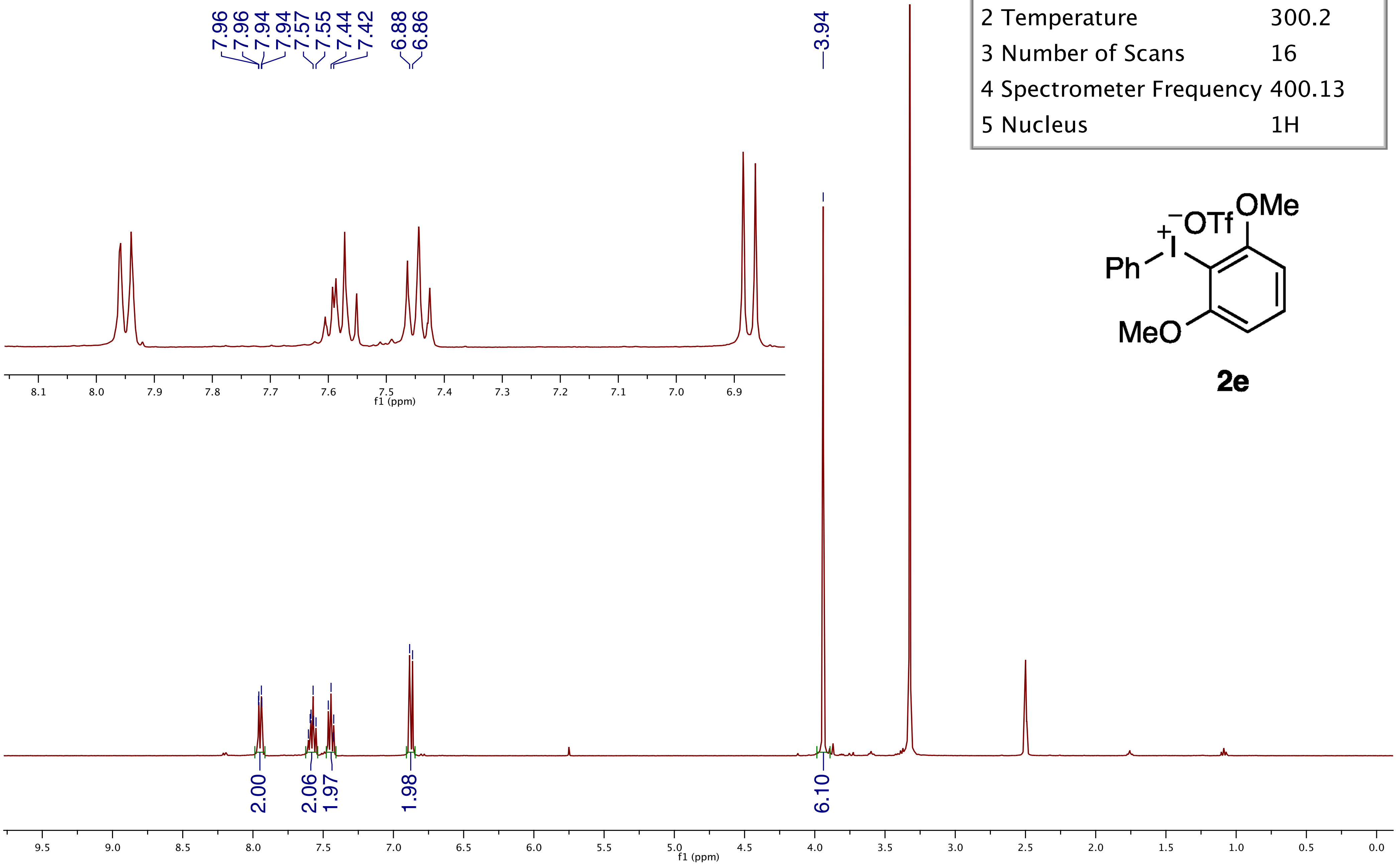

2e

Parameter	Value
1 Solvent	DMSO
2 Temperature	300.2
3 Number of Scans	172
4 Spectrometer Frequency	100.62
5 Nucleus	13C

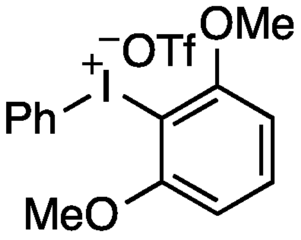

2e

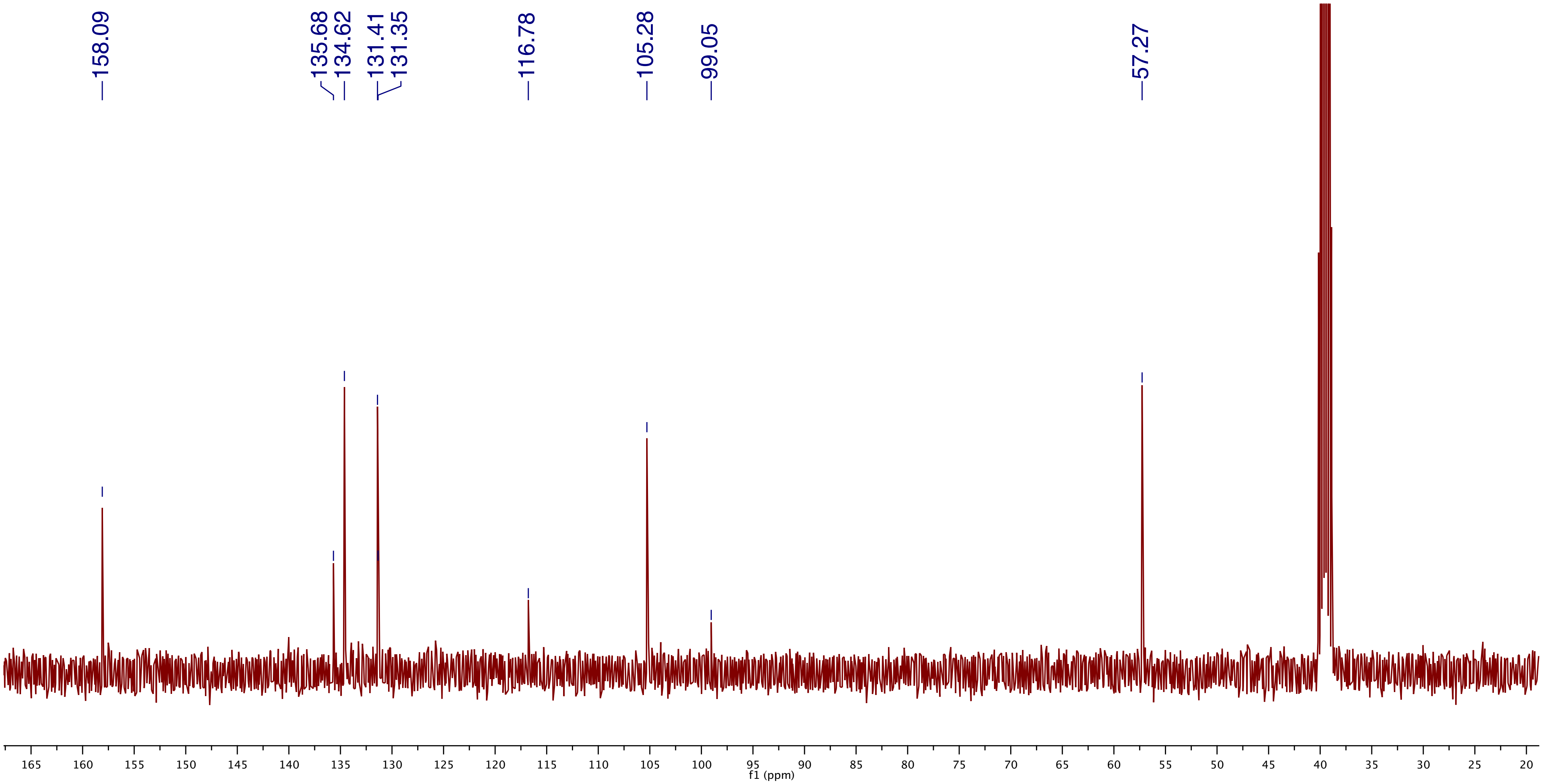

Parameter	Value
1 Solvent	DMSO-d6
2 Temperature	298.2
3 Number of Scans	16
4 Spectrometer Frequency	500.13
5 Nucleus	1H

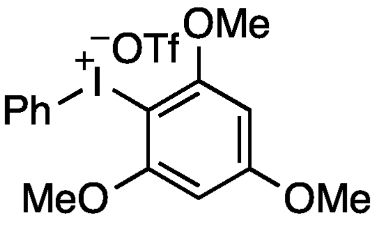

2f

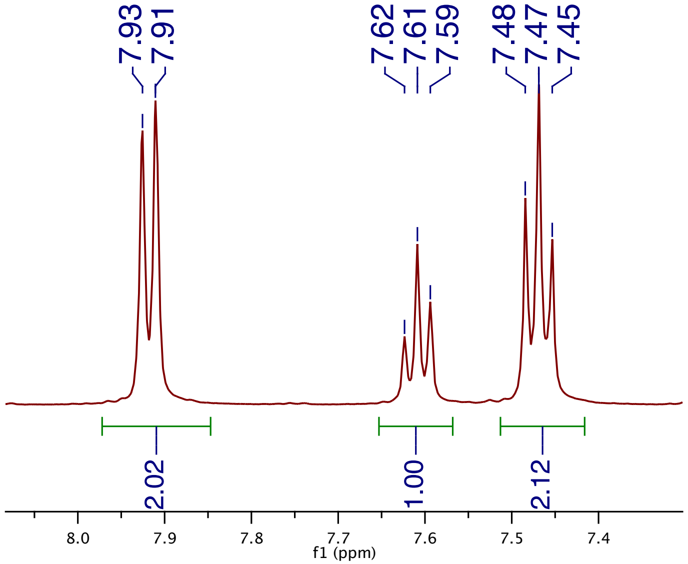

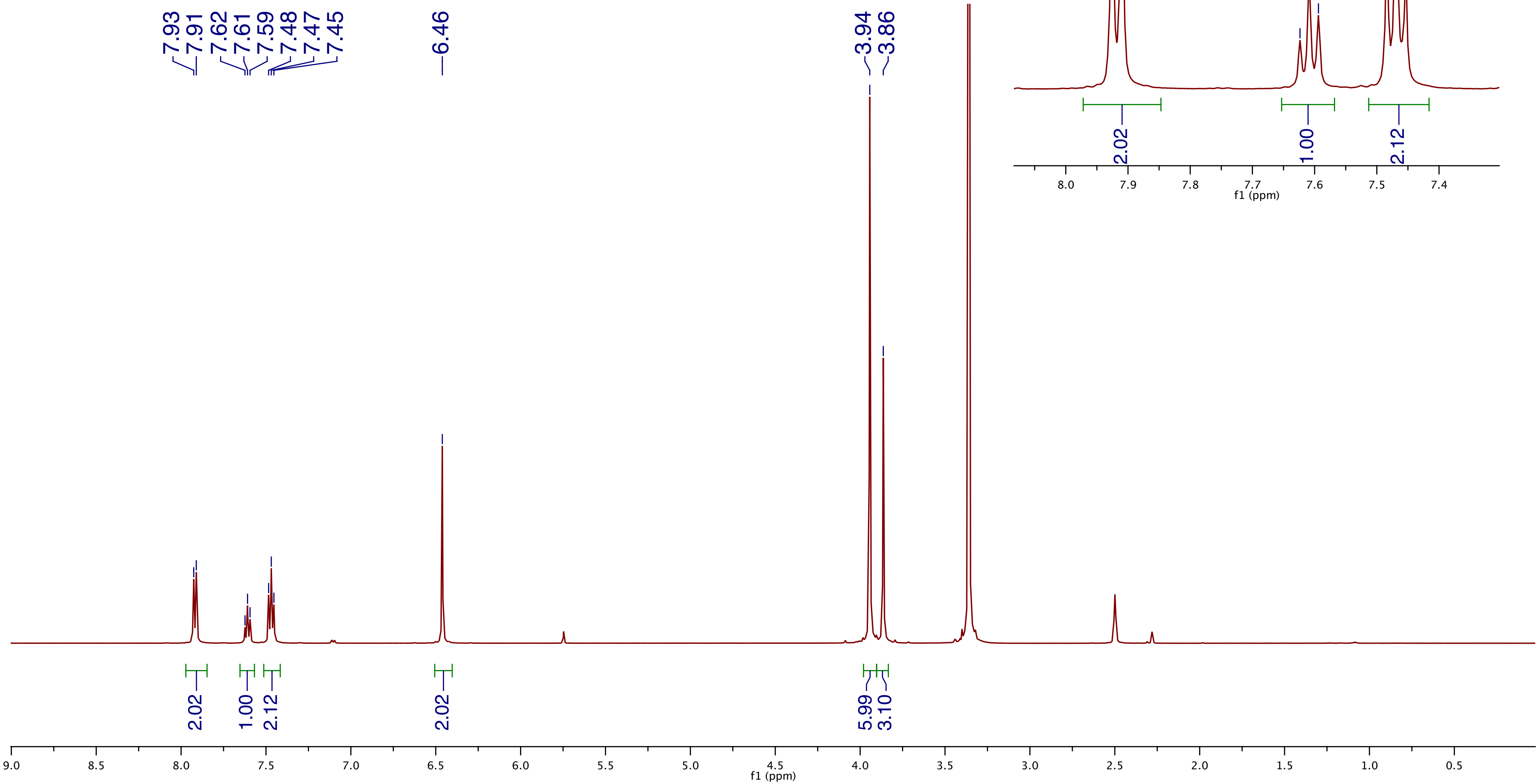

Parameter	Value
1 Solvent	DMSO-d6
2 Temperature	298.1
3 Number of Scans	6144
4 Spectrometer Frequency	100.62
5 Nucleus	13C

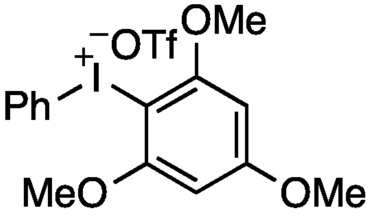

2f

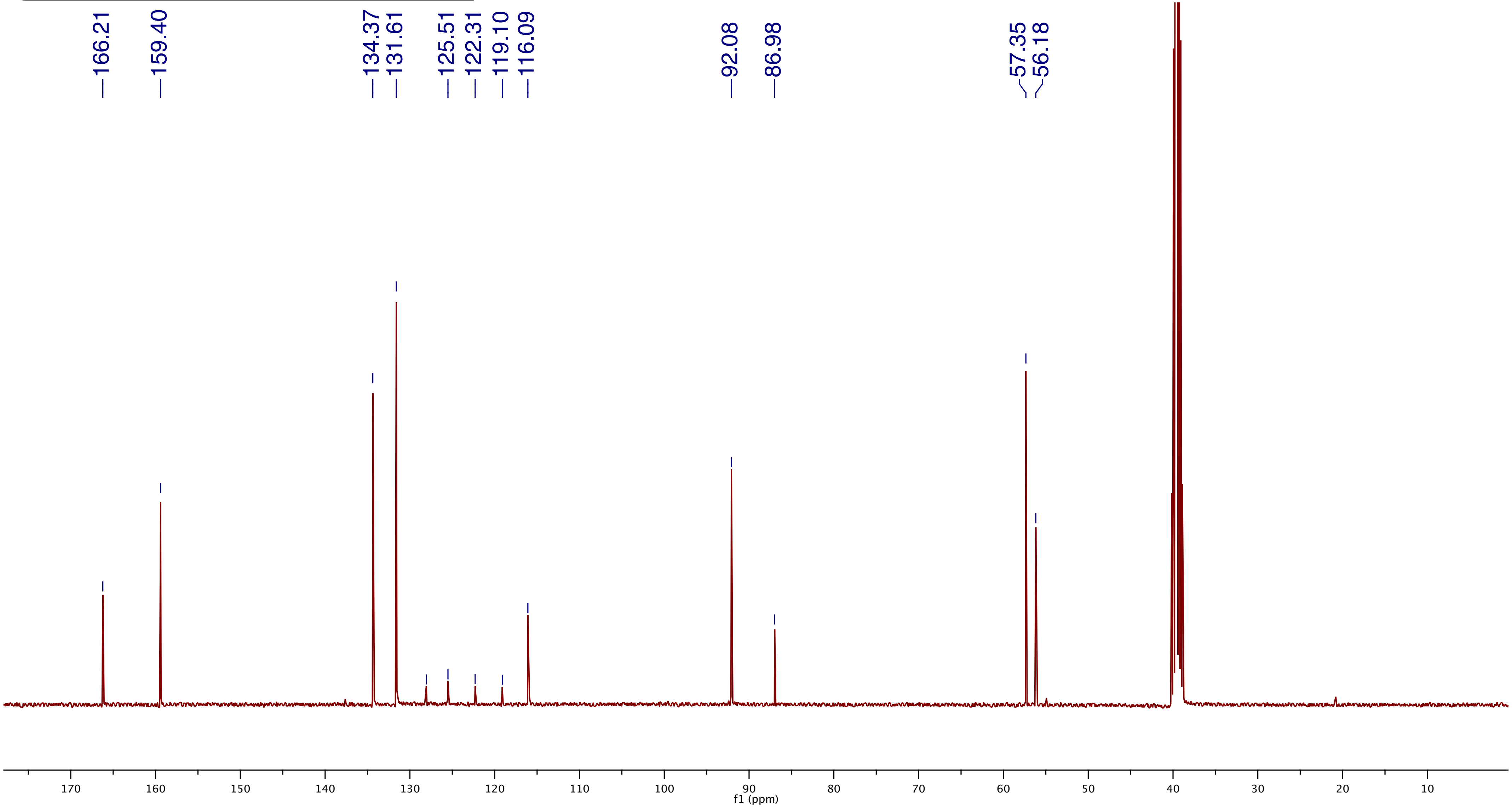

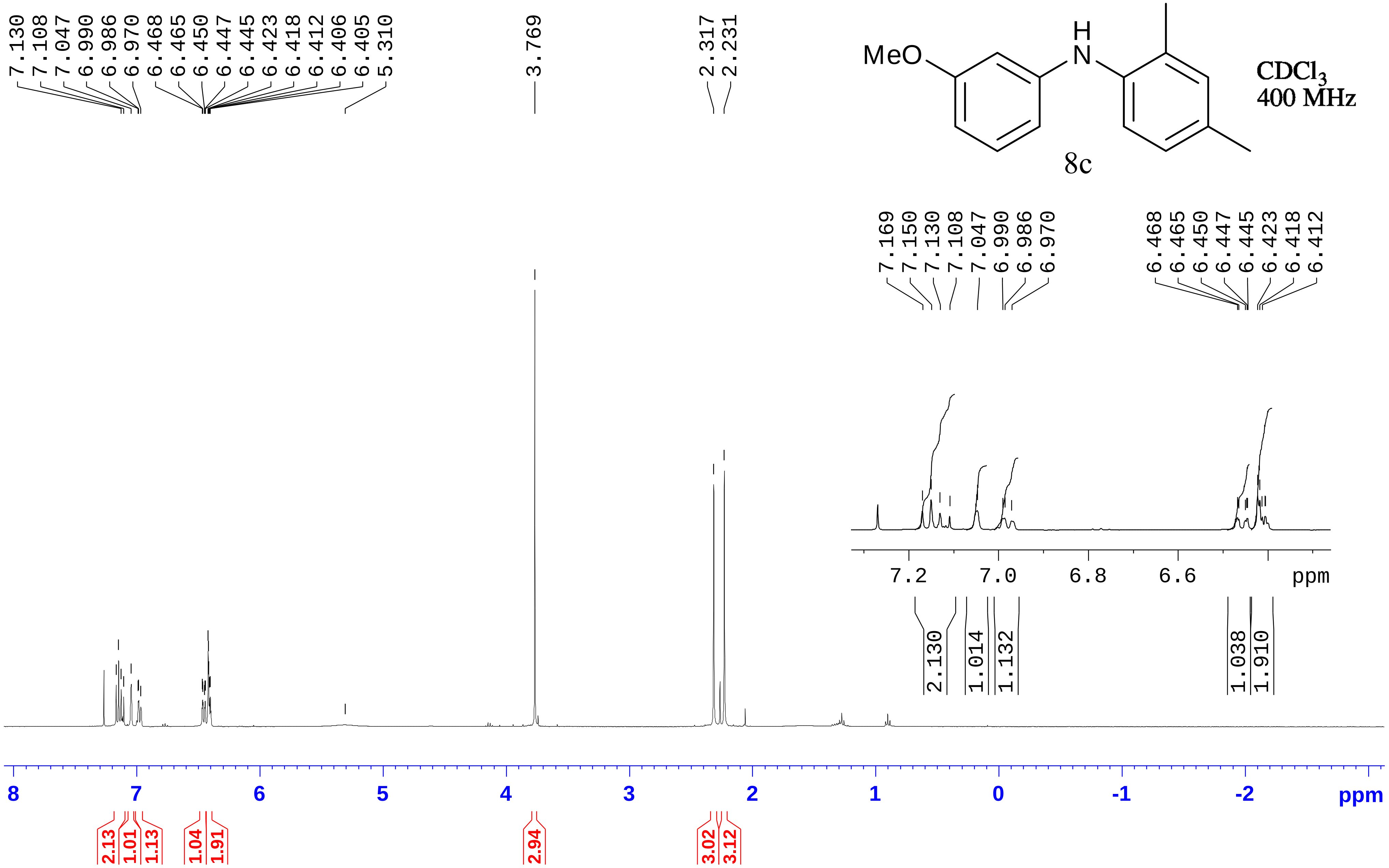
7.130
7.108
7.047
6.990
6.986
6.970
6.468
6.465
6.450
6.447
6.445
6.423
6.418
6.412
6.406
6.405
5.310
3.769
2.317
2.231
MeO
H
N
8c
CDCl3
400 MHz
7.169
7.150
7.130
7.108
7.047
6.990
6.986
6.970
6.468
6.465
6.450
6.447
6.445
6.423
6.418
6.412
7.2
7.0
6.8
6.6
ppm
2.130
1.014
1.132
1.038
1.910
8
7
6
5
4
3
2
1
0
-1
-2
ppm
2.13
1.01
1.13
1.04
1.91
2.94
3.02
3.12

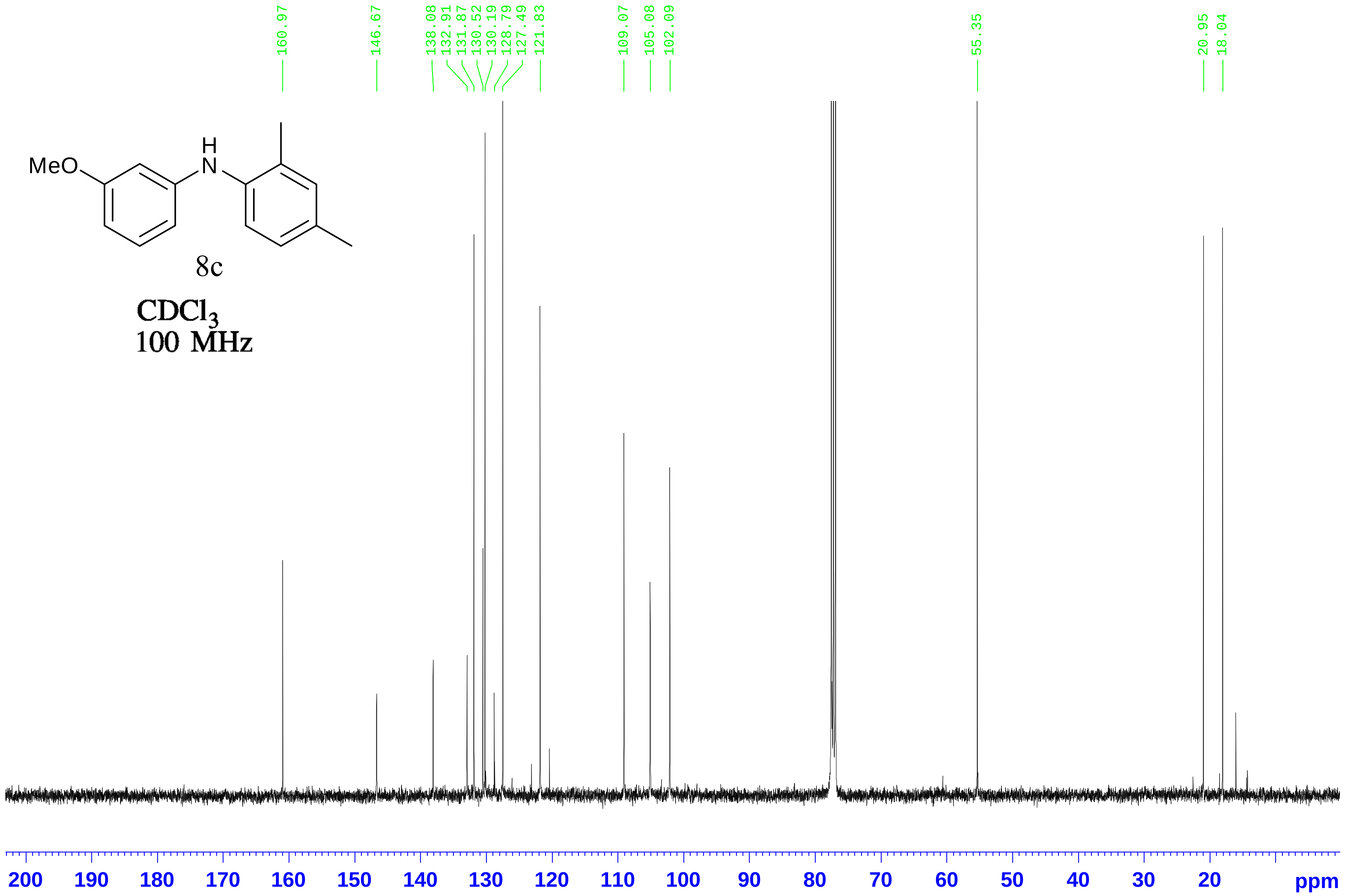
160.97
146.67
138.08
132.91
131.87
130.52
130.19
128.79
127.49
121.83
109.07
105.08
102.09
55.35
20.95
18.04
MeO
H
N
8c
$CDCl_3$
100 MHz
200 190 180 170 160 150 140 130 120 110 100 90 80 70 60 50 40 30 20
ppm

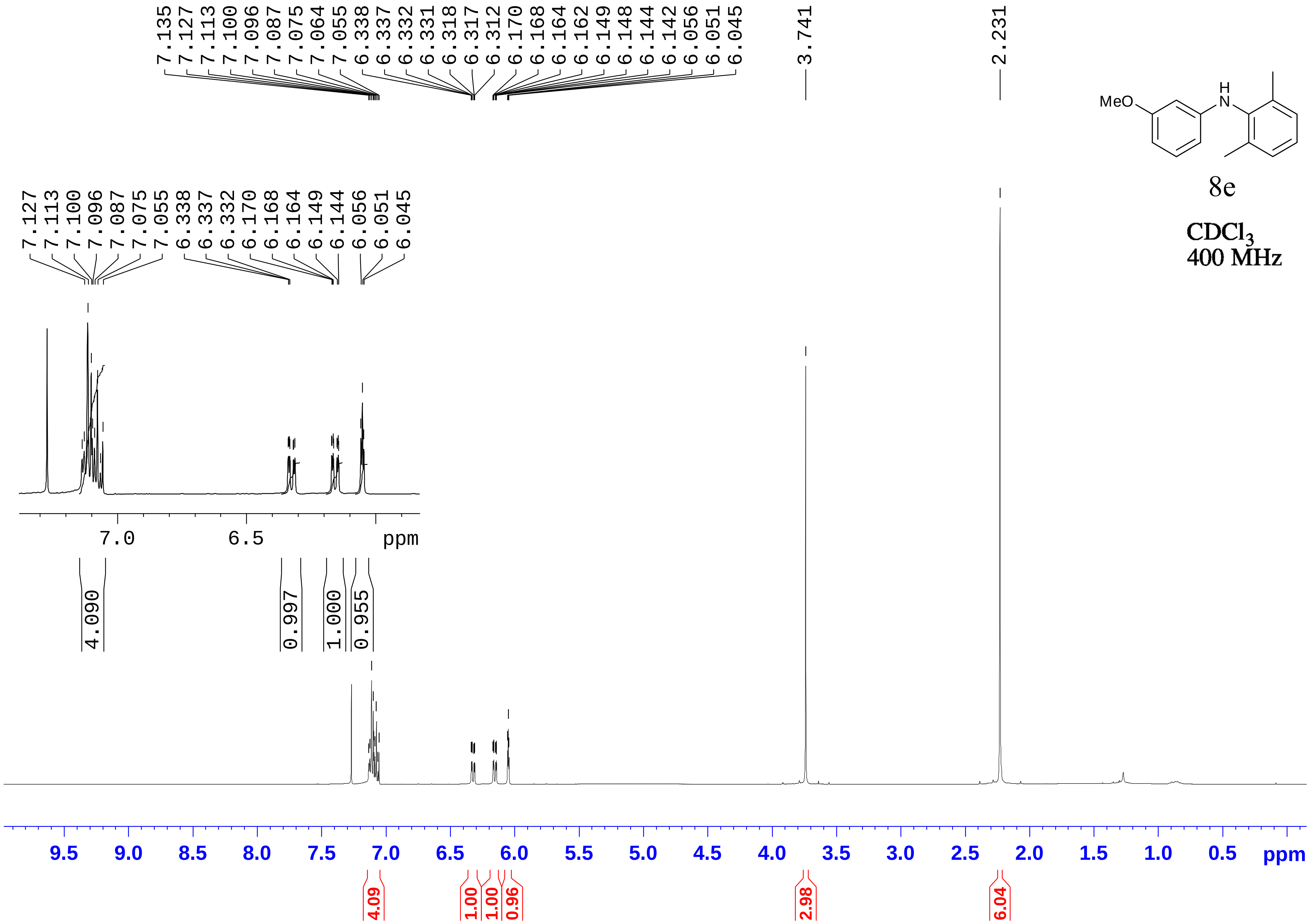
7.135 7.127 7.113 7.100 7.096 7.087 7.075 7.064 7.055 6.338 6.337 6.332 6.331 6.318 6.317 6.312 6.170 6.168 6.164 6.162 6.149 6.148 6.144 6.142 6.056 6.051 6.045
3.741
2.231
MeO
H
N
8e
$CDCl_3$
400 MHz
7.127 7.113 7.100 7.096 7.087 7.075 7.055 6.338 6.337 6.332 6.170 6.168 6.164 6.149 6.144 6.056 6.051 6.045
7.0
6.5
ppm
4.090
0.997
1.000
0.955
9.5 9.0 8.5 8.0 7.5 7.0 6.5 6.0 5.5 5.0 4.5 4.0 3.5 3.0 2.5 2.0 1.5 1.0 0.5 ppm
4.09
1.00
1.00
0.96
2.98
6.04

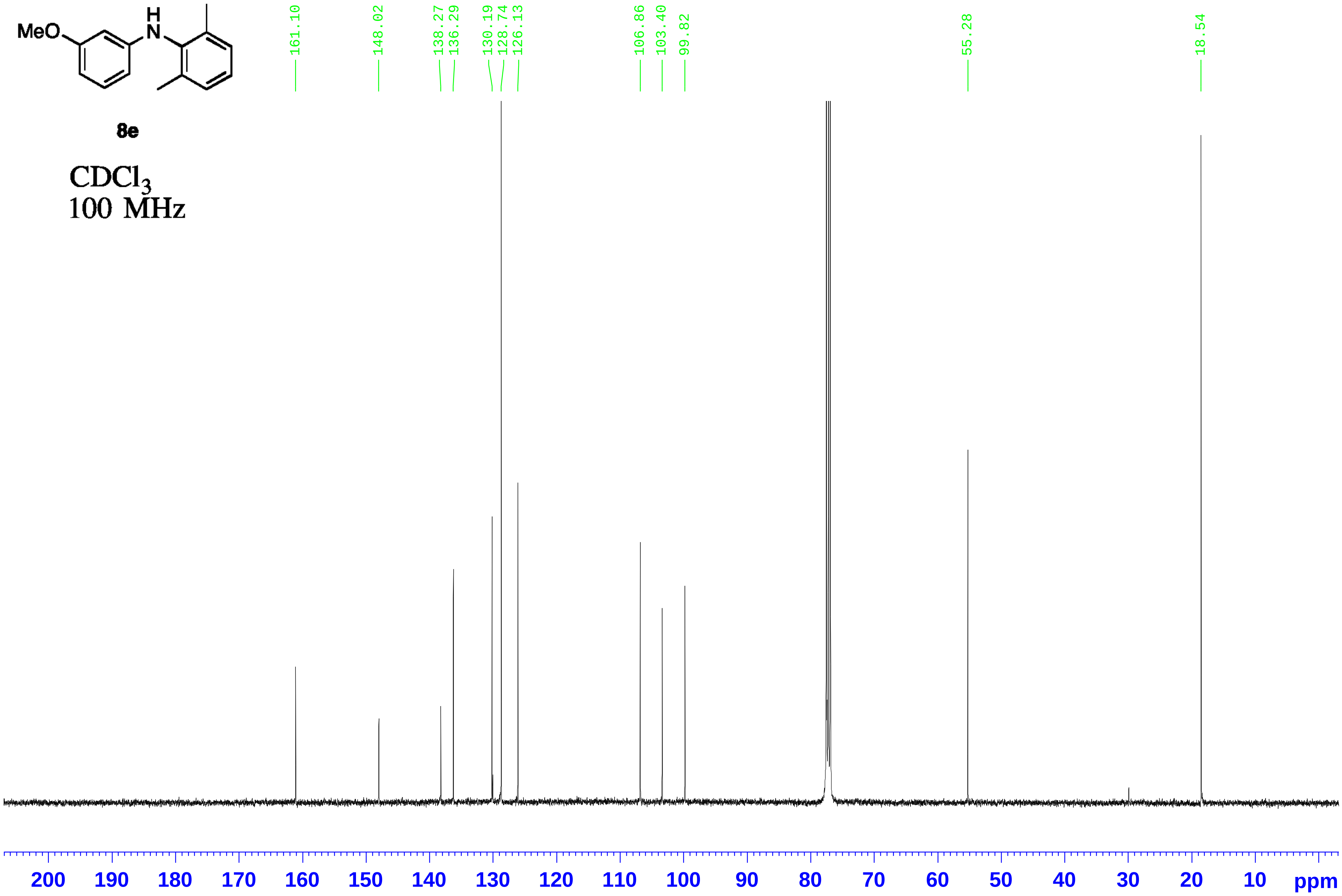
MeO
H
N
8e
CDCl3
100 MHz
161.10
148.02
138.27
136.29
130.19
128.74
126.13
106.86
103.40
99.82
55.28
18.54
200 190 180 170 160 150 140 130 120 110 100 90 80 70 60 50 40 30 20 10 ppm

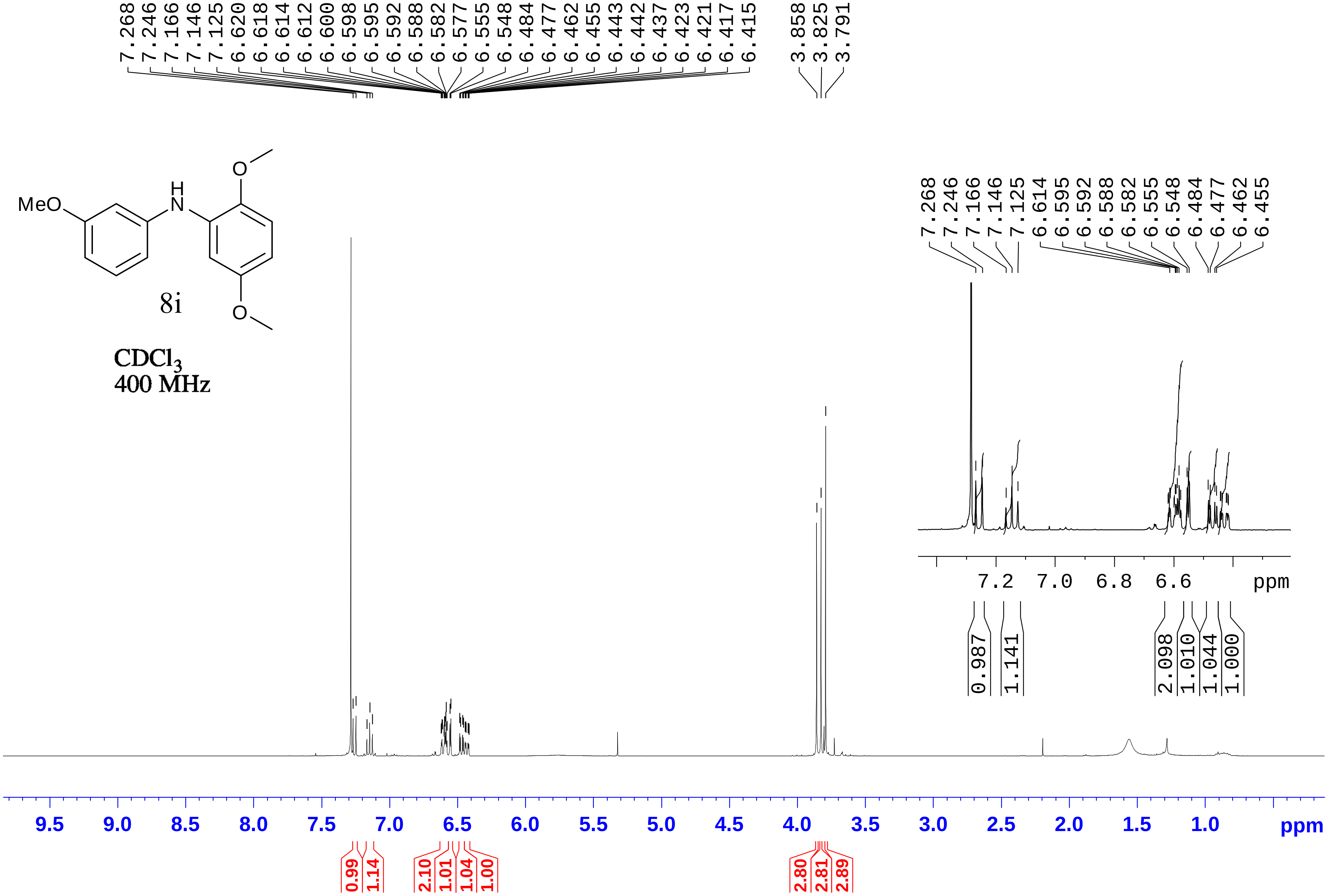

8i

CDCl$_3$
400 MHz

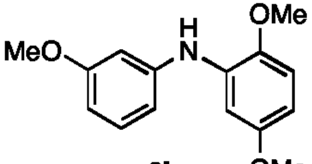

8i

$CDCl_3$
100 MHz

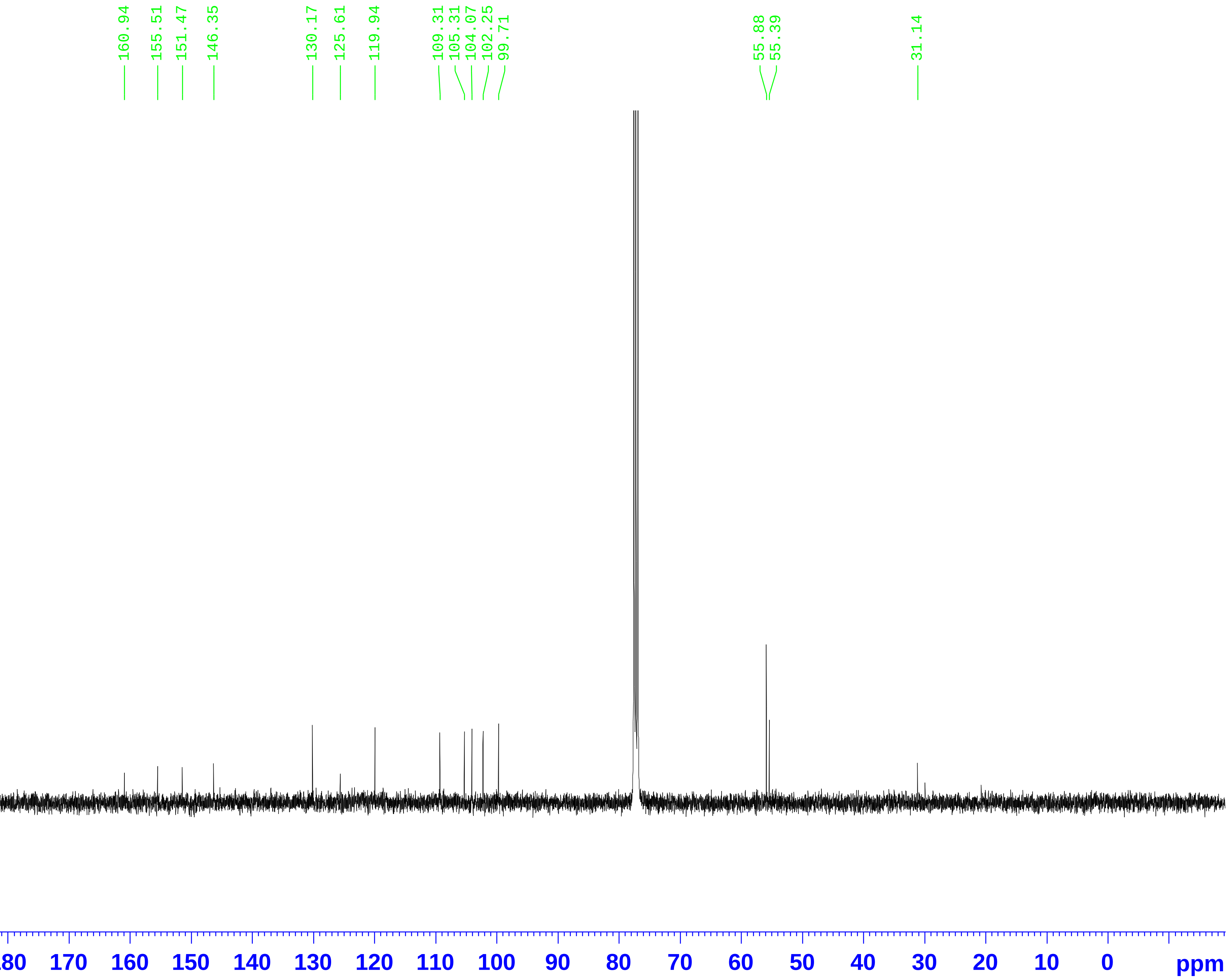

CDCl$_3$
400 MHz

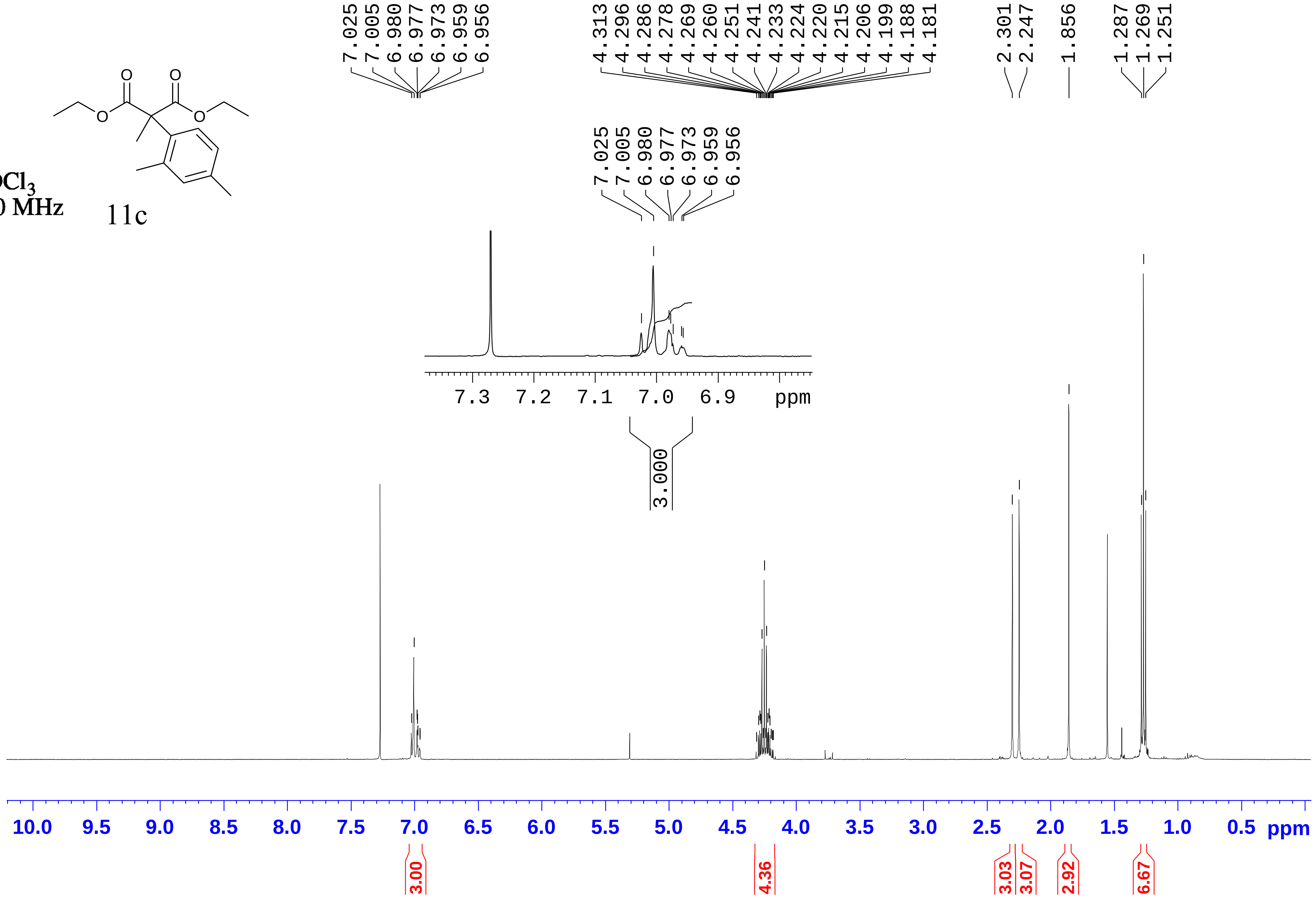

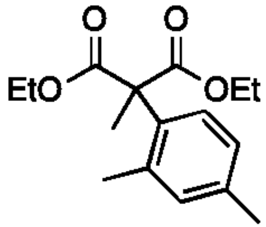

$CDCl_3$
100 MHz

11c

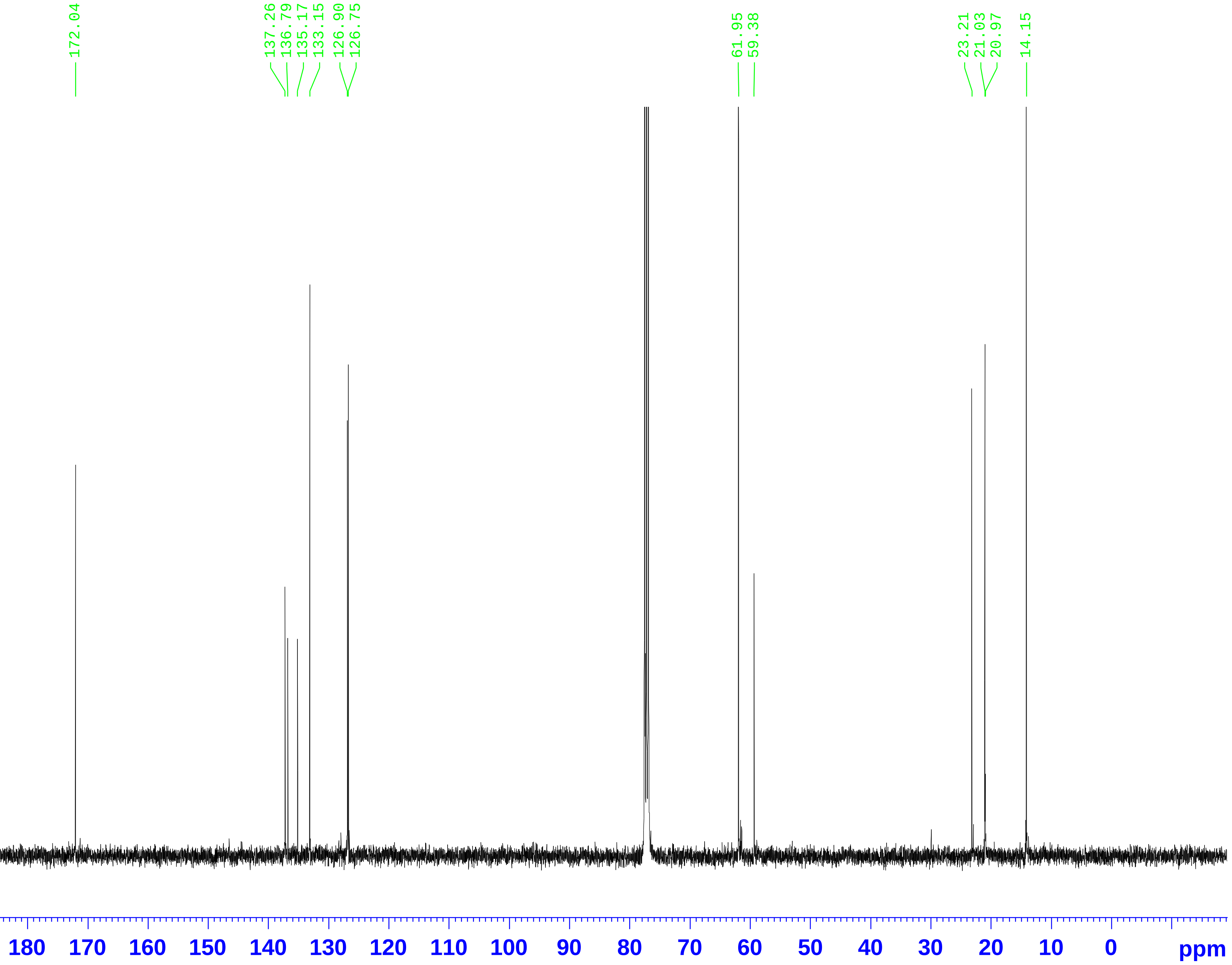

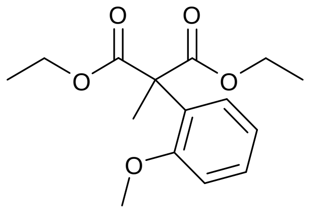

11h

$CDCl_3$
400 MHz

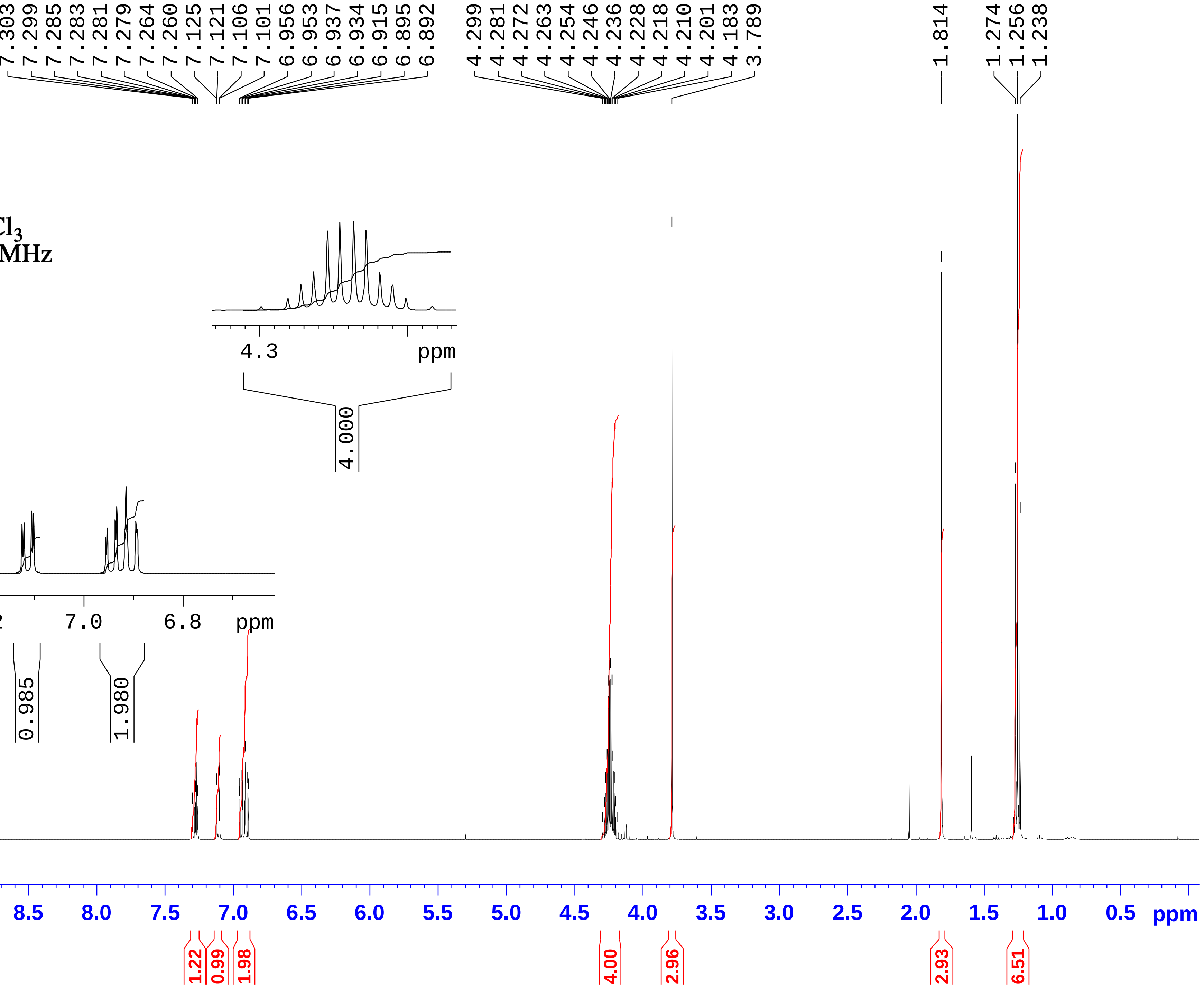

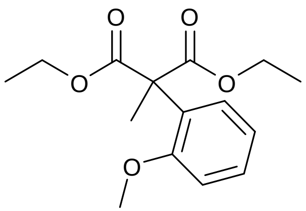

11h CDCl$_3$
100 MHz

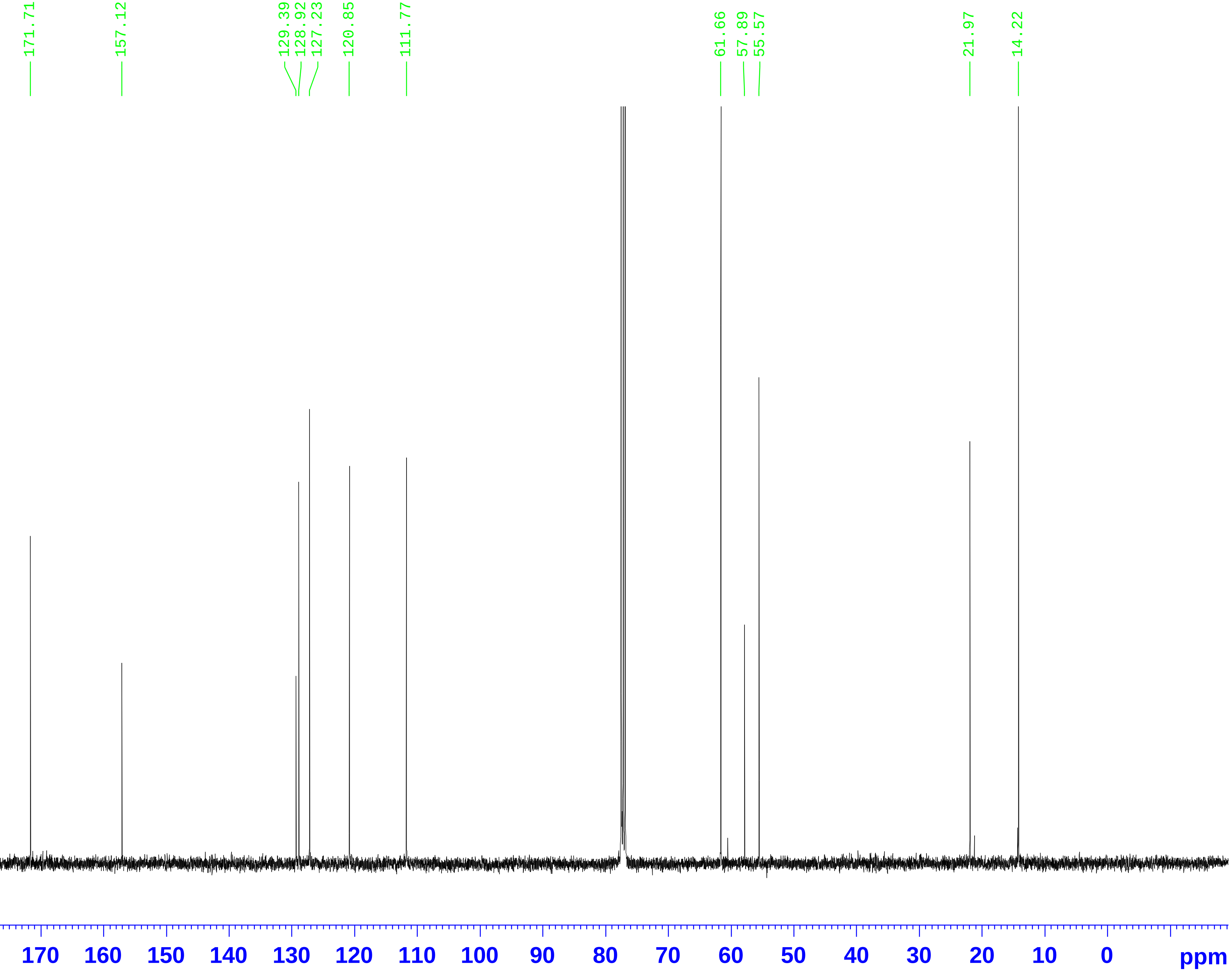

CDCl$_3$
400 MHz

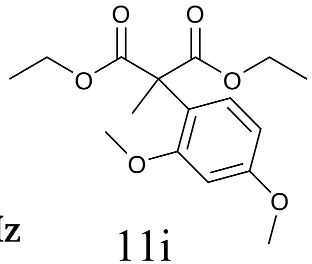

11i

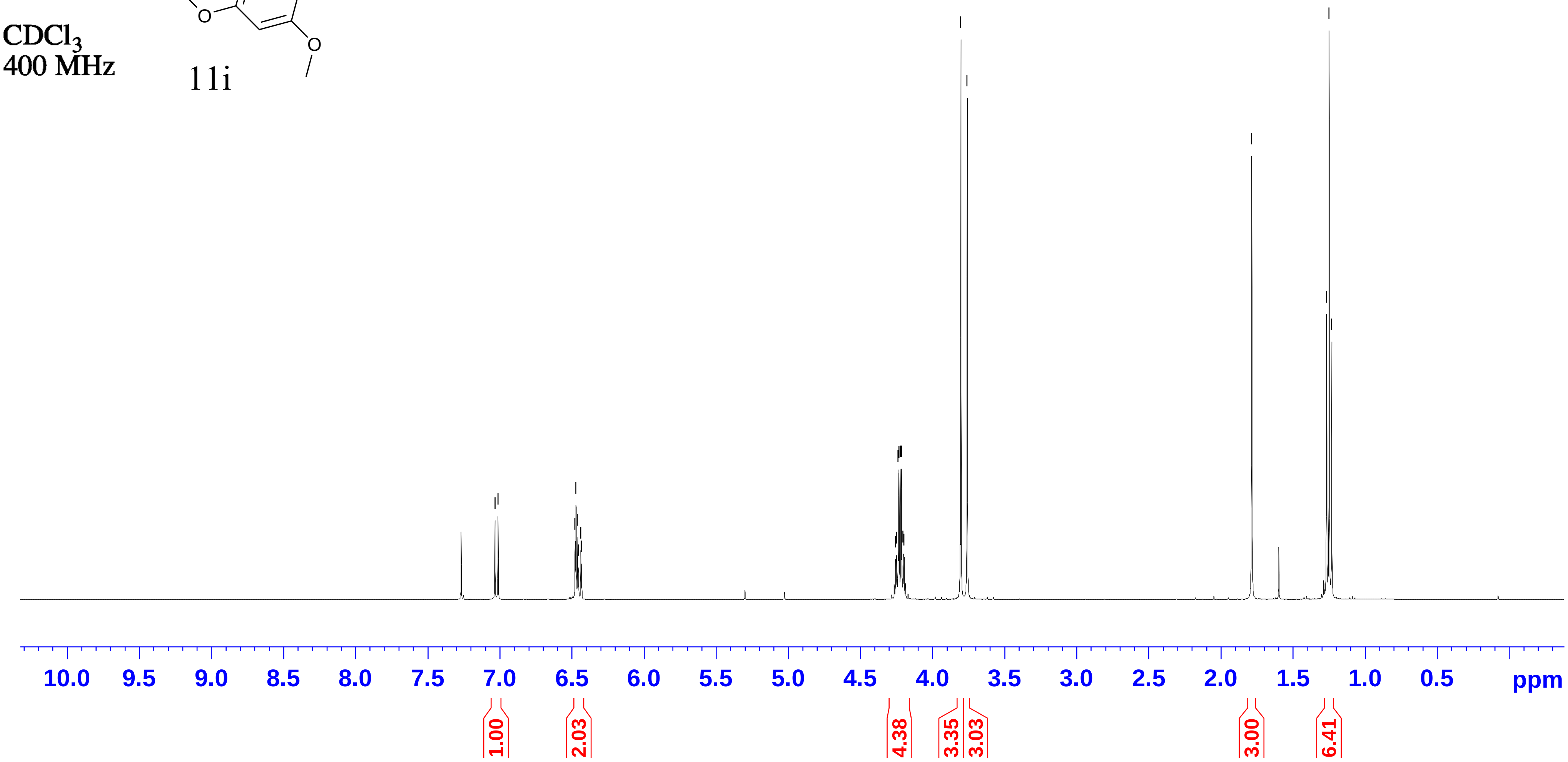

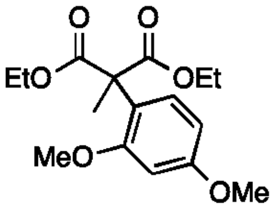

11i

$CDCl_3$
100 MHz

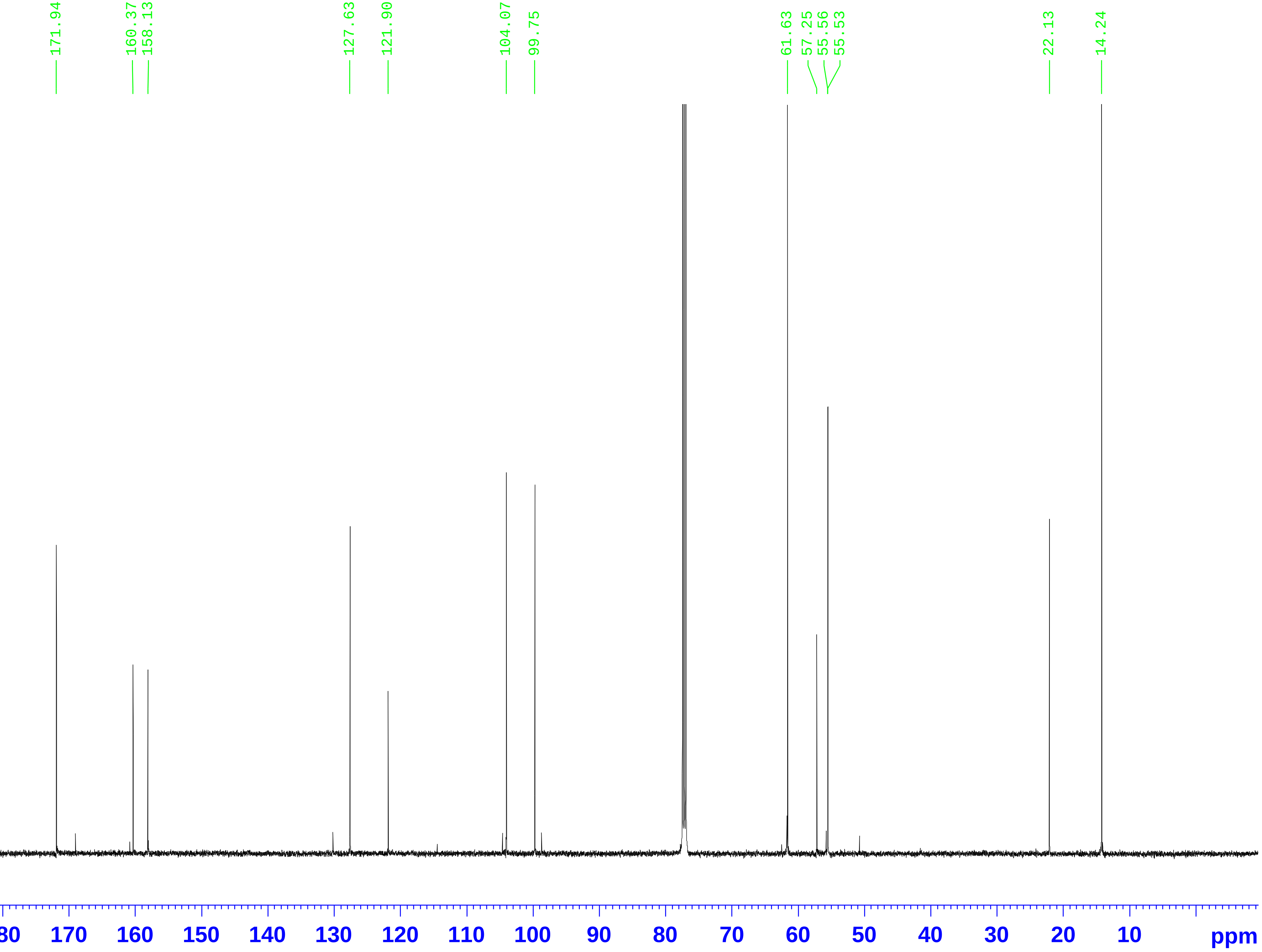

Supplement: Supplementary file 1 [file chem0019-10334-sd1.pdf]
